# Arbutin Ameliorates Murine Colitis by Inhibiting JAK2 Signaling Pathway

**DOI:** 10.3389/fphar.2021.683818

**Published:** 2021-09-14

**Authors:** Liang Wang, Yuntao Feng, Jianwen Wang, Tenglong Luo, Xinyue Wang, Mengze Wu, Runxia Wang, Dapeng Chen, Jiyan Li, Jingyu Wang

**Affiliations:** ^1^ Comparative Medicine Department of Researching and Teaching, Dalian Medical University, Dalian, China; ^2^ Laboratory Animal Center, Dalian Medical University, Dalian, China; ^3^ Department of Spleen and Stomach, Dalian Hospital of Traditional Chinese Medicine, Dalian, China

**Keywords:** dextran sulfate sodium, JAK2, stat3, apoptosis, epithelium, arbutin, inflammatory bowel disease, sulfasalazine

## Abstract

**Background and objective:** Abnormal activation of Janus kinase 2 (JAK2) promotes the pathogenesis and progress of inflammatory bowel disease (IBD) by stimulating the cytokine traffic. Based on docking studies, arbutin, a natural product extracted from a traditional medicinal plant bearberry, was found to bind to JAK2. The study aimed to investigate the effects and mechanisms of regulating JAK2 by arbutin on colitis in mice.

**Methods:** A mice colitis model was established to mimic human IBD. The mice freely drank water containing dextran sulfate sodium. Inflammation in epithelial (IEC6) and immune (RAW264.7) cells was analyzed following treatment with lipopolysaccharides (LPS).

**Results:** Colitis symptoms, including body weight loss, increased disease activity index, and increased colon weight/length ratio, were significantly alleviated by arbutin. Mediators of colonic pro-inflammatory cytokines as well as apoptosis markers in colitis were suppressed by the glycoside. High expression of phosphorylated JAK2 in colitis was significantly reversed by arbutin. The effects of arbutin treatment on colitis were considerably inhibited by the JAK2 inhibitor AG490. LPS-induced inflammatory responses were also suppressed by arbutin, which was notably inhibited by the JAK2 inhibitor AG490.

**Conclusion:** The findings obtained herein suggest the protective role of arbutin and provide novel insights into alternative colitis treatments, which involve inhibition of the JAK2 signaling pathway.

## Introduction

Inflammatory bowel disease (IBD) is a group of nonspecific chronic intestinal inflammatory conditions, including ulcerative colitis (UC) and Crohn’s disease. At present, approximately 3.9 million people are living with IBD worldwide and the number of prevalent cases is continuously increasing (D 2017 Inflammatory Bow, 2020). IBD can cause a series of symptoms, such as diarrhea, abdominal pain and cramps, blood in the stool, and weight loss (Wang et al., 2012). It can also lead to serious extraintestinal complications, including anemia and osteoporosis, which severely affect the patients’ quality of life (Ott and Schölmerich, 2013). Moreover, recurrent IBD is a high risk factor for intestinal tumors (Kim and Chang, 2014). The latest evidence shows that IBD is also closely related to nervous system diseases, such as Parkinson’s disease, Alzheimer’s disease, and depression (Villumsen et al., 2019; Zhu et al., 2019).

The etiology of IBD is complex and is believed to involve complex interactions between genetic, environmental, and microbial factors as well as immune responses (Zhang and Li, 2014). The pathogenesis of IBD includes persistent inflammatory damage caused by injury to the intestinal immune system in genetically susceptible people under the action of environmental factors and intestinal microorganisms (García et al., 2020). In the imbalanced intestinal immune microenvironment of IBD patients, inflammatory mediators, and cytokines released by a large number of inflammatory cells can activate the Janus kinase 2 (JAK2) signaling pathway to aggravate the damage of the intestinal tract and intestinal barrier function. This is related to the destruction of the intestinal epithelial tight junction barrier and apoptosis of intestinal epithelial cells (Li et al., 2010; Prager et al., 2012; Bhat et al., 2019).

JAK2 is an important member of the JAK family, which plays a key role in the cytokine signal transduction. JAK2 binds to the cytoplasmic region of the type II cytokine receptor and is widely distributed in various tissues and cells. Under the action of cytokines, JAK2 is phosphorylated and subsequently activates the downstream signal transducer and activator of transcription 3 (STAT3) in the cytoplasm (Seif et al., 2017). Activated STAT3 dissociates from the upstream tyrosine kinase and enters the nucleus to regulate the expression of various pro-inflammatory genes, thus promoting the development of inflammation (Hillmer et al., 2016).

The objective of IBD treatment is to alleviate excessively activated intestinal inflammatory responses (Pithadia and Jain, 2011). The emergence of different biopharmaceuticals to target cytokines has recently attracted considerable attention in the area of IBD treatment. Based on high oral bioavailability, minimal risk of antibody formation, and low production costs, small molecule drugs have been proposed as promising alternatives to biologic drugs (Lucaciu et al., 2020). Tofacitinib, which acts by inhibiting the activity of JAK, belongs to the group of first approved new generation small molecular drugs for the treatment of moderate to severe UC. However, poor response, disease recurrence, and side effects following the administration of this agent are common (Currie et al., 2019). Hence, research into new IBD treatments has focused on the search for small molecule drugs able to inhibit JAK. Considering the unique role of traditional Chinese medicine in the treatment of IBD (Ke et al., 2012), we previously used molecular docking technologies to screen various monomers isolated from Chinese herbs employing available databases. It was found that arbutin could effectively bind to JAK2. Arbutin is a natural product, which is predominantly extracted from the leaves of bearberry. It is traditionally used to treat urinary tract infections (Garrett, 2003). Nonetheless, the role and specific mechanism of arbutin in intestinal inflammation remains unclear.

The present study aimed to investigate the effect of regulating JAK2 by arbutin on colitis. Mouse colitis and cell inflammation models were established to mimic human UC. The mice freely drank water containing dextran sulfate sodium and the inflammation was analyzed following treatment with lipopolysaccharides (LPS).

## Materials and Methods

### Reagents

Arbutin (497-76-7, ≥ 98%) was obtained from Chengdu Pusi Biotechnology Co., Ltd. (Chengdu, China). Sulfasalazine (SASP) was purchased from Guangdong Qiangji Pharmaceutical Co., Ltd. (Guangdong, China). The Cell Counting Kit-8 (CCK-8) (WLA074b) was acquired from Wanleibio (Shenyang, China). Enzyme-linked immunosorbent assay (ELISA) kits for the detection of IL-1β (26048-1-AP), IL-6 (KE10007), and TNF-α (KE10002) were acquired from Proteintech Co., Ltd. (Wuhan, China). Antibodies against iNOS (14142-1-AP), Bcl-2 (26593-1-AP), and SOCS3 (14025-1-AP) were obtained from Proteintech Co., Ltd. (Wuhan, China). Antibodies against COX-2 (12282), JAK2 (3230), phosphorylated JAK2 (p-JAK2) (4406), and phosphorylated STAT3 (p-STAT3) (9145) were purchased from Cell Signal Co., Ltd. (Boston, MA, United States). Antibodies against GAPDH (ab9485) and MLCK (ab232949) were obtained from Abcam (Hong Kong) Ltd. (Hong Kong, China). Unless otherwise noted, all other reagents were acquired from Sigma-Aldrich (St. Louis, MO, United States).

### Animals

Sixty male C57BL/6 mice (6–8 weeks old, weighing 18–22 g) were obtained from the experimental animal center at the Dalian Medical University [Certificate of Conformity: No. SYXK (Liao) 2018-0007]. The experimental protocol was approved by the Dalian Medical University Animal Care and Ethics Committee (approval number: AEE20046) and was designed to reduce pain and discomfort in the animals. The mice were adapted to laboratory conditions (23°C, 12 h/12 h light/darkness, 50% humidity, free access to food and water) for 1 week prior to conducting the experiments. The mice were raised at a density of one per cage, and were deprived of food for 24 h before euthanasia by cervical dislocation.

### Cell Culture

Rat intestinal epithelial IEC-6 cells and macrophage RAW264.7 cells were obtained from the cell bank of the Shanghai Institute (Shanghai, China). The cells used in the study were evaluated prior to the experiments. No significant interspecific changes in the JAK2 expression, which could affect the outcomes of the study, were found. The cells were maintained at 37°C in a 5% CO_2_ environment. The culture medium consisted of Dulbecco’s Modified Eagle Medium (DMEM) containing 4.5 mg/ml glucose, 50 U/mL penicillin, 50 U/mL streptomycin, 4 mM glutamine, 25 mM 4-(2-hydroxyethyl)-1-piperazineethanesulfonic acid, and 10% fetal bovine serum. Both fetal bovine serum and DMEM were purchased from Invitrogen (Waltham, MA, United States).

### Experiment Design

30 mice were used for pharmacodynamics investigations. The mice were randomized into five groups (*n* = 6). The mice were treated as follows: group I, sham-operated control with intragastric administration of saline; group II, colitis group; groups III, IV, and V intragastric administration of SASP (100 mg/kg body weight dissolved in saline), low-dose arbutin (50 mg/kg body weight dissolved in saline), and high-dose arbutin (100 mg/kg body weight dissolved in saline), respectively, 1 day after induction of colitis. SASP is an anti-inflammatory drug widely used for the clinical treatment of diseases such as IBD. Consequently, it was used as a positive control for analyzing the effects of arbutin on colitis. SASP and arbutin were administered by gavage once daily for seven successive days. In addition to the treatments used for other groups, the mice in group I were given autoclaved drinking water without dextran sulfate sodium (DSS). The colitis symptoms in groups II–V were induced by replacing drinking water with DSS (2.5% w/v) dissolved in autoclaved drinking water.

In the JAK2 blocking experiments, 30 mice were randomly divided into five groups (*n* = 6): group I, sham-operated control with intragastric and intraperitoneal administration of saline; group II, colitis group; groups III, IV, and V treated with high-dose arbutin (100 mg/kg body weight dissolved in saline, intragastric), AG490 (5 mg/kg body weight dissolved in saline, intraperitoneal), and high-dose arbutin + AG490, respectively, 1 day after induction of colitis. The colitis symptoms in groups II–V were induced by replacing drinking water with DSS (2.5% w/v) dissolved in autoclaved drinking water.

Mice in groups III–V were administered high-dose arbutin by gavage and AG490 by intraperitoneal injection once a day for seven consecutive days. Mice in the control group were given autoclaved drinking water without DSS.

Mice in groups I–V were euthanized by cervical dislocation on the 7th day. The distal colon samples were harvested for biochemical studies. The serum was obtained after centrifugation at 1 × 10^3^ g for 10 min at 4°C and was stored at −20°C for ELISA.

### Assessment of Inflammation

The animal body weight as well as the consistency and color of stools for each group were recorded daily. Additionally, the disease activity index (DAI) ratio was scored on the last day. The scores for weight loss, stool bleeding, and stool consistency were added (Kim et al., 2012). The loss of body weight was calculated as the percent difference between the original body weight (day 1) and the body weight on a particular day and scored as follows: 0: no change; 1: ≤5%; 2: 6%–10%; 3: 11–20%; and 4: ≥20%. The stool bleed score was assessed according to the following criteria: 0: none; 2: trace visible blood in stool; and 4: total rectal bleeding. Stool consistency scores were determined as follows: 0: normal; 1: soft but still formed; 2: very soft; 3: half diarrhea; 4: and diarrhea (Dong et al., 2021). The colon weight/length ratio and the spleen index were calculated on the last day. Notably, the colon weight/length ratio and spleen index are morphological parameters used to assess the degree of inflammation in a mouse model of DSS-induced colitis (Lee et al., 2019). The colon samples were dewaxed and dehydrated for hematoxylin eosin (HE) and immunohistochemistry (IHC) staining. The experiments were performed based on previously described methods (Dong et al., 2020). Histology was scored as follows: epithelium (E), 0 = normal morphology; 1 = loss of globlet cells; 2 = loss of globlet cells in large areas; 3 = loss of crypts; 4 = loss of crypts in large areas; and infiltration (I), 0 = no infiltrate; 1 = infiltrate around the crypt basis; 2 = infiltrate reaching the L muscularis mucosae; 3 = extensive infiltration reaching the L muscularis mucosae and thickening of the mucosa with abundant oedema; 4 = infiltration of the L submucosa (Yoshihara et al., 2006). Total histological score was given as E + I. The levels of inflammation-associated proteins were measured by western blotting, while the levels of pro-inflammatory cytokines IL-1β, IL-6, and TNF-α were determined using ELISA kits according to the manufacturer’s instructions.

### Western Blot Analysis

Colon segments were isolated from mice and immediately stored in liquid nitrogen. The total protein was isolated from colonic epithelial cells employing a total protein extraction kit (Beyotime Biotechnology, China). Proteins were transferred onto polyvinylidene fluoride filters and probed with the corresponding antibodies overnight at 4°C with gentle shaking. Bands were detected and quantified using a Bio-Rad ChemiDoc XRS^+^ imaging system (Bio-Rad, Hercules, CA, United States).

### CCK-8 Assay

The effects of arbutin on the viability of IEC-6 and RAW264.7 cells were measured using the CCK-8 assay. Briefly, 5 × 10^3^ cells in the logarithmic growth phase were seeded in 100 μL of DMEM medium containing 10% FBS in each well of a 96-well plate. The cells were treated with arbutin at the specified concentration for 24 h. Then, 10 μL of CCK-8 solution was added to the cells, which were then incubated at 37°C for 4 h. An automatic microplate reader (Thermo Fisher Scientific, Waltham, MA, United States) was used to measure the absorbance at 450 nm to determine cell viability.

### Immunofluorescent Analysis

RAW264.7 cells were inoculated into glass slides, and added into 6-well culture plates. The cells were subsequently allowed to adhere overnight prior to treatment with LPS (1 μg/ml) for 12 h to induce the colitis model. AG490 and arbutin were then administered for 12 h to analyze the effect of the latter on colitis. The glass slides containing RAW264.7 cells were inoculated and washed three times with ice-cold phosphate-buffered saline (PBS). The cells were fixed with 4% paraformaldehyde, incubated in 0.1% Triton X-100 (Amresco) in PBS, and stained with rabbit anti-p-STAT3 (1:500 dilution) in 1% BSA (Solarbio) overnight at 4°C. After washing three times with PBS, the cells were incubated for 60 min at 37°C in the presence of anti-rabbit secondary antibodies (Cell Signaling Technology Inc.). The cells were treated with ProLong Gold antifade reagent containing 4′,6-diamidino-2-phenylindole (DAPI) (Invitrogen). The images of the treated cells were acquired by trichromatic immunofluorescence microscopy using blue (DAPI) and fluorescent (fluorescein) filters with 20 × and 40 × targets, respectively.

### Flow Cytometry Analysis

The antiapoptotic effect of arbutin was evaluated in IEC-6 cells by flow cytometry. The IEC-6 cells were added into 6-well culture plates and allowed to adhere overnight. The cells were subsequently treated with LPS (1 μg/ml) for 12 h to induce the IEC-6 cell apoptosis model. AG490 (50 μM) and arbutin (500 μM) were then administered for 12 h to investigate the effect of arbutin on the apoptosis of IEC6 cells. Untreated IEC-6 cells were used as controls. Following digestion with trypsin in the absence of ethylenediaminetetraacetic acid (EDTA) for 2 min, the IEC-6 cells were collected and washed three times with a washing buffer. The cells in each group were counted, equilibrated, and then stained with annexin V-fluorescein isothiocyanate (FITC) and propidium iodide (PI) according to the manufacturer’s protocol (annexin V-FITC apoptosis detection kit, BD Pharmingen, United States). The apoptotic cells (annexin V-positive and PI-negative) were quantitatively evaluated by flow cytometry according to the manufacturer’s procedure (BD Pharmingen, United States).

### Molecular Docking

Molecular docking was conducted in MOE1. The crystal structure of JAK2 was downloaded from the RCSB Protein Data Bank (PDB ID: 4GL9) and defined as the receptor. The crystal structure of arbutin was acquired from the SPECS database (STOCK1N-07141) and defined as the ligand. The ligand binding site in the original crystal structure of JAK2 was used as the binding site for docking. Prior to docking, the force field of AMBER12: EHT and the implicit solvation model of the Reaction Field were selected. The protonation state of the protein as well as the orientation of the hydrogen atoms were optimized by the LigX module at the pH of 7 and temperature of 300 K.

### Statistical Analysis

The animal experiments, *in vitro* experiments, and data analyses were conducted according to a single-blind study design. Data between three or more groups were compared using one-way analysis of variance, while data between two groups were compared by the student’s t-test. Data were expressed as mean ± standard deviation. Data were normally distributed and each group showed similar variances. Further evaluations were performed using the Kruskal–Wallis rank-sum tests. All experiments were repeated at least three times and a *p*-value of < 0.05 was considered statistically significant.

## Results

### Toxicology of Arbutin *in vitro* and *in vivo*

**FIGURE 1:**
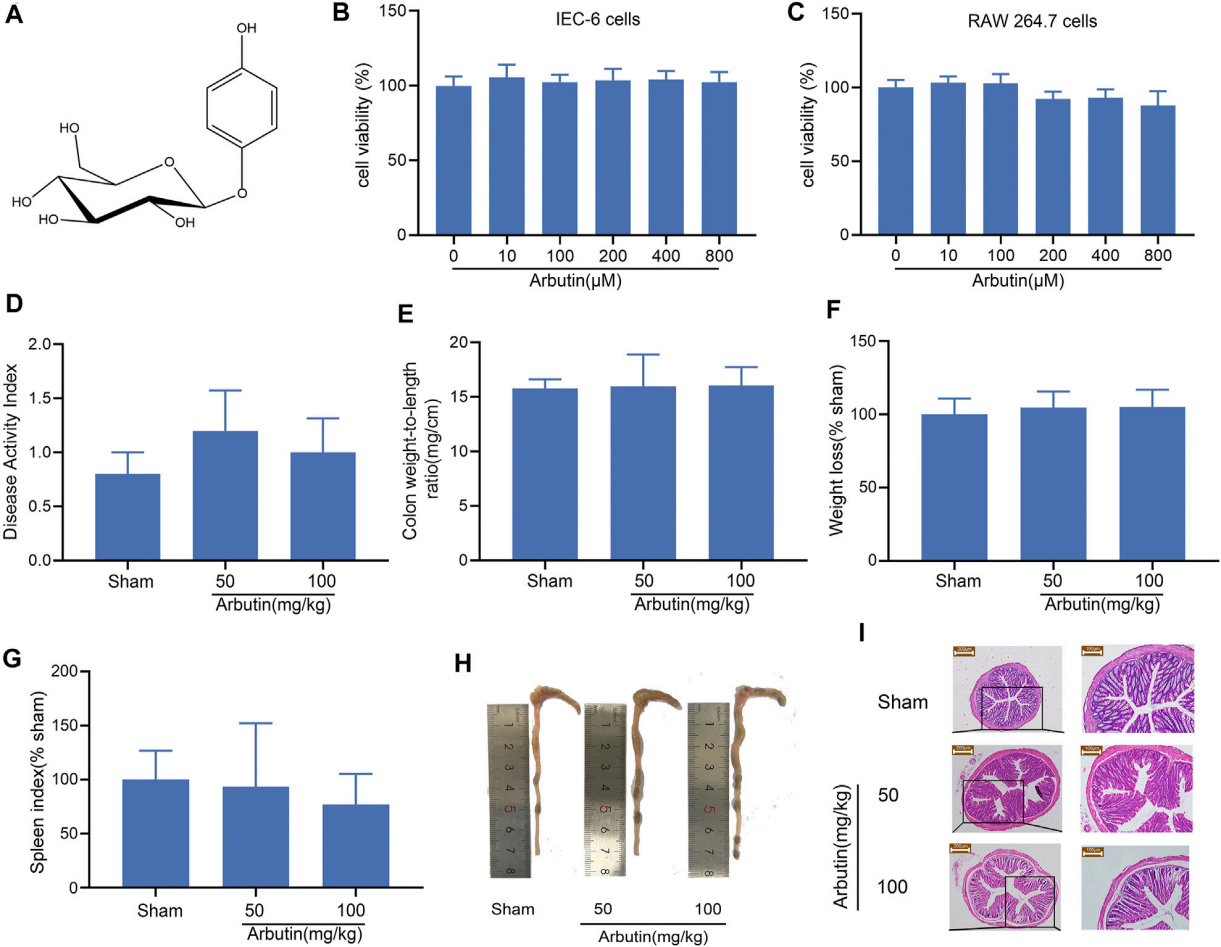
Toxicology of arbutin *in vitro* and vivo **(A)** Chemical structure of arbutin. **(B**, **C)** IEC-6 cells and RAW-264.7 cells were treated with CCK-8 solution for 4 h after pretreatment with arbutin (10, 100, 200, 400, and 800 μM) for 24 h. The cell viability was investigated by CCK-8. The mice were given arbutin (50 and 100 mg/kg) intragastrically once a day for 7 consecutive days **(D)** DAI, **(E)** colon weight-to-length ratio, **(F)** body weight, **(G)** spleen index, and **(H)** macroscopic observation **(I)** Hematoxylin-eosin staining of mouse colonic tissue. Scale bars, 200 μm (left panel) and 100 μm (right panel). Data are expressed as the mean ± SD. *n* = 3 samples.

The chemical structure of arbutin is shown in Figure 1A. The cytotoxicity of the natural product was examined *in vitro* by exposing the cells to 10–800 μM arbutin for 24 h. These concentrations did not affect the viability of IEC-6 (Figure 1B) or RAW264.7 cells (Figure 1C). Intragastric administration of arbutin at a dose of 50 and 100 mg/kg for seven consecutive days had no effect on the DAI, colon weight/length ratio, mouse body weight, spleen index, or colon length (Figures 1D–H) in the colon tissue. Moreover, the HE images exhibited no significant differences between the mice in the sham group and those treated with arbutin (Figure 1I). Based on these preliminary experiments and previous reports (Ye et al., 2019; Ding et al., 2020), the maximum therapeutic dose of arbutin was set to 100 mg/kg (*in vivo*) and 500 μM (*in vitro*).

### Arbutin Alleviates DSS-Induced Colitis Symptoms in Mice

**FIGURE 2:**
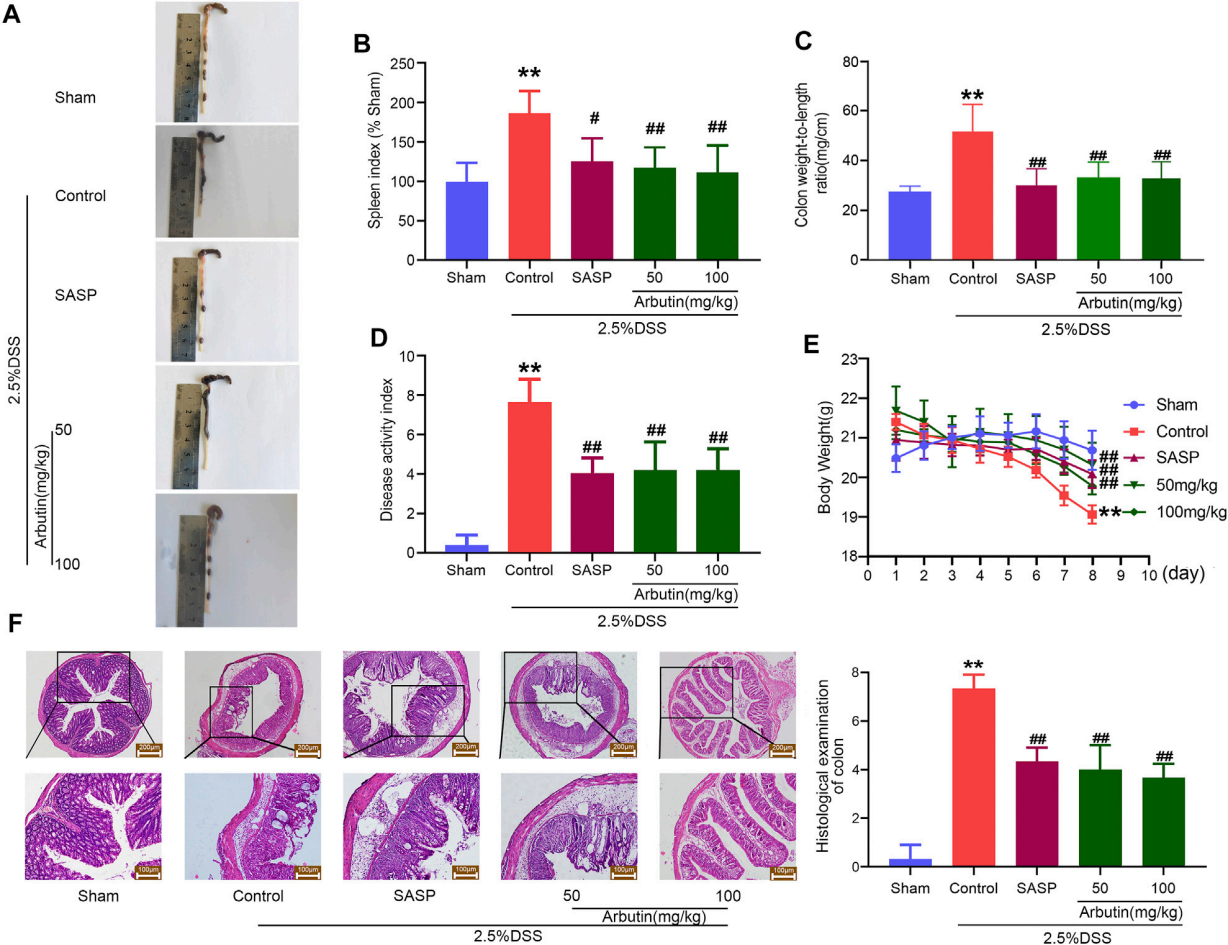
Arbutin alleviates DSS-induced colitis in mice **(A)** macroscopic observation, **(B)** spleen index, **(C)** colon weight-to-length ratio, **(D)** DAI, and **(E)** weight loss **(F)** Representative hematoxylin-eosin-stained colon sections (left panel) and histological score (right panel). Scale: upper panel is 200 μm, lower panel is 100 μm. Data are expressed as the mean ± SD. **p < 0.01 compared with sham operation group; #*p* < 0.05 compared with DSS induced colitis group; ##*p* < 0.01 compared with DSS induced colitis group; *n* = 6 samples.

Colitis symptoms were observed in the DSS-induced colitis mouse model. These included shortened colon length, an increased spleen index, increased colon weight/length ratio, increased DAI, and weight loss (Figures 2A–E). The comparison between the sham and colitis groups revealed that the colitis model was successfully established. Compared with the control mice, the colitis symptoms were partially or completely reversed in the mice treated with arbutin (50 and 100 mg/kg) and SASP. In addition, histological analysis of the colon tissue showed that compared to the sham group, the colonic structure of the mice in the colitis group was destroyed. Ulceration, crypt dilation, goblet cell failure, destruction of the epithelium, and strong infiltration of inflammatory cells were observed in colitis mice (Figure 2F). In contrast, mice treated with arbutin (50 and 100 mg/kg) exhibited complete colonic histological features in the colon tissue. These results suggested that arbutin notably ameliorated the symptoms of colitis.

### Effects of Arbutin on the Inflammatory Responses in Colitis

**FIGURE 3:**
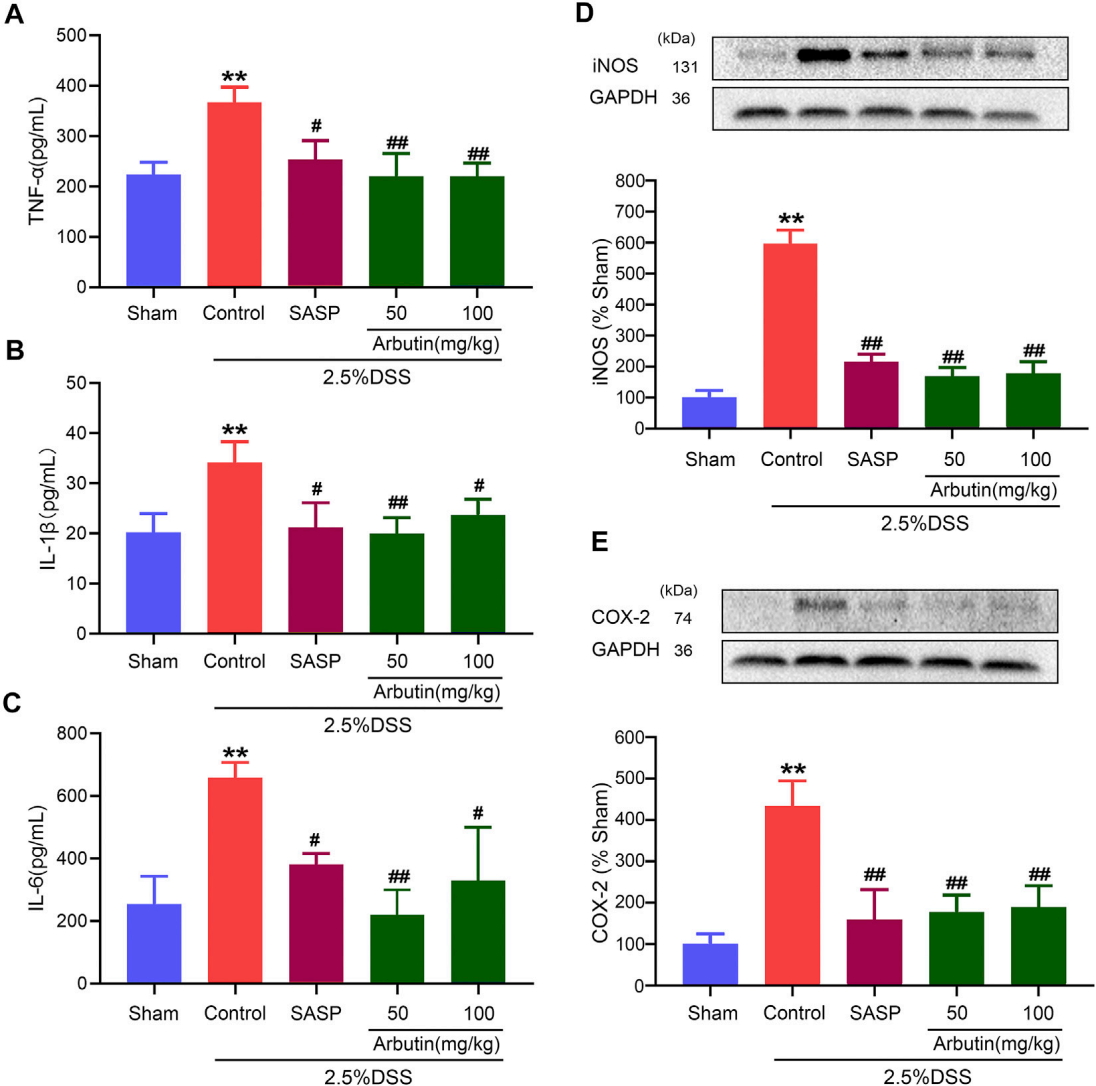
Effects of arbutin on inflammation in colitis. As described in the *Materials and Methods*, blood from mice was centrifugated and were assayed for the determination of the levels of TNF-α **(A)**, IL-1β **(B),** and IL-6 **(C)**, and colon segments from mice were harvested and were used to determine the expression levels of iNOS **(D)** and COX2 **(E)**. Data were expressed as mean ± SD. **p < 0.01 compared with sham operation group; #*p* < 0.05 compared with DSS induced colitis group; ##*p* < 0.01 compared with DSS induced colitis group; *n* = 3 samples for Western blot experiments; *n* = 6 samples for other experiments.

As shown in Figure 3, compared with the mice in the sham group, an ELISA revealed that pro-inflammatory cytokine levels, including those of TNF-α, IL-1β, and IL-6, increased significantly in the murine model of colitis (Figures 3A–C). Western blot analysis showed that levels of inflammation-related proteins (iNOS and COX2) increased after DSS treatment (Figures 3D,E). The increase in cytokines and inflammation-related proteins in colitis were significantly inhibited by gavage administration of 50 and 100 mg/kg arbutin.

### Effects of Arbutin on Intestinal Barrier Dysfunction in Colitis

**FIGURE 4:**
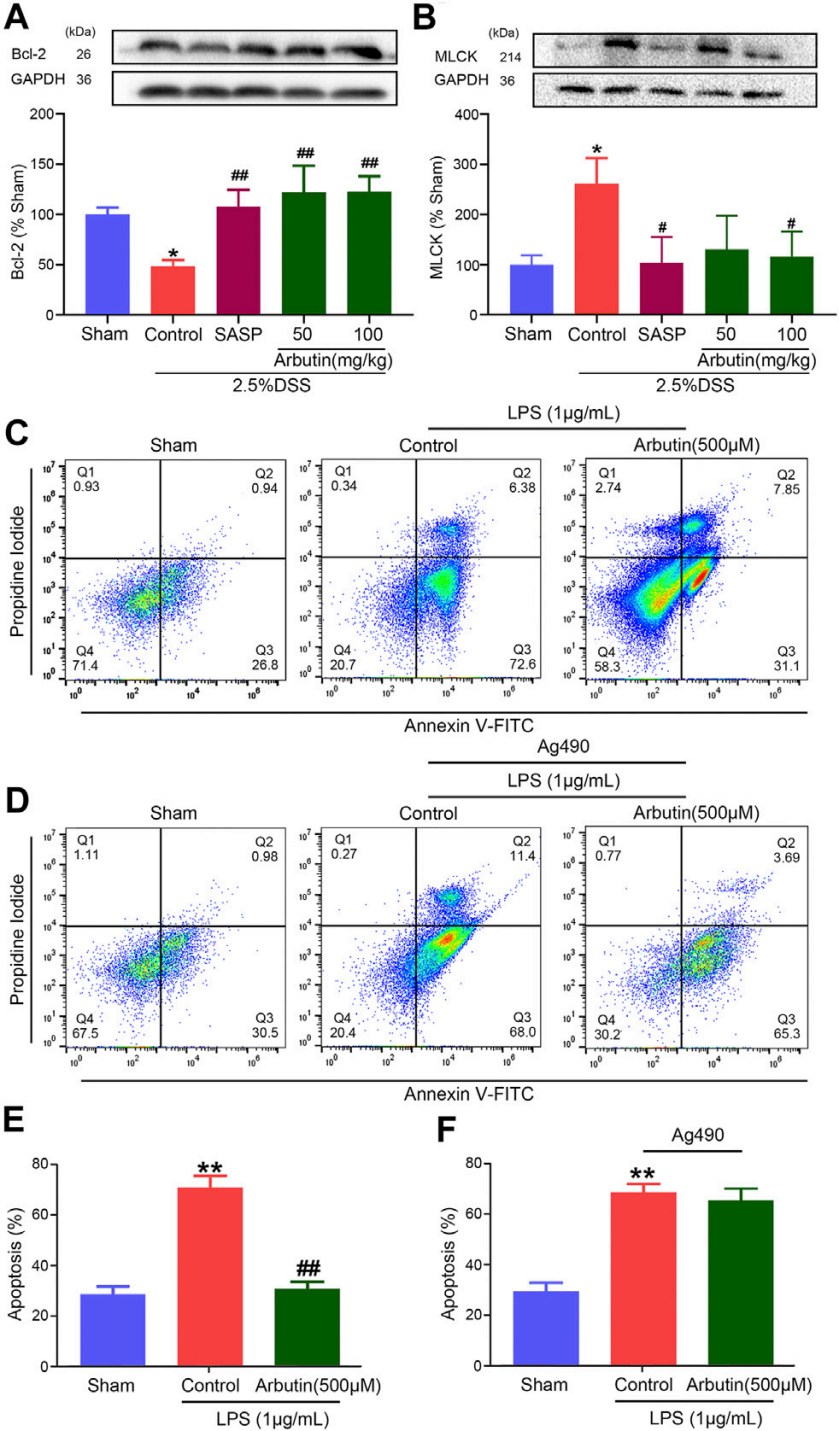
Effects of arbutin on intestinal barrier dysfunction in colitis. Colon segments from mice were harvested and the expression of anti-apoptotic marker bcl-2 **(A)** and tight junction barrier dysfunction marker MLCK **(B)** were analyzed by Western blot analysis **(C**, **E)** IEC-6 cells were given arbutin (500 μM) for 12 h after treatment with LPS (1 μg/ml) for 12 h and the cell apoptosis was determined by flow cytometry **(D**, **F)** IEC-6 cells were pretreated with AG490 (50 μM) for 1 h, treated with LPS (1 μg/ml) for 12 h, and given arbutin (500 μM) for 12 h. Then the cell apoptosis was detected by flow cytometry. Data were expressed as mean ± SD. **p < 0.01 compared with sham group; #*p* < 0.05 compared with control group; ##*p* < 0.01 compared with control group. *n* = 3 samples for Western blot experiments.

Compared with the sham group mice, the colitis model mice displayed a lower level of the colonic anti-apoptotic marker Bcl2 (Figure 4A). The low expression of Bcl2 in colitis was significantly increased by arbutin (Figure 4A). Moreover, the level of the colonic tight junction barrier dysfunction marker MLCK was considerably increased in the colitis model (Figure 4B), which was also reversed by arbutin. These results implied that intestinal epithelial barrier dysfunction in colitis mice was alleviated by arbutin.

The anti-apoptotic effect of arbutin was analyzed in IEC-6 cells using flow cytometry. Cell apoptosis was significantly increased in IEC-6 cells incubated with 1 μg/ml LPS, which was alleviated by 500 μM arbutin. (Figures 4C,E). Arbutin induced inhibition of cell apoptosis was blocked by JAK2 inhibitor AG490 (Figures 4D,F). These outcomes indicated that arbutin could inhibit the apoptosis to maintain intestinal barrier function in colitis, and JAK2 maybe a potential target.

### Effect of Arbutin on JAK2 in Colitis

**FIGURE 5:**
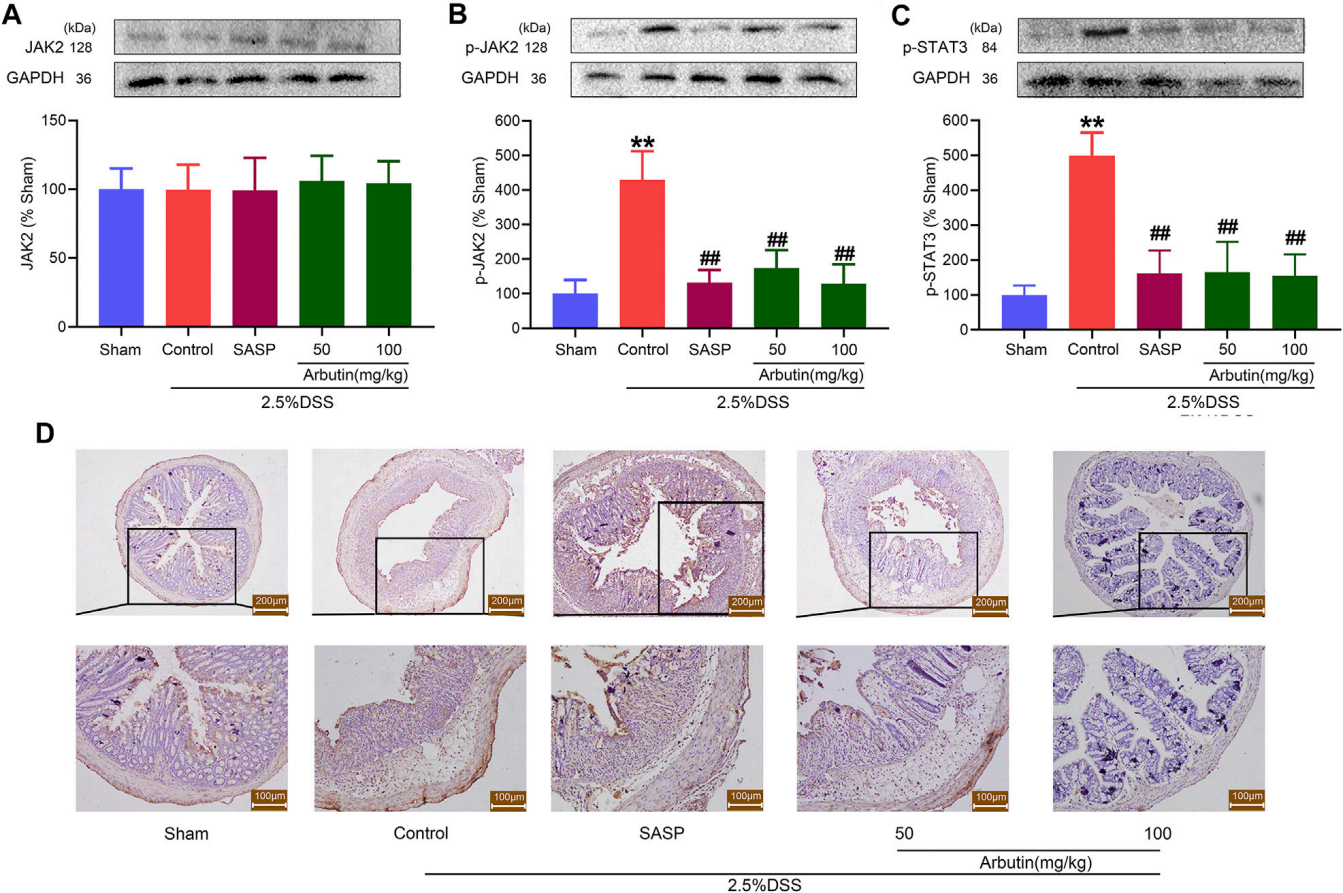
Arbutin inhibited JAK2 signaling pathway in colon. The proteins associated with the JAK2 pathway were detected in mice. Western blot analysis of **(A)** JAK2, **(B)** p-JAK2, and **(C)** p-STAT3 **(D)** Expression of SOCS3 determined by Immunohistochemical staining in the colonic epithelium. Scale bars, 200 (upper panel) and 100 μm (lower panel). Data were expressed as mean ± SD. **p < 0.01 compared with sham operation group; ##*p* < 0.01 compared with DSS induced colitis group; *n* = 3 samples for Western blot experiments; *n* = 6 samples for other experiments.

To investigate the effect of regulating JAK2 by arbutin in a murine model of colitis, the expression of p-JAK2, p-STAT3, and SOCS3 (downstream protein of JAK2) was studied. Our results showed that arbutin did not significantly influence JAK2 expression (Figure 5A). Compared with the sham group, the levels of p-JAK2, p-STAT3, and SOCS3 significantly increased in the colitis group (Figures 5B–D). Moreover, gastric administration of arbutin considerably reversed the expression changes of p-JAK2, p-STAT3, and SOCS3 in colitis. These results suggested the inhibitory effects of arbutin on JAK2 activity in colitis.

### Effect of Arbutin on JAK2 *in vitro*

**FIGURE 6:**
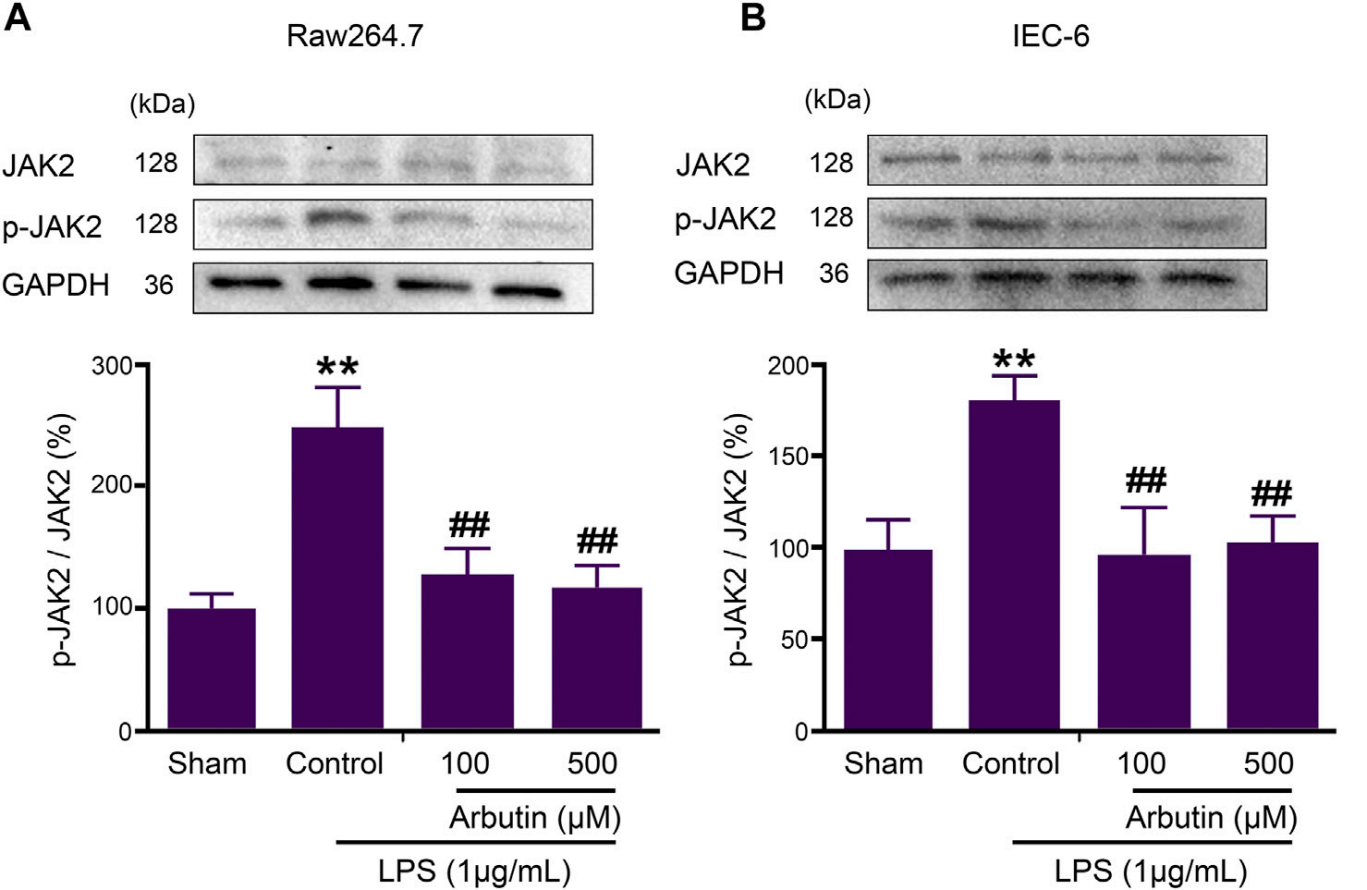
Arbutin inhibited JAK2 signaling pathway in LPS-stimulated IEC6 and RAW264.7 cells. The proteins associated with the JAK2 pathway were determined in cell lines. RAW 264.7 cells **(A)** and IEC-6 cells **(B)** were treated with arbutin (100 and 500 μM) for 12 h after treatment with LPS (1 μg/ml) for 12 h, and the expression levels of JAK2, and p-JAK2 were investigated by Western blot analysis. Data were expressed as mean ± SD. **p < 0.01 compared with sham group; ##*p* < 0.01 compared with LPS control group.

Subsequently, the inhibition of JAK2 activity by arbutin was studied in IEC-6 and raw264.7 cells. The expression of p-JAK2 in IEC-6 and RAW264.7 cells was significantly increased after LPS stimulation, while arbutin reversed this change (Figures 6A,B).

### JAK2 Inhibitor AG490 Blocked Arbutin Induced Therapeutic Effects of Colitis

**FIGURE 7:**
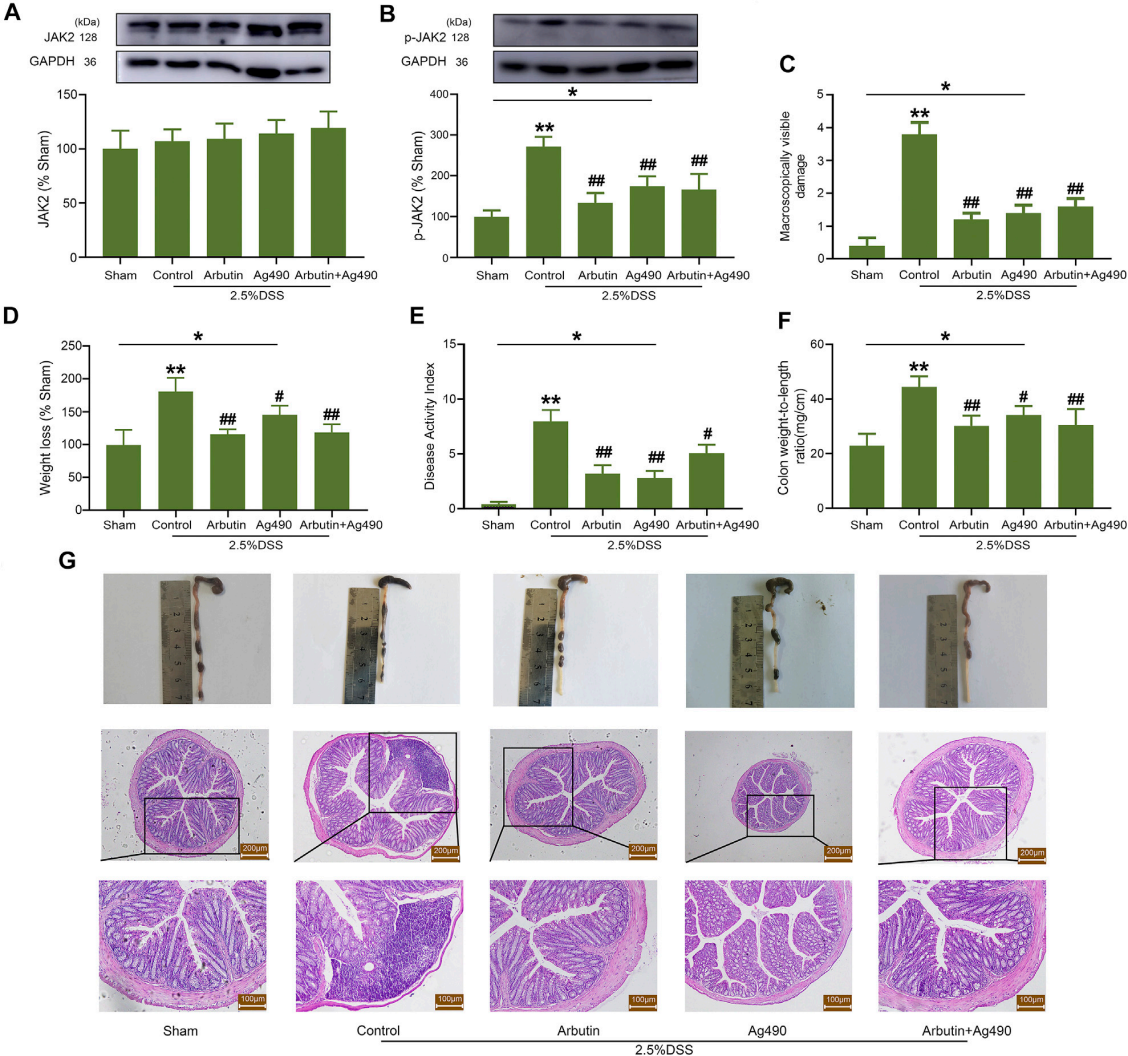
Effects of arbutin and AG490 on UC in mice. The JAK2 protein was silenced by AG490 in mice. Under the condition that AG490 silenced the JAK2, western blot analysis of JAK2 **(A)** and P-JAK2 **(B)**, colonic macroscopically visible damage **(C)**, weight loss **(D)**, DAI **(E)**, colon weight-to-length ratio **(F)**, macroscopic observation and hematoxylin-eosin-stained colon sections **(G)** were recorded. Scale: upper panel is 200 μm, lower panel is 100 μm. Data were expressed as mean ± SD. *p < 0.05 compared between the indicated groups; **p < 0.01 compared with sham group; #*p* < 0.05 compared with DSS control group; ##*p* < 0.01 compared with DSS control group; *n* = 3 samples for Western blot experiments; *n* = 6 samples for other experiments.

AG490, a type of JAK2 inhibitor, was also used to elucidate the role of JAK2 in the treatment of colitis. The expression of JAK2 in colitis was not significantly influenced by arbutin and AG490 (Figure 7A), while the expression of p-JAK2 was significantly suppressed by arbutin and AG490 (Figure 7B). The colitis symptoms, including macroscopically visible damage, weight loss, increase in DAI, colon weight/length ratio, and disruption of the intestinal structure were reversed by the treatment with arbutin and AG490 (Figures 7C–G). Nevertheless, no additional treatment effects were observed upon administration of arbutin in the presence of AG490. These results suggested that the inhibition of JAK2 might be involved in arbutin-induced colitis treatment effects.

**FIGURE 8:**
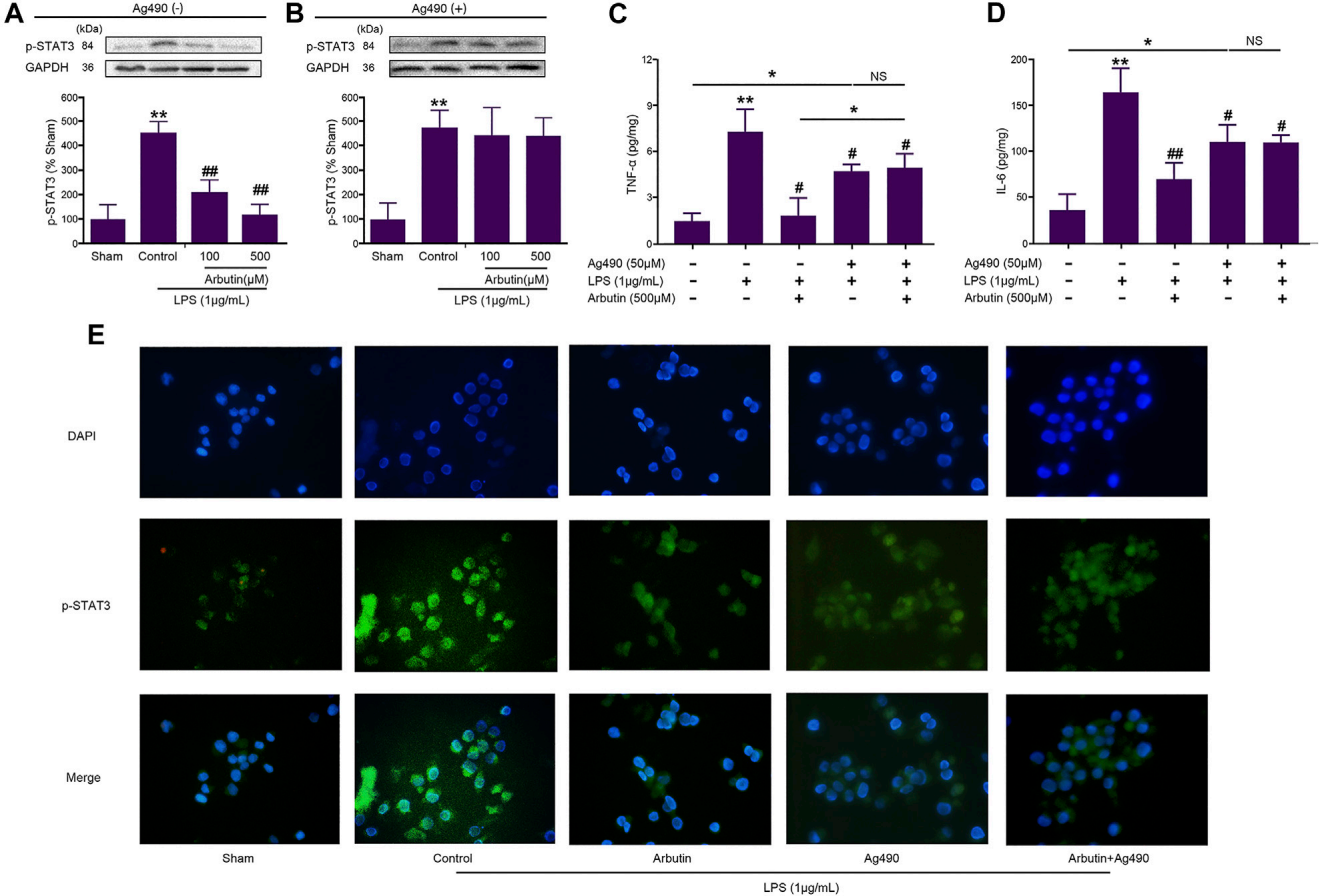
Effects of arbutin and Ag490 on LPS-stimulated IEC6 and RAW 264.7 cells **(A)** IEC-6 cells were given arbutin (100 and 500 μM) for 12 h after treatment with LPS (1 μg/ml) for 12 h and the level of p-STAT3 was determined by western blot analysis **(B)** IEC-6 cells were pretreated with AG490 (50 μM) for 1 h, treated with LPS (1 μg/ml) for 12 h, and given arbutin (100 and 500 μM) for 12 h. Then the level of p-STAT3 was detected by western blot analysis **(C**, **D)** IEC-6 cells were pretreated with AG490 (50 μM) for 1 h, LPS (1 μg/ml) for 12 h, and then treated with arbutin (500 μM) for 12 h, respectively. The TNF-α and IL-6 levels were detected by ELISA **(E)** RAW 264.7 cells were pretreated with AG490 (50 μM) for 1 h, treated with LPS (1 μg/ml) for 12 h, and given arbutin (500 μM) for 12 h, and the p-STAT3 expression was detected by immunofluorescence as described in the *Materials and Methods*. Data were expressed as mean ± SD. *p < 0.05 compared between the indicated groups; **p < 0.01 Compared with sham group; #*p* < 0.05 compared with LPS control group; ##*p* < 0.01 compared with LPS control group; NS *p* > 0.05 compared between the indicated groups.

As shown in Figure 8, the levels of p-STAT3 (a downstream protein of JAK2), TNF-α, and IL-6, were significantly increased by LPS in IEC-6 (Figures 8A,C,D) and RAW264.7 (Figure 8E) cells, which was significantly inhibited by arbutin. The cells were pretreated by AG490, and then incubated by LPS and arbutin. Arbutin induced p-STAT3 reduction (downstream of JAK2) was significantly blocked by AG490, which suggested that arbutin maybe a potential JAK2 inhibitor (Figure 8B). Arbutin induced decrease in TNF-α and IL-6 was partially blocked by AG490 (especially TNF-α). These results suggested that multiples targets besides JAK2 be involved in arbutin induced inhibition of cytokines release. Therefore, we assumed that arbutin may be an JAK2 inhibitor and competitively bind to JAK2.

Molecular docking analysis was performed to investigate the biding mechanisms between arbutin and JAK2. The predicted binding mode of arbutin with JAK2 is shown in the Supplementary Figure S1. The oxygen atom of arbutin acted as a hydrogen bond donor to form a hydrogen bonding interaction with the backbone nitrogen atom of residue Leu932. Moreover, the N2 and O3 atoms of the *o*-dihydroxybenzene ring in tyrphostin AG-490 were respectively connected to the main chain residue (Leu855) of the N-terminal leaf of JAK2 and the Leu932 residue of the hinge ring to form a hydrogen bond (Rashid et al., 2015). The formation of hydrogen bonding interactions and similarity of the binding structure to Leu932 further confirmed that arbutin might be a potential binding inhibitor of JAK2.

## Discussion

In this study, arbutin was found to significantly alleviate the symptoms of colitis by inhibiting the activity of JAK2. The main pathological features of a DSS-induced UC mouse model were previously found to include infiltration of inflammatory cells in the lamina propria and submucosa, leading to mucosal erosion, expansion of submucosa, and destruction of the intestinal barrier function (DeRoche et al., 2014). Arbutin considerably inhibited cytokine-induced cascade amplification of inflammatory signals as well as destruction of the barrier function caused by intestinal epithelial apoptosis in the colitis model. Inhibition of JAK2 by AG490 *in vivo* and *in vitro* notably hindered the effect of arbutin on the symptoms of colitis.

In inflammatory bowel disease, due to the infiltration of inherent and adaptive immune cells into the lamina propria, the concentration of local proinflammatory cytokines (IL-1β, IL-6, and TNF-α) increases, which affects the immune balance required for normal intestinal homeostasis (Lee and Kim, 2012; Műzes et al., 2012). In addition, continuous inflammation further damages the integrity of the intestinal barrier, leading to increased apoptosis, destruction of tight junctions among epithelial cells, and eventually to intestinal edema and ulcer formation (Grivennikov et al., 2009). In this study, we determined that arbutin considerably downregulated the levels of inflammatory cytokines (IL-1β, IL-6, and TNF-α) and proteins (iNOS and COX-2) in colitis mice. Furthermore, arbutin could significantly reverse the changes of the anti-apoptotic marker Bcl2 and tight junction barrier dysfunction marker MLCK in colitis mice (Sui et al., 2020). The results obtained herein indicate that arbutin could adjust unbalanced inflammatory signals in the intestinal tract and protect the intestinal barrier during inflammation.

It has been demonstrated that arbutin targets a variety of signal transduction pathways, including Sirt1/Nrf2, TLR-4/NF-κB, and p-AMPK/p62 (Ye et al., 2019; Ding et al., 2020; Nalban et al., 2020), indicating that it can resist apoptosis, inflammation, and oxidative stress. In this work, by molecular docking analysis, we found that arbutin might inhibit the transduction of the JAK2 signaling pathway by binding to JAK2. Our findings provide another potential mechanism, by which arbutin alleviates colitis.

The JAK/STAT3 signaling pathway plays a key role in inflammatory signaling, which can aggravate intestinal inflammatory injury (Salas et al., 2020). In UC patients, the severity of bowel disease is positively correlated with the expression of JAK2 and its downstream STAT3 (Lin et al., 2017). In addition, SOCS3, a negative regulator of the JAK2 signal pathway, is upregulated as result of the aggravation of the JAK2 signal pathway in patients diagnosed with colitis (Liu et al., 2015). In the present study, arbutin significantly inhibited the phosphorylation of JAK2 and its downstream STAT3. Moreover, it downregulated the expression of SOCS3 in a DSS-induced colitis model. To further explore the mechanism of arbutin, we used a specific JAK2 inhibitor, namely AG490, to block the JAK2 signaling pathway. The obtained results indicated that both AG490 and arbutin could alleviate colitis symptoms, downregulate inflammatory indexes, and inhibit the phosphorylation of JAK2 and STAT3 in a mouse colitis model as well as in LPS-induced epithelial and immune cell inflammation models. Nevertheless, no further therapeutic effect of arbutin was observed in the presence of AG490. These outcomes suggested that the therapeutic effect of arbutin might be related to the inhibition of JAK2.

Intestinal epithelium forms a key barrier, which separates the body from the external environment. The balance between proliferation and apoptosis of the intestinal epithelial cells is important to maintain the integrity of the barrier (Negroni et al., 2015). However, colonic epithelial cells in UC patients exhibit a higher rate of apoptosis (Souza et al., 2005). The JAK pathway plays an important role in regulating cell proliferation and apoptosis (Bousoik and Montazeri Aliabadi, 2018). Furthermore, the conducted experiments demonstrated that inhibition of the JAK2 pathway could alleviate cell apoptosis caused by intestinal ischemia-reperfusion (Lian et al., 2017). Arbutin treatment could significantly inhibit LPS-induced apoptosis in intestinal epithelial cells; however, this effect was reversed following pretreatment with AG490. These outcomes implied that arbutin could improve the barrier function by regulating the JAK2 signal transduction to control cell proliferation and apoptosis. However, the anti-apoptotic effect of arbutin was mainly studied *in vitro* and slightly involved *in vivo* by western blot. Further experiments including *in situ* TUNEL staining are needed to verify the anti-apoptotic effect of arbutin *in vivo*.

In conclusion, arbutin treatment appeared to significantly inhibit JAK2 signal transduction; therefore, it was determined that it played an important protective role in DSS-induced colitis mice. The findings of this study provide some understanding of the role of JAK2 inhibitors in IBD and could lead to the application of arbutin as a small molecule drug for the treatment of the disease. In future, we will try to construct JAK2-knock down mice to study the effects of arbutin on mice colitis.

## Data Availability

The original contributions presented in the study are included in the article/Supplementary Material, further inquiries can be directed to the corresponding authors.
