# Supplementary material for: Arbutin Ameliorates Murine Colitis by Inhibiting JAK2 Signaling Pathway
Source: Front Pharmacol. 2021 Sep 14;12:683818. doi: 10.3389/fphar.2021.683818 (PMC8477021; doi:10.3389/fphar.2021.683818)
Supplement: Supplementary file 1 [file DataSheet1.zip › original WB images.docx]

Representative bands Raw data 1 Raw data 2


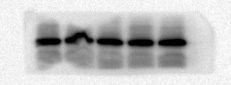


Fig.3


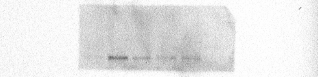

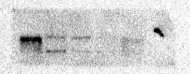

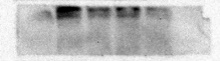


COX-2 1 29 57


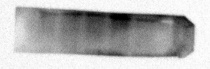

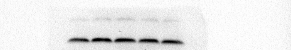


GAPDH 2 30 58


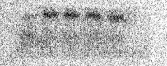


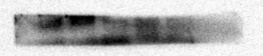

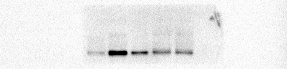
iNOS 3 31 59


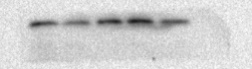

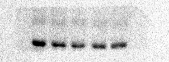

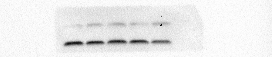


GAPDH 4 32 60

Fig.4


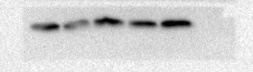

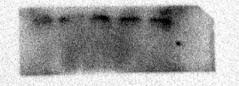


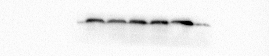
Bcl-2 5 33 61


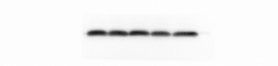


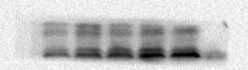

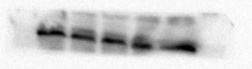
GAPDH 6 34 62


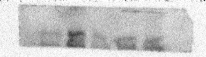

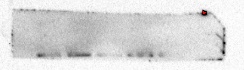

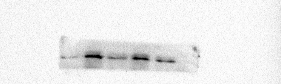


MLCK 7 35 63


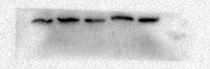

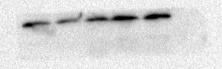


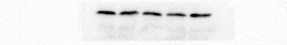
GAPDH 8 36 64


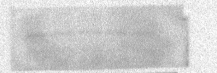

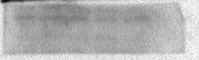

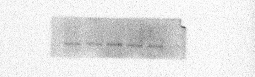
Fig.5

JAK2 9 37 65


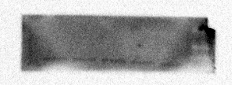

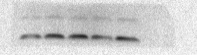

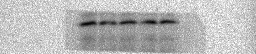


GAPDH 10 38 66


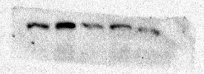

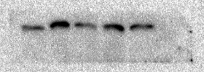

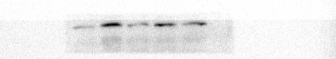


p-JAK2 11 39 67


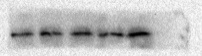

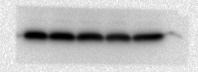

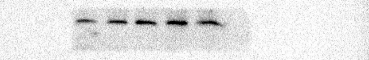


GAPDH 12 40 68


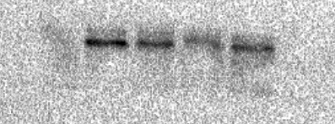

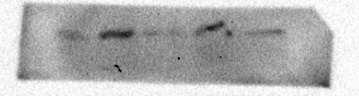

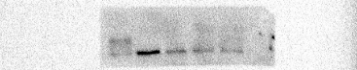


p-STAT3 13 41 69


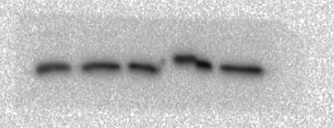

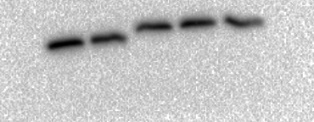

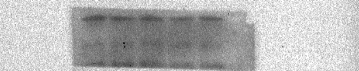


GAPDH 14 42 70

Fig.6

RAW


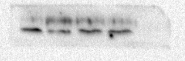

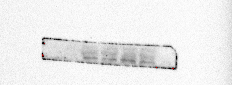

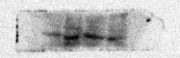
JAK2 15 43 71


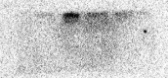

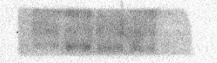


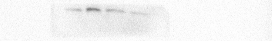
p-JAK2 16 44 72


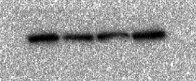


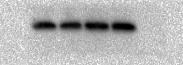

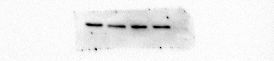
GAPDH 17 45 73


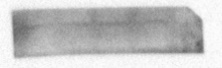

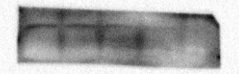

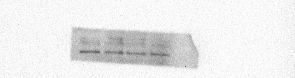
IEC6

JAK2 18 46 74


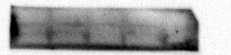

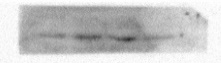

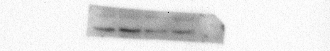


p-JAK2 19 47 75


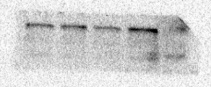

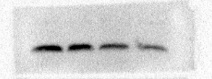


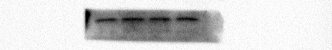
GAPDH 20 48 76

Fig. 7


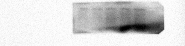

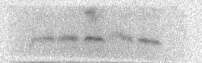


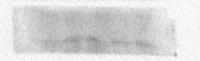
JAK2 21 49 77


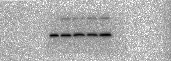


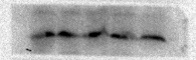

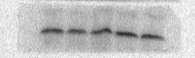
GAPDH 22 50 78


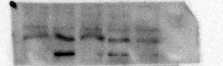

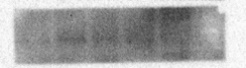


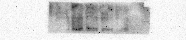
p-JAK2 23 51 79


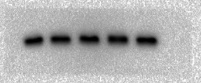

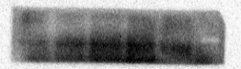

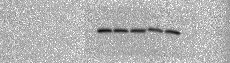


GAPDH 24 52 80


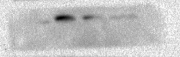

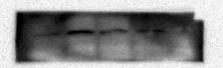

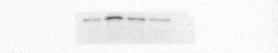
Fig. 8

p-STAT3 25 53 81


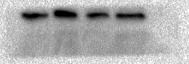

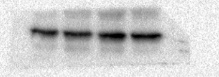


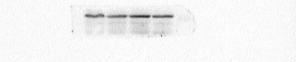
GAPDH 26 54 82


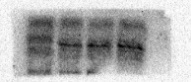

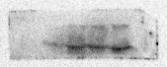

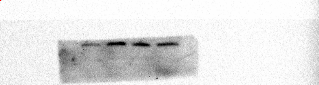
Ag490

p-STAT3 27 55 83


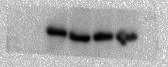

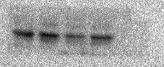

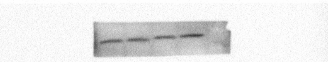


GAPDH 28 56 84
