# Supplementary figures and images for: Arbutin Ameliorates Murine Colitis by Inhibiting JAK2 Signaling Pathway
Source: Front Pharmacol. 2021 Sep 14;12:683818. doi: 10.3389/fphar.2021.683818 (PMC8477021; doi:10.3389/fphar.2021.683818)

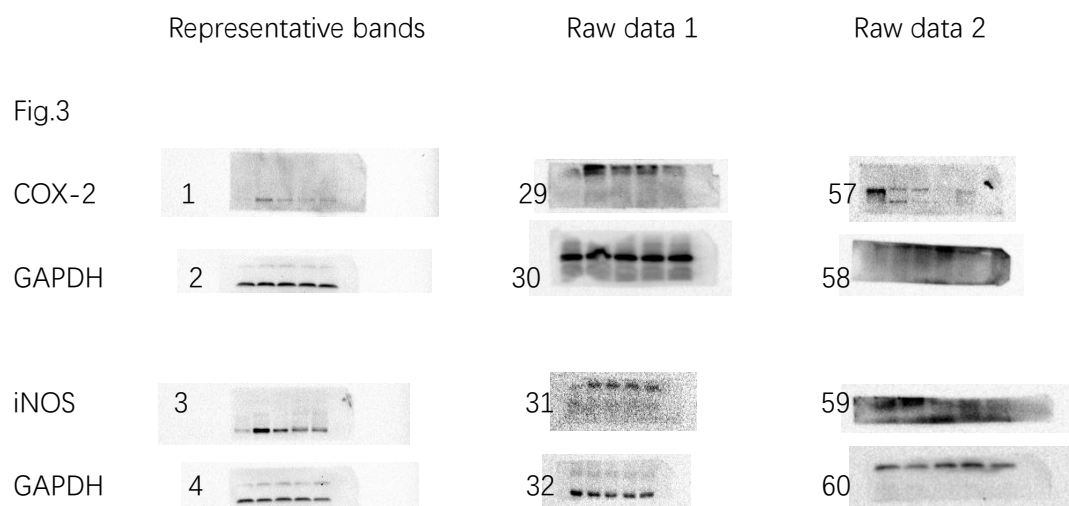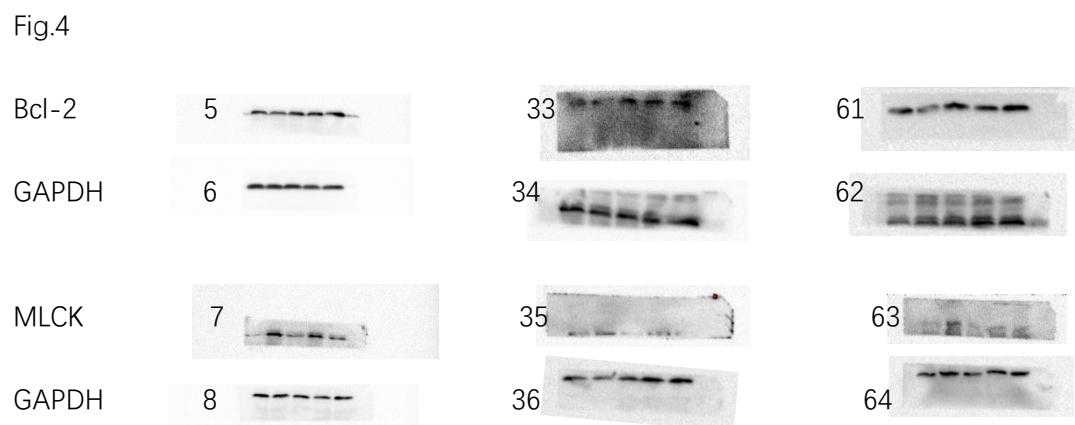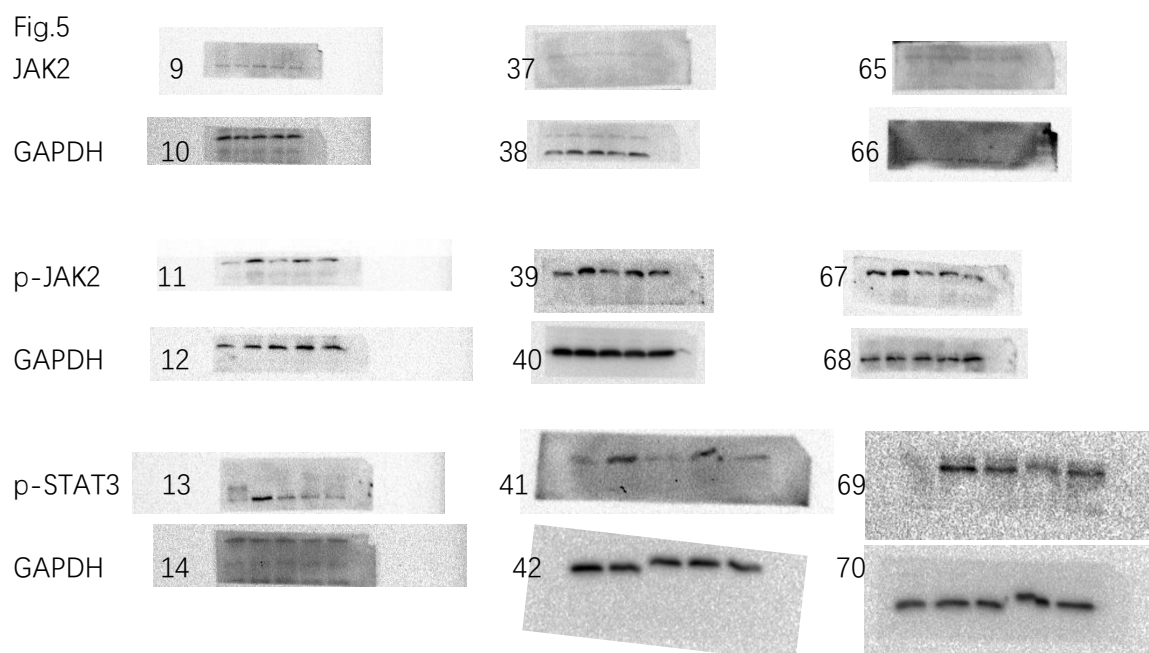

Fig.6

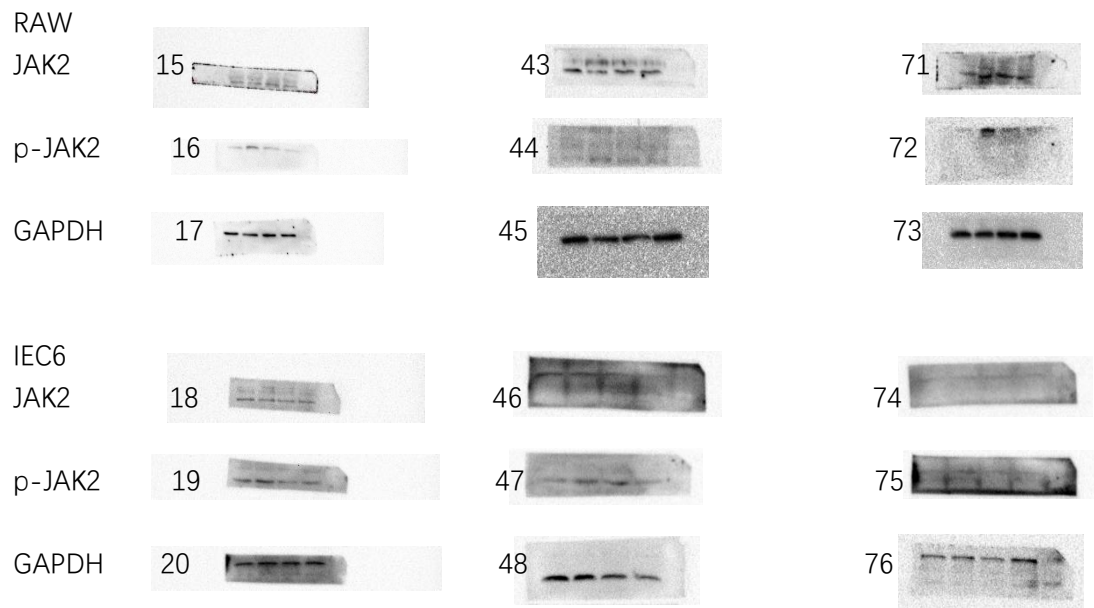

Fig. 7

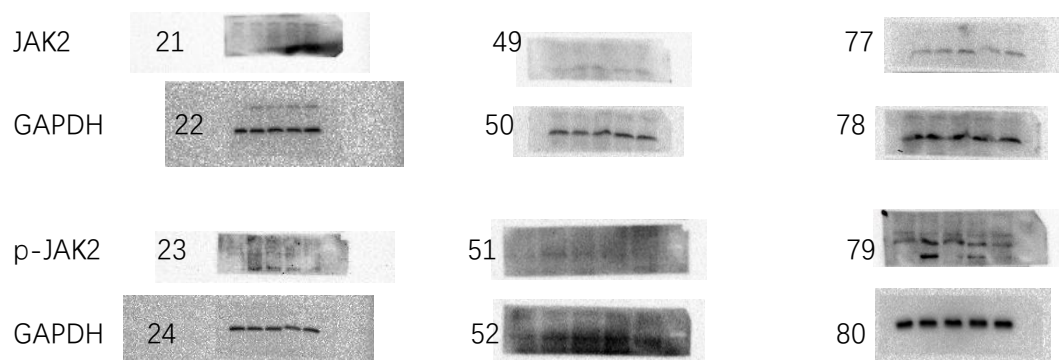

Fig. 8

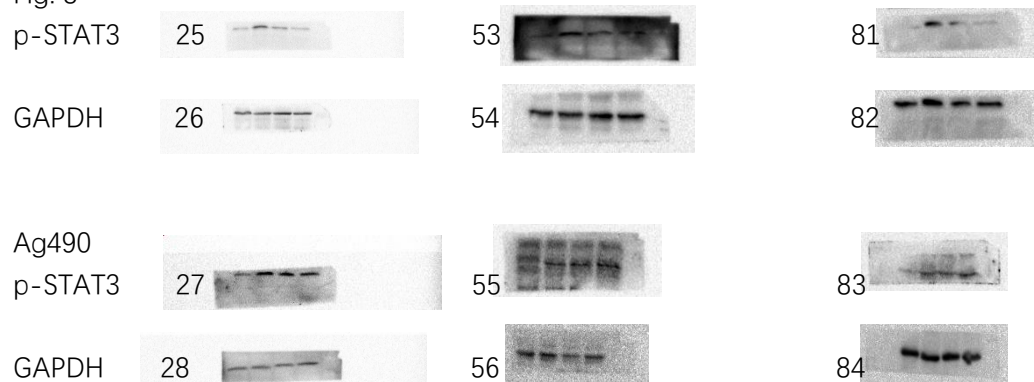

Supplement: Supplementary file 1 [file DataSheet1.zip › original WB images.pdf]

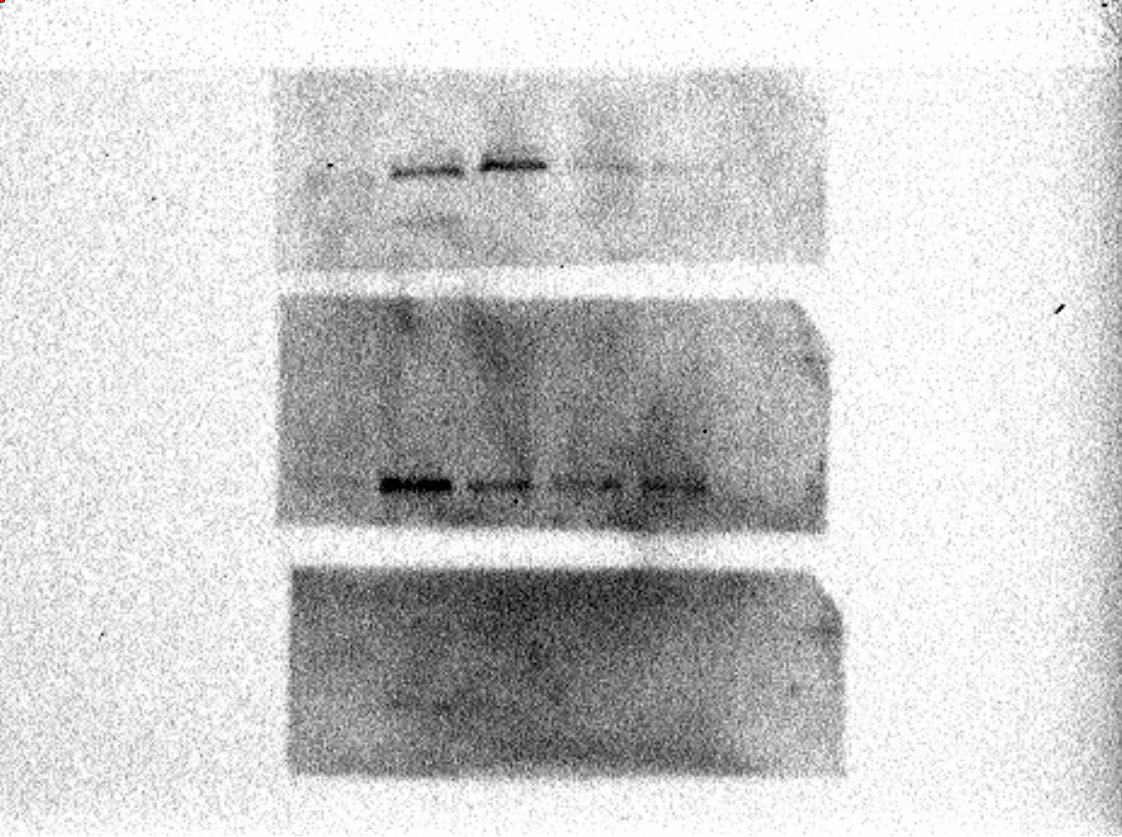

Supplement: Supplementary file 1 [file DataSheet1.zip › original WB images/1(COX2) 3(iNOS).tif]

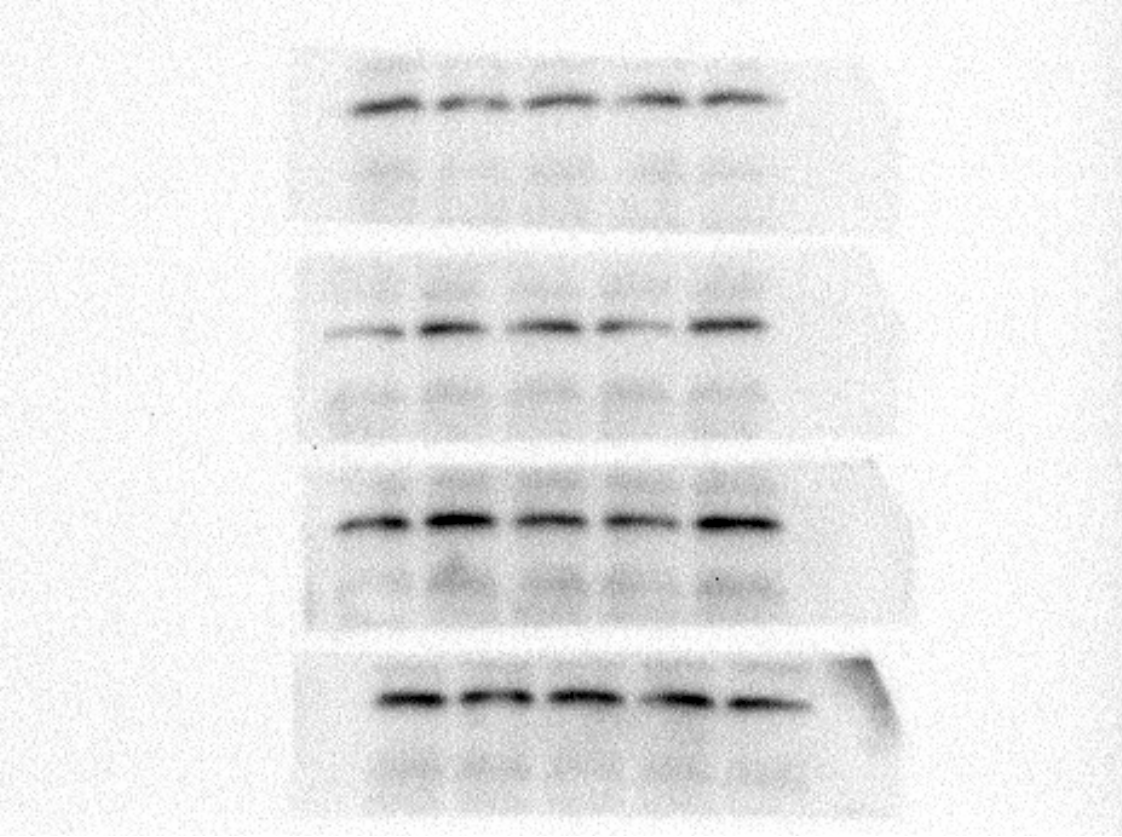

Supplement: Supplementary file 1 [file DataSheet1.zip › original WB images/10(GAPDH-JAK2).tif]

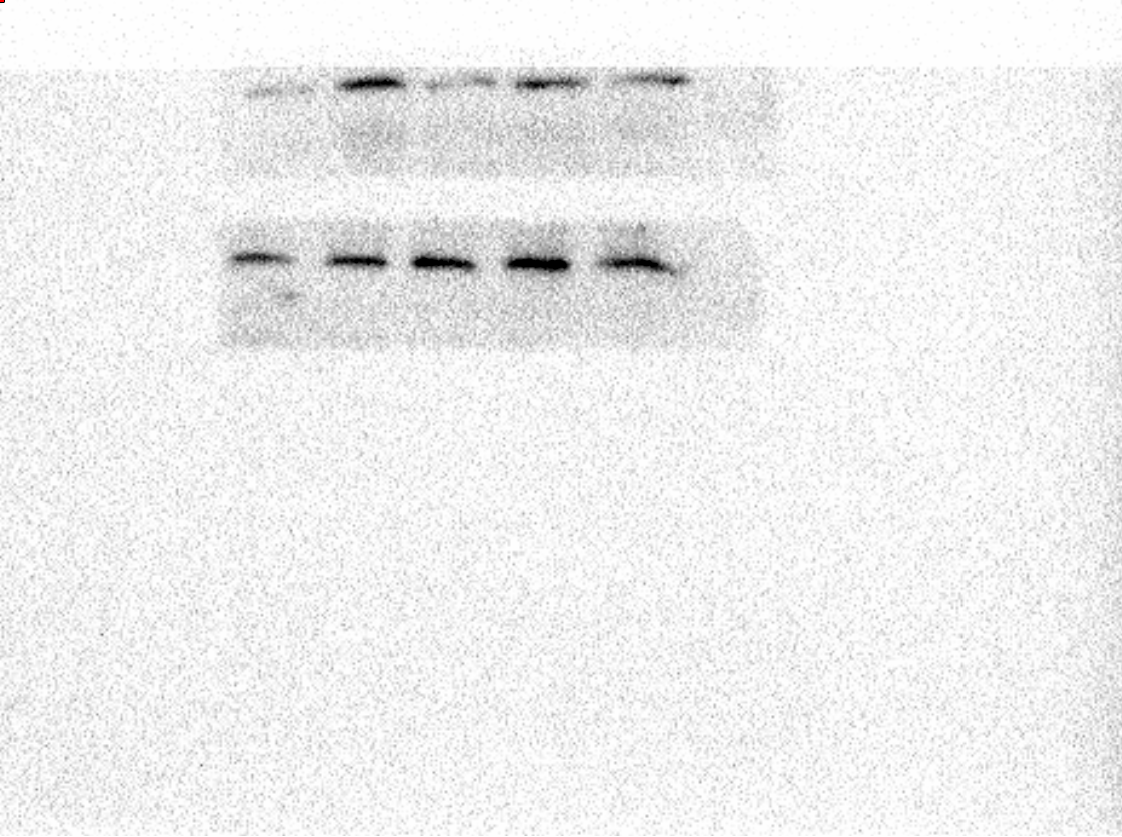

Supplement: Supplementary file 1 [file DataSheet1.zip › original WB images/11(p-JAK2) 12(GAPDH-pJAK2).tif]

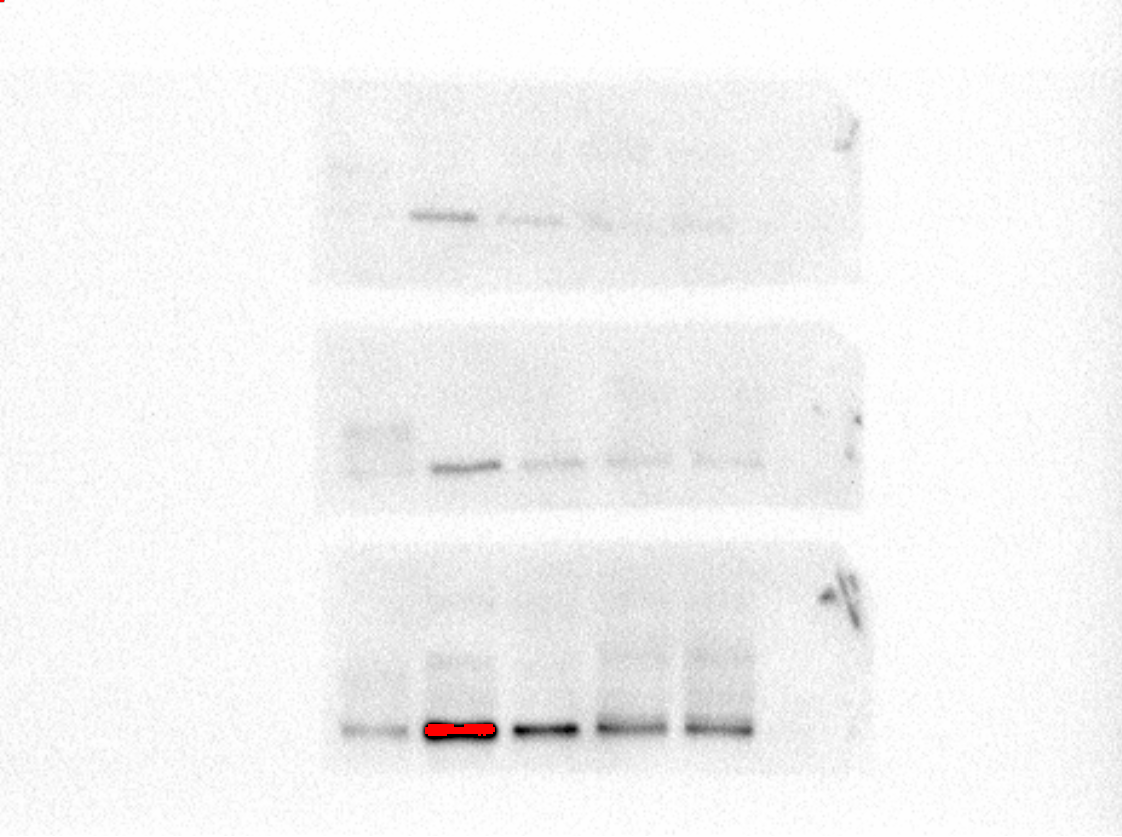

Supplement: Supplementary file 1 [file DataSheet1.zip › original WB images/13(P-STAT3).tif]

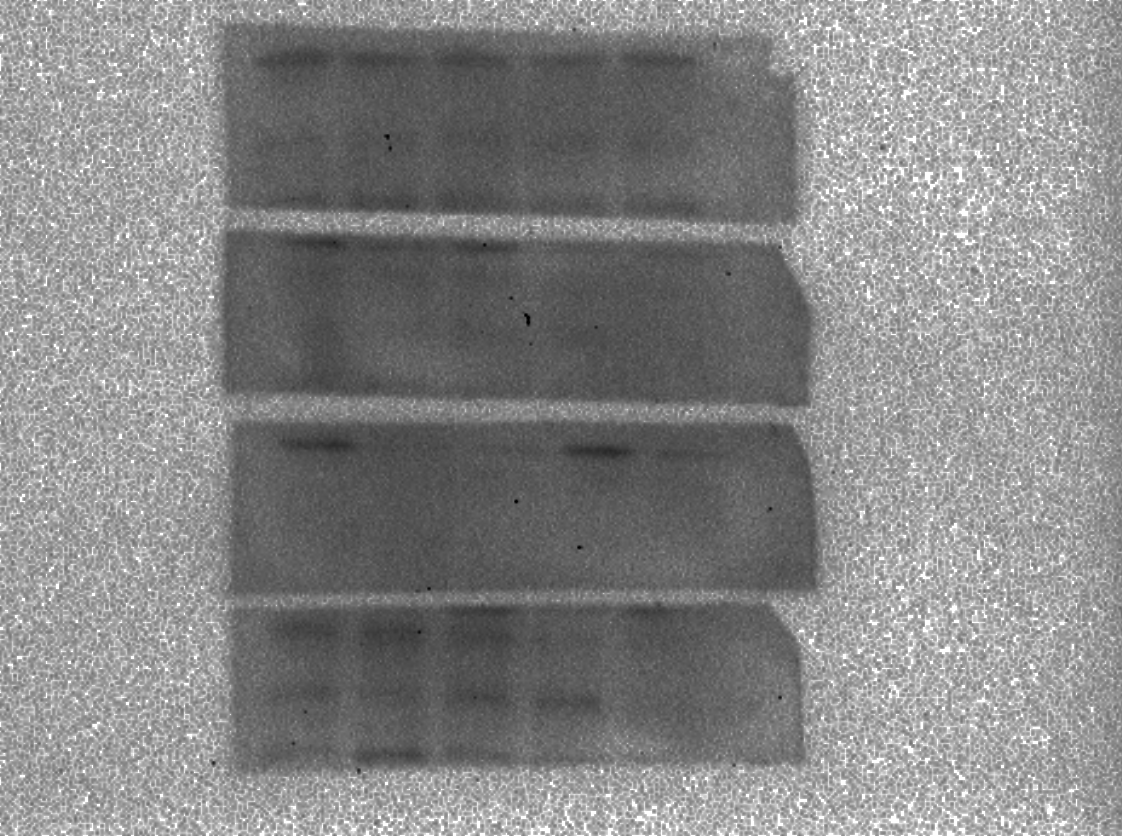

Supplement: Supplementary file 1 [file DataSheet1.zip › original WB images/14(GAPDH-pstat3).tif]

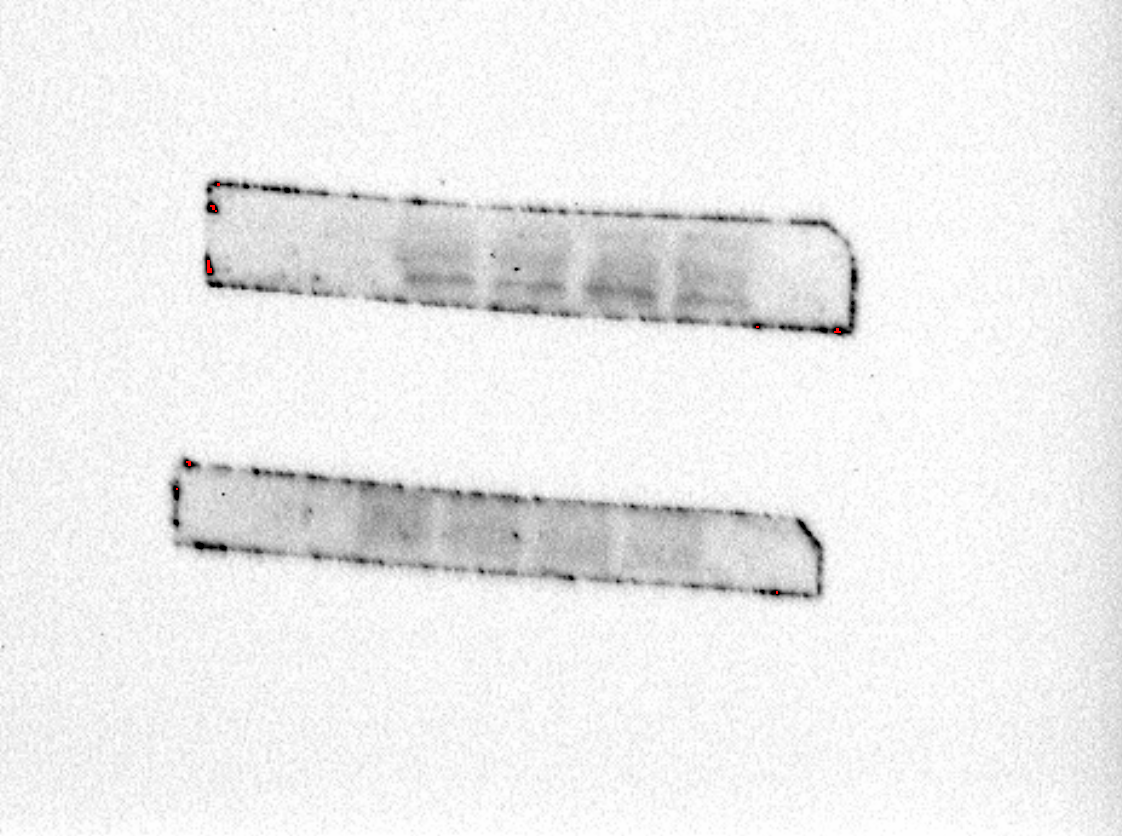

Supplement: Supplementary file 1 [file DataSheet1.zip › original WB images/15(JAK2-RAW).tif]

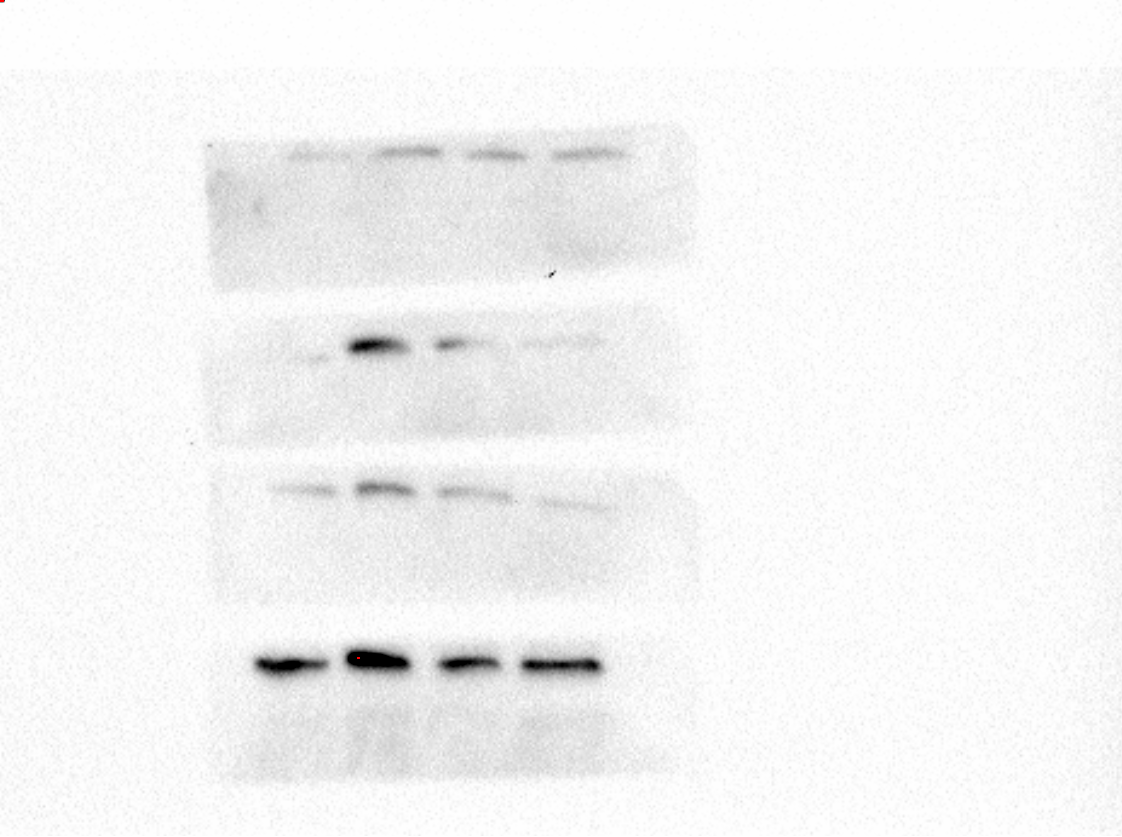

Supplement: Supplementary file 1 [file DataSheet1.zip › original WB images/16(p-jak2 raw) 27(p-stat3 ag490) 81(p-stat3) 82(GAPDH-pstat3).tif]

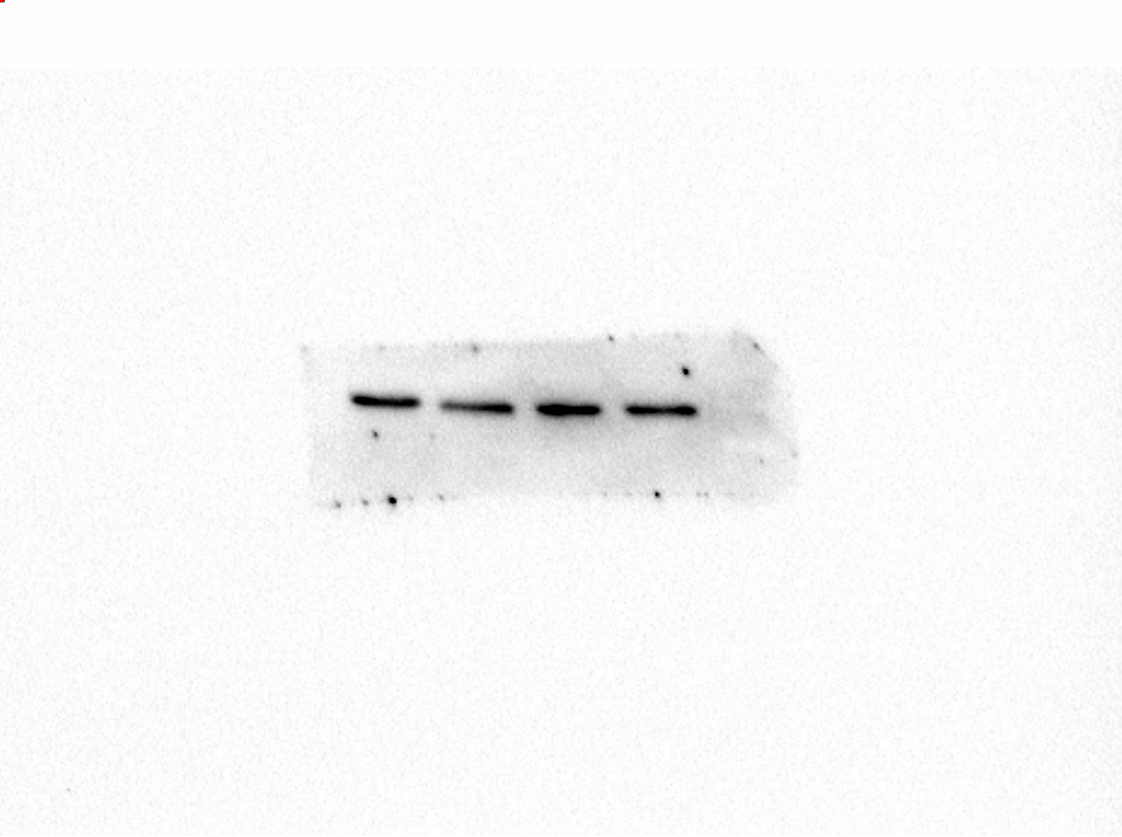

Supplement: Supplementary file 1 [file DataSheet1.zip › original WB images/17(GAPDH-RAW).tif]

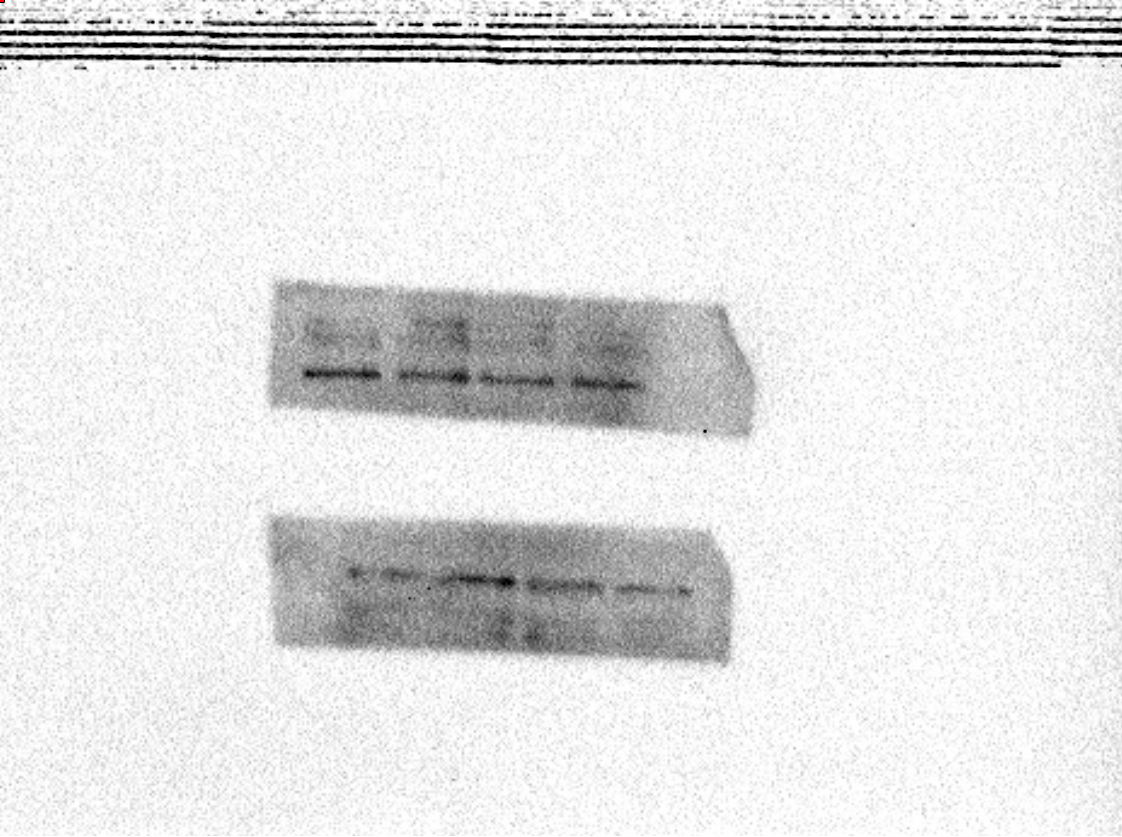

Supplement: Supplementary file 1 [file DataSheet1.zip › original WB images/18(JAK2-IEC6).tif]

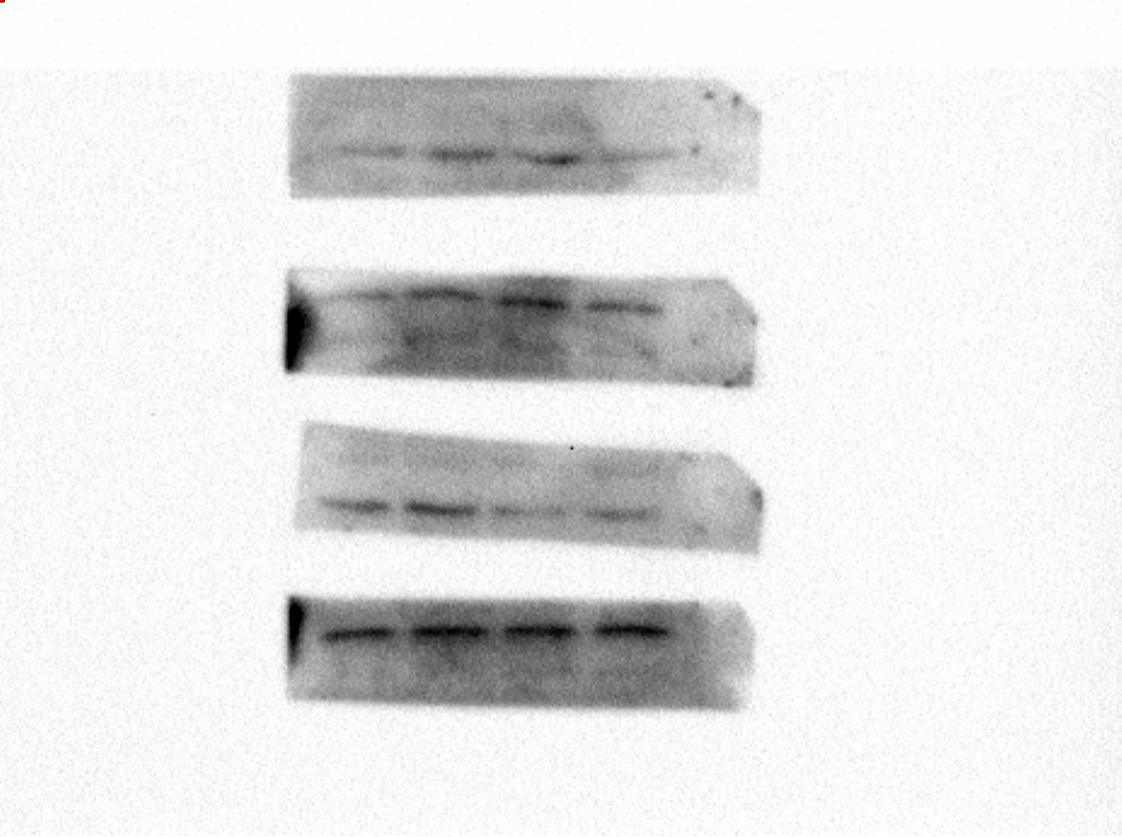

Supplement: Supplementary file 1 [file DataSheet1.zip › original WB images/19(P-JAK2-IEC6) 20(GAPDH-IEC6) 47(p-JAK2-IEC6).tif]

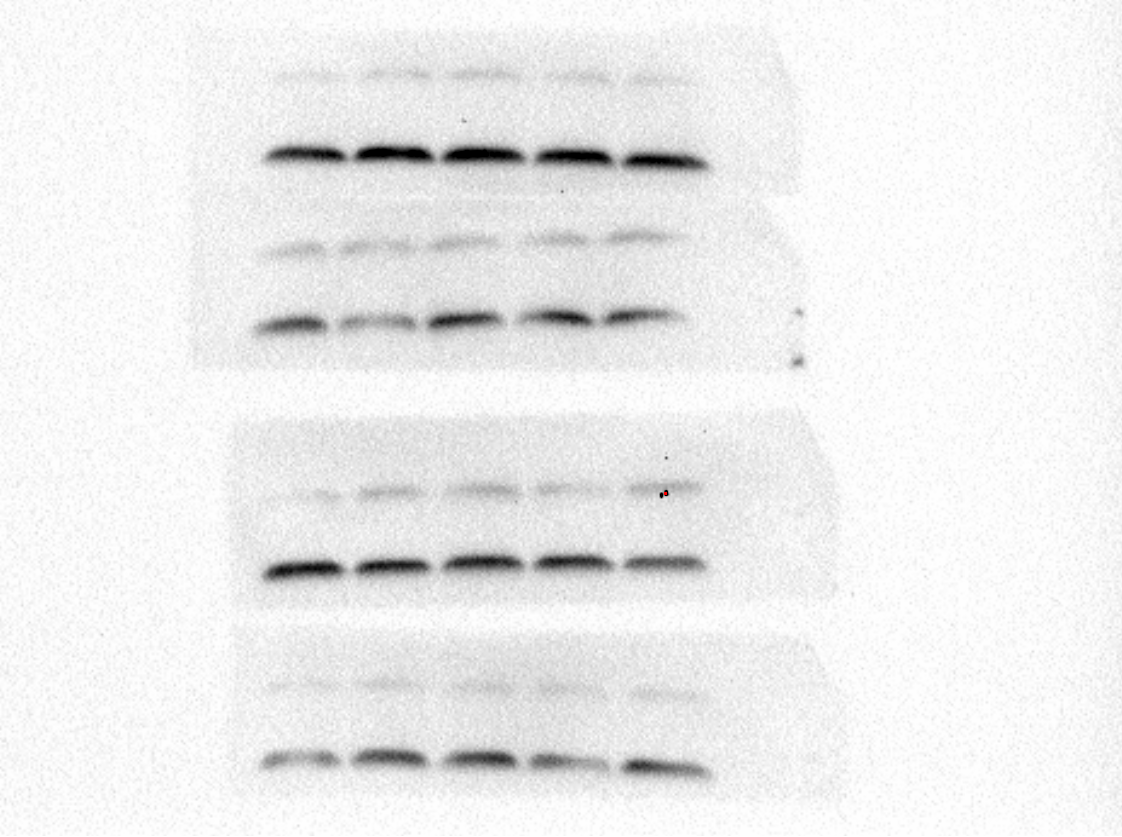

Supplement: Supplementary file 1 [file DataSheet1.zip › original WB images/2(GAPDH-Cox2) 4(GAPDH-inos) 38(GAPDH-JAK2).tif]

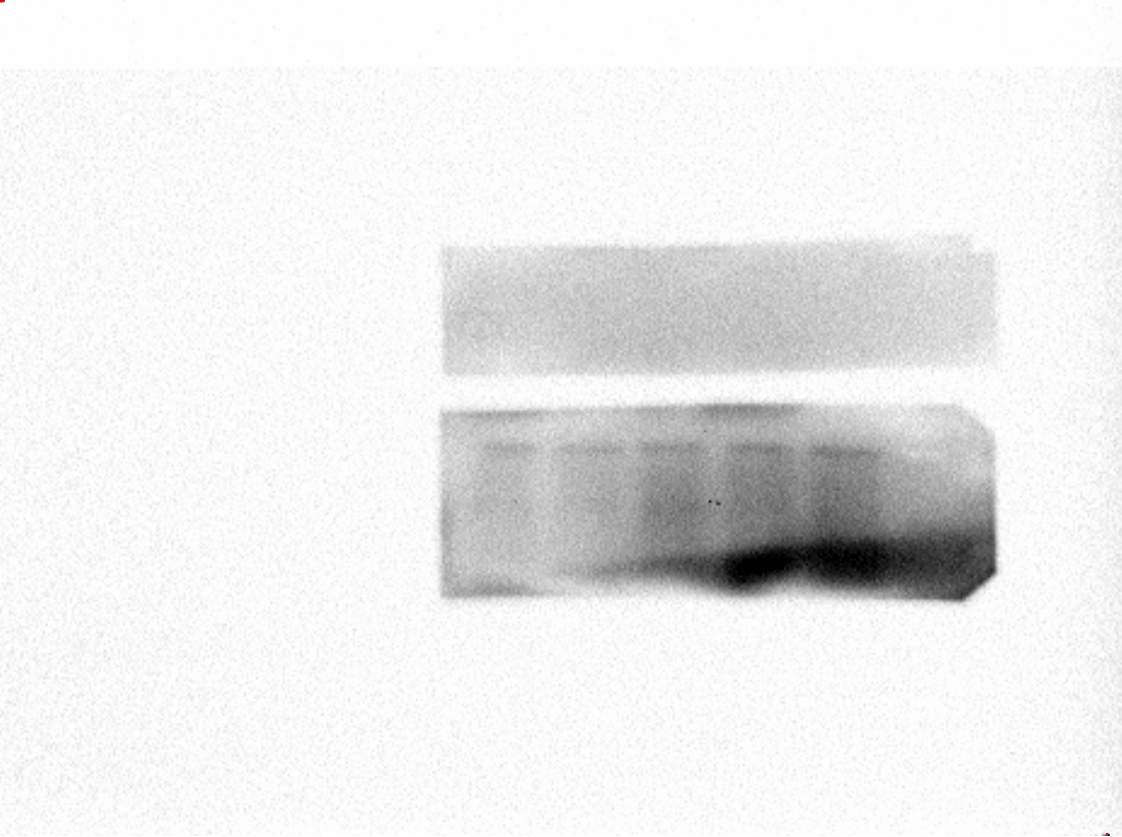

Supplement: Supplementary file 1 [file DataSheet1.zip › original WB images/21(JAK2).tif]

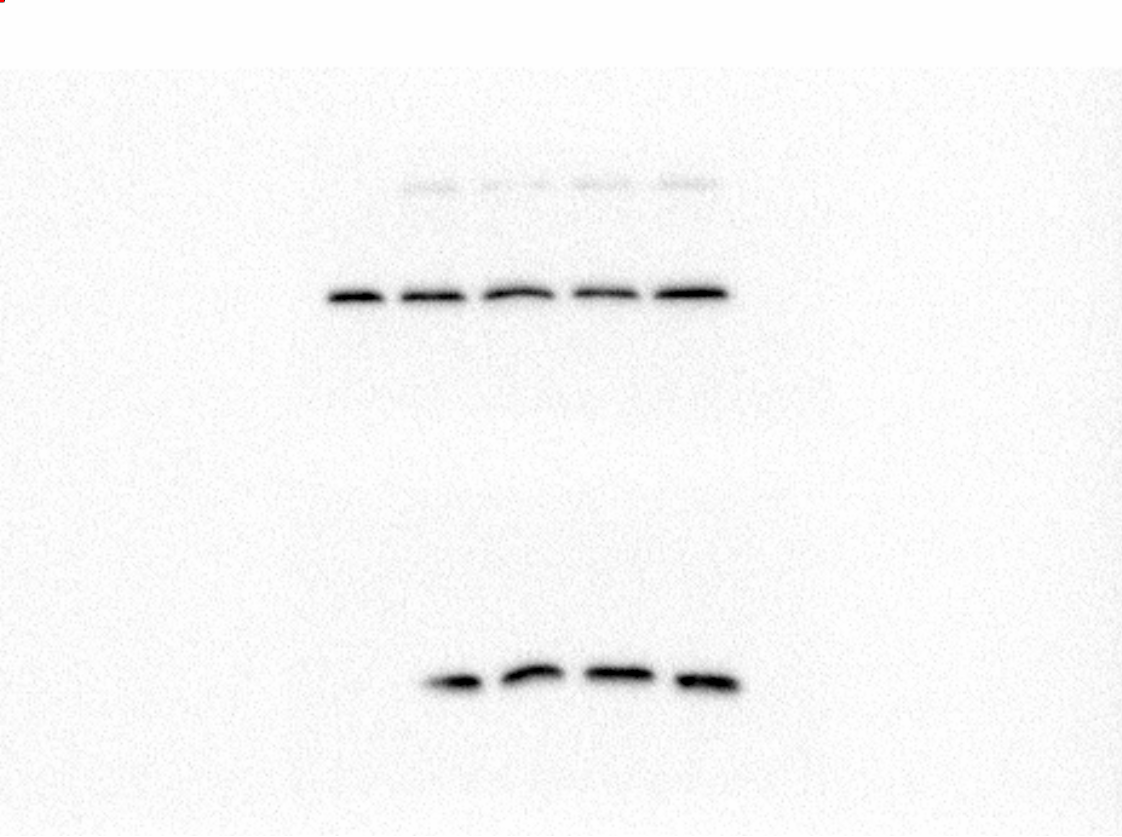

Supplement: Supplementary file 1 [file DataSheet1.zip › original WB images/22(GAPDH-JAK2).tif]

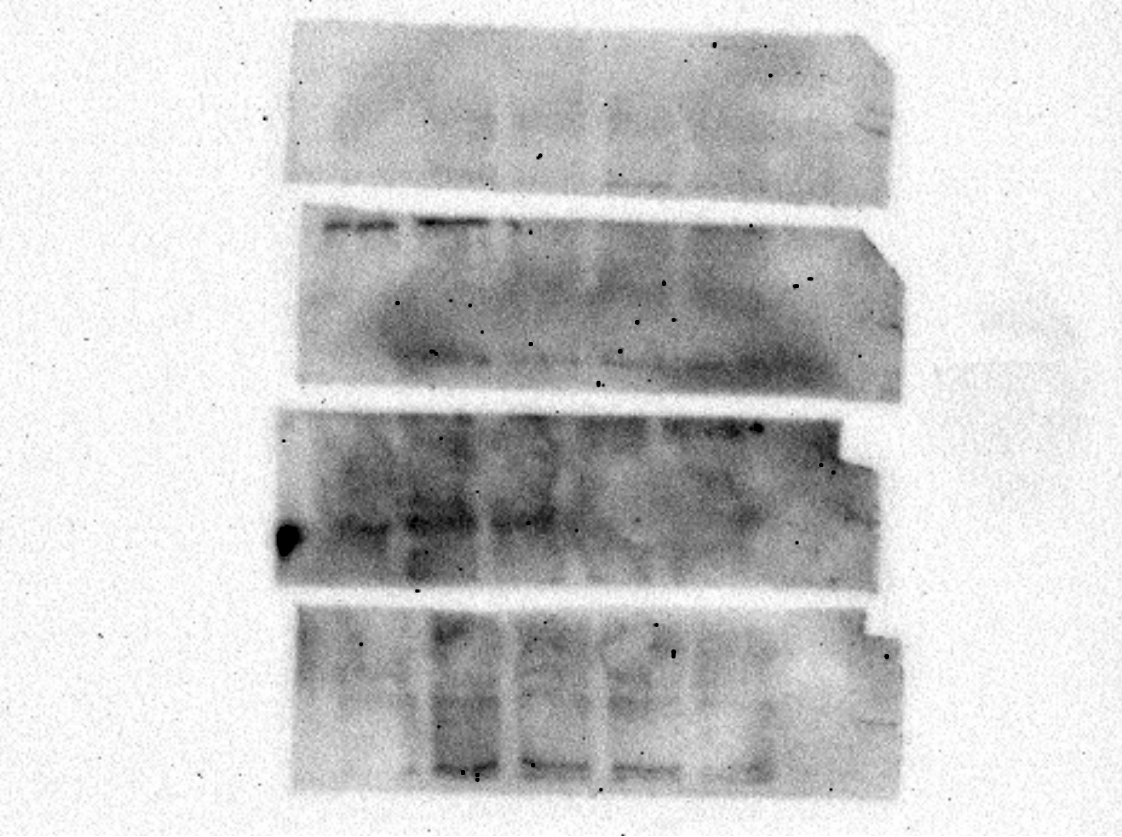

Supplement: Supplementary file 1 [file DataSheet1.zip › original WB images/23(p-JAK2).tif]

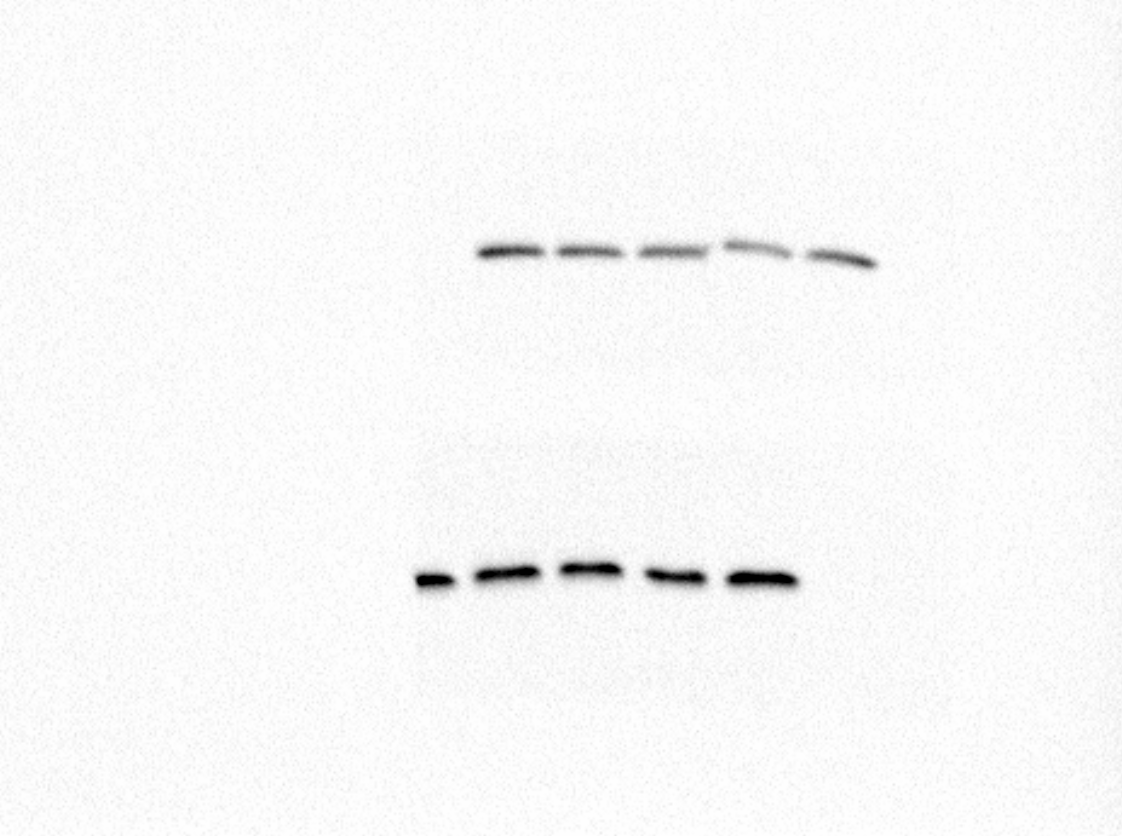

Supplement: Supplementary file 1 [file DataSheet1.zip › original WB images/24(GAPDH-pjak2).tif]

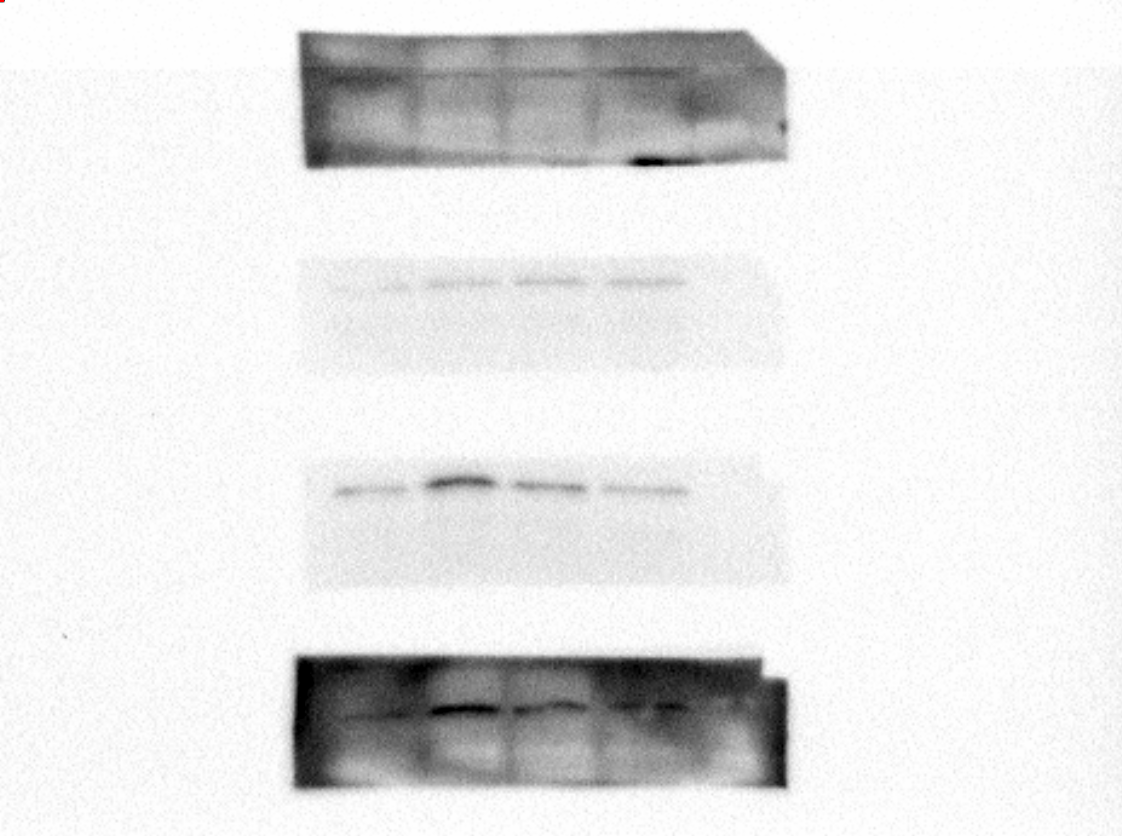

Supplement: Supplementary file 1 [file DataSheet1.zip › original WB images/25(p-STAT3) 53(p-STAT3).tif]

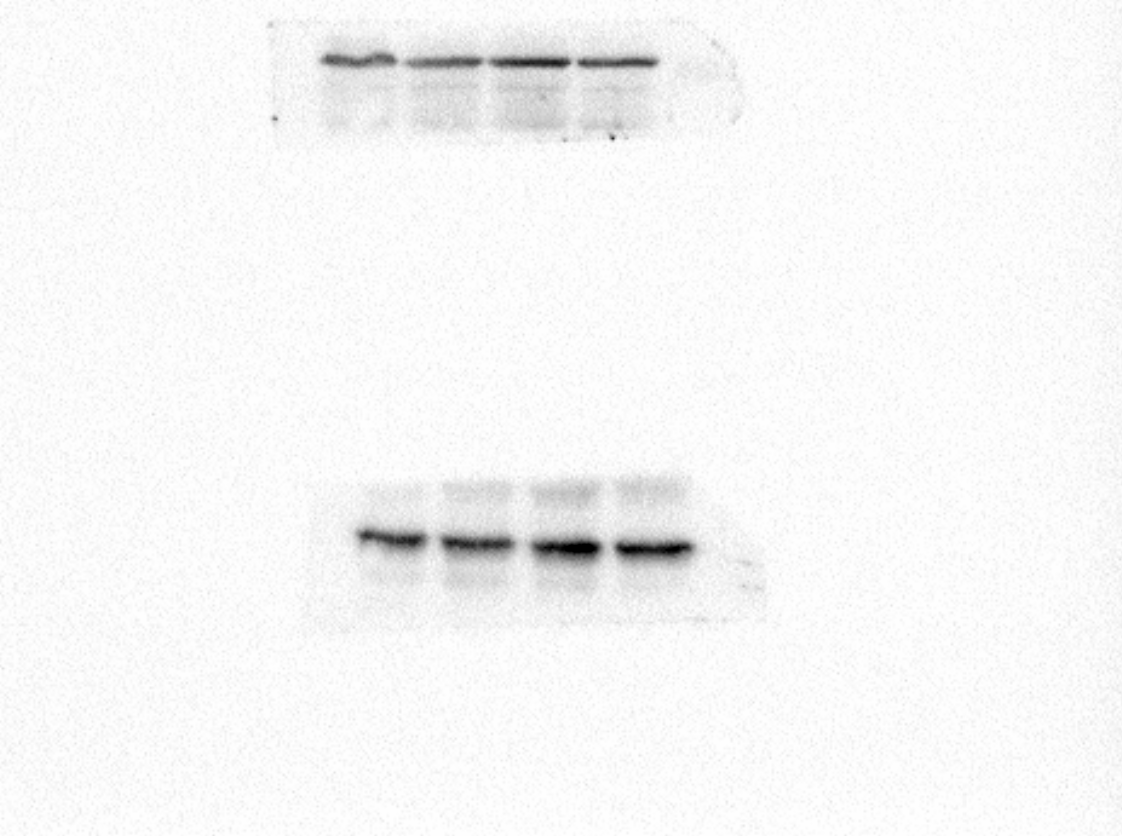

Supplement: Supplementary file 1 [file DataSheet1.zip › original WB images/26(GAPDH-pstat3) 54(GAPDH-pstat3).tif]

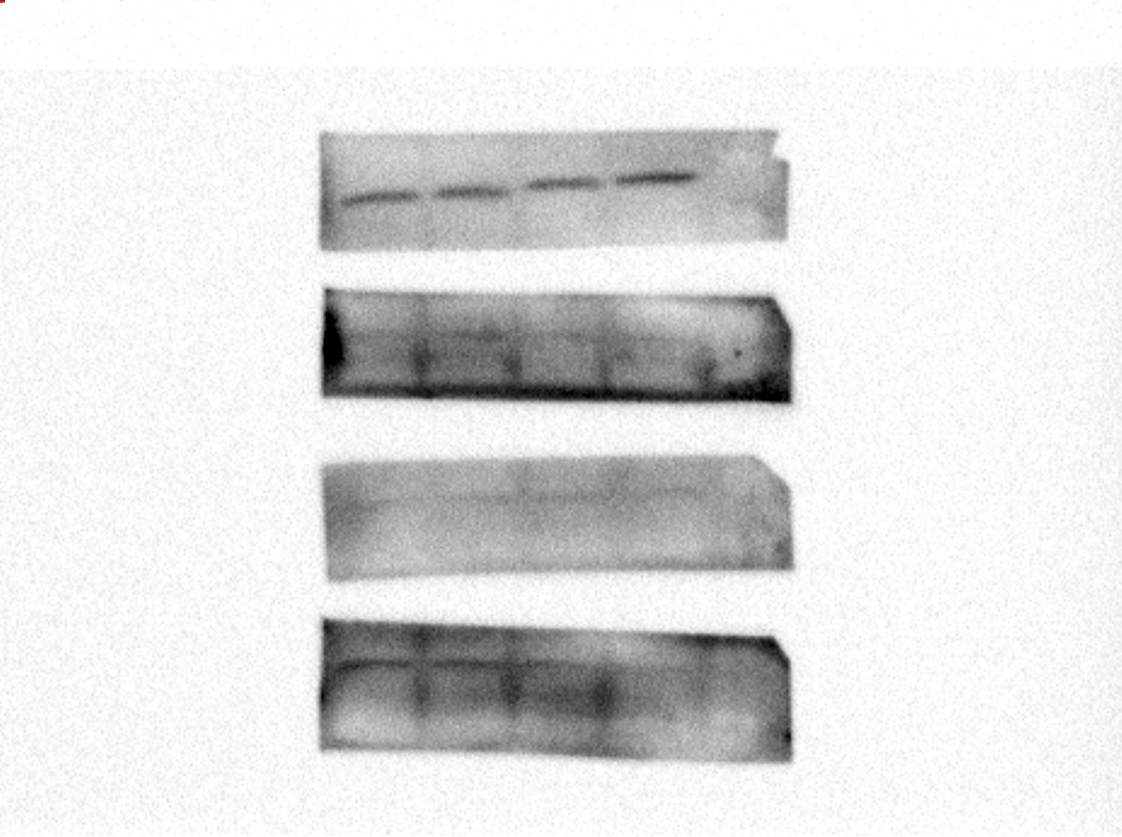

Supplement: Supplementary file 1 [file DataSheet1.zip › original WB images/28(GAPDH-pstat3) 46(JAK2-IEC6) 74(JAK2-IEC6) 75(p-JAK2-IEC6).tif]

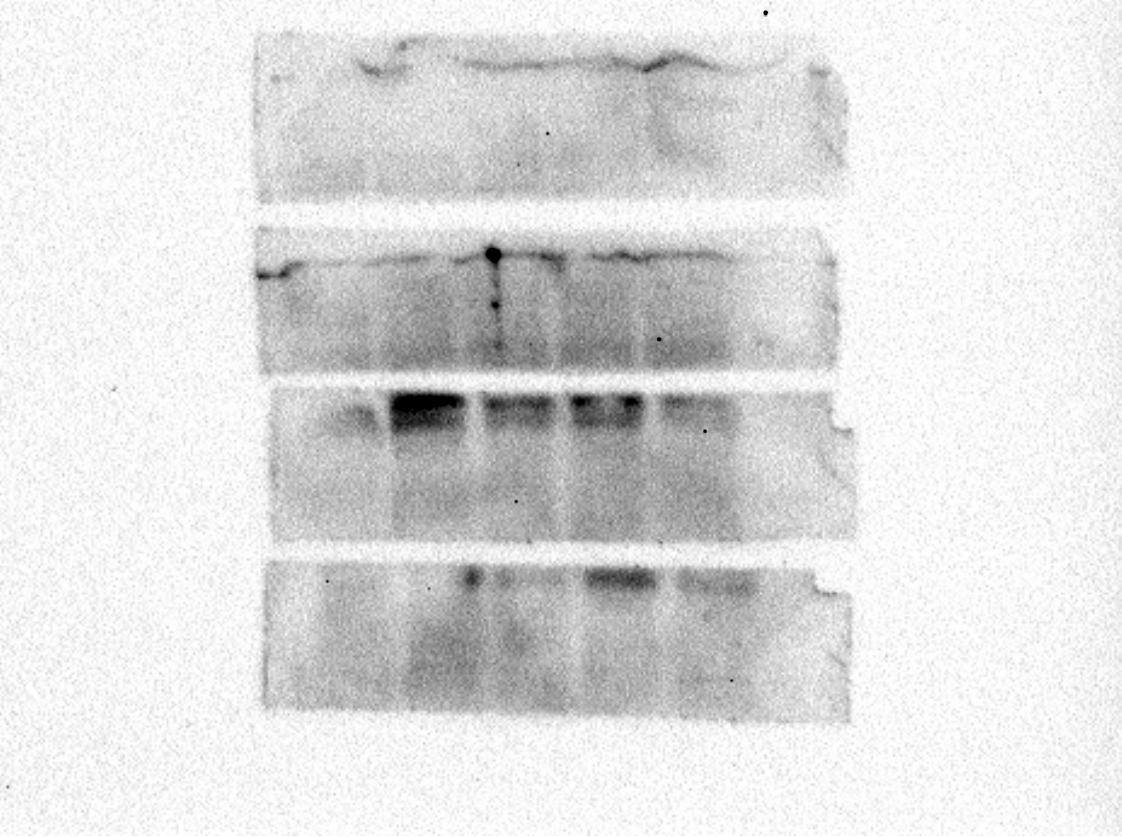

Supplement: Supplementary file 1 [file DataSheet1.zip › original WB images/29(COX2).tif]

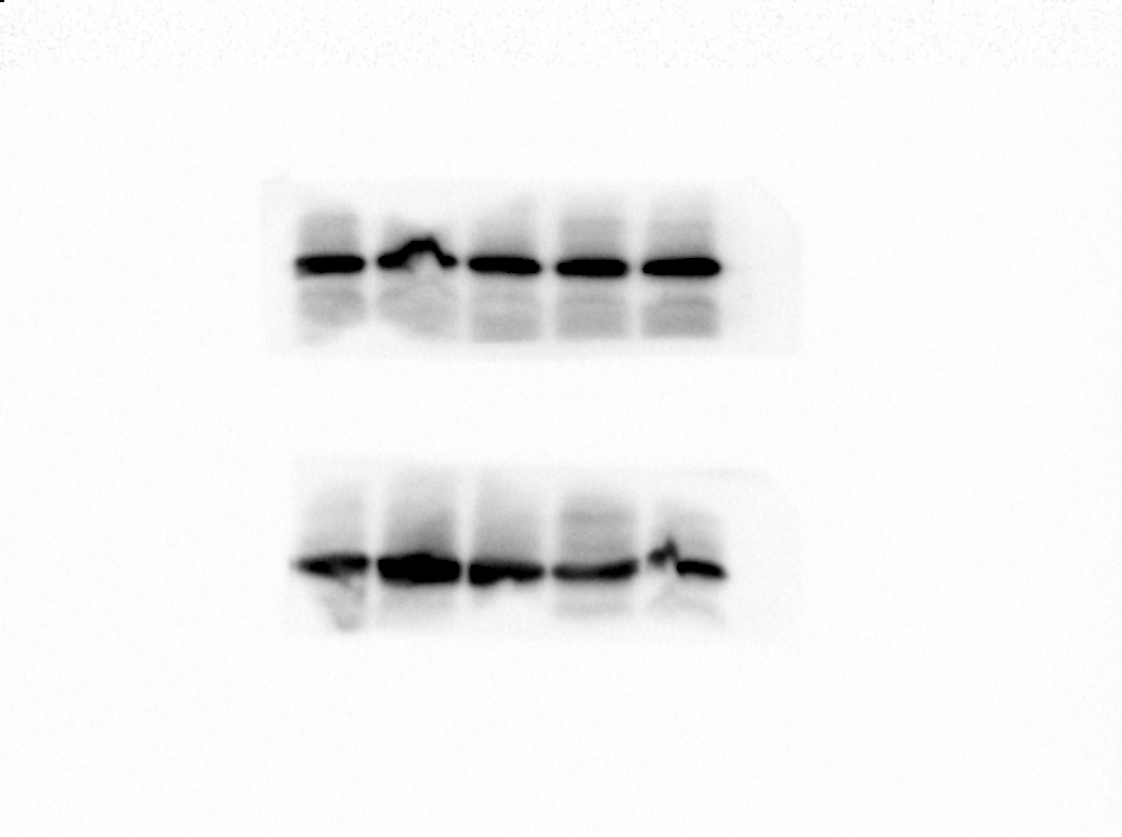

Supplement: Supplementary file 1 [file DataSheet1.zip › original WB images/30(GAPDH-COX2).tif]

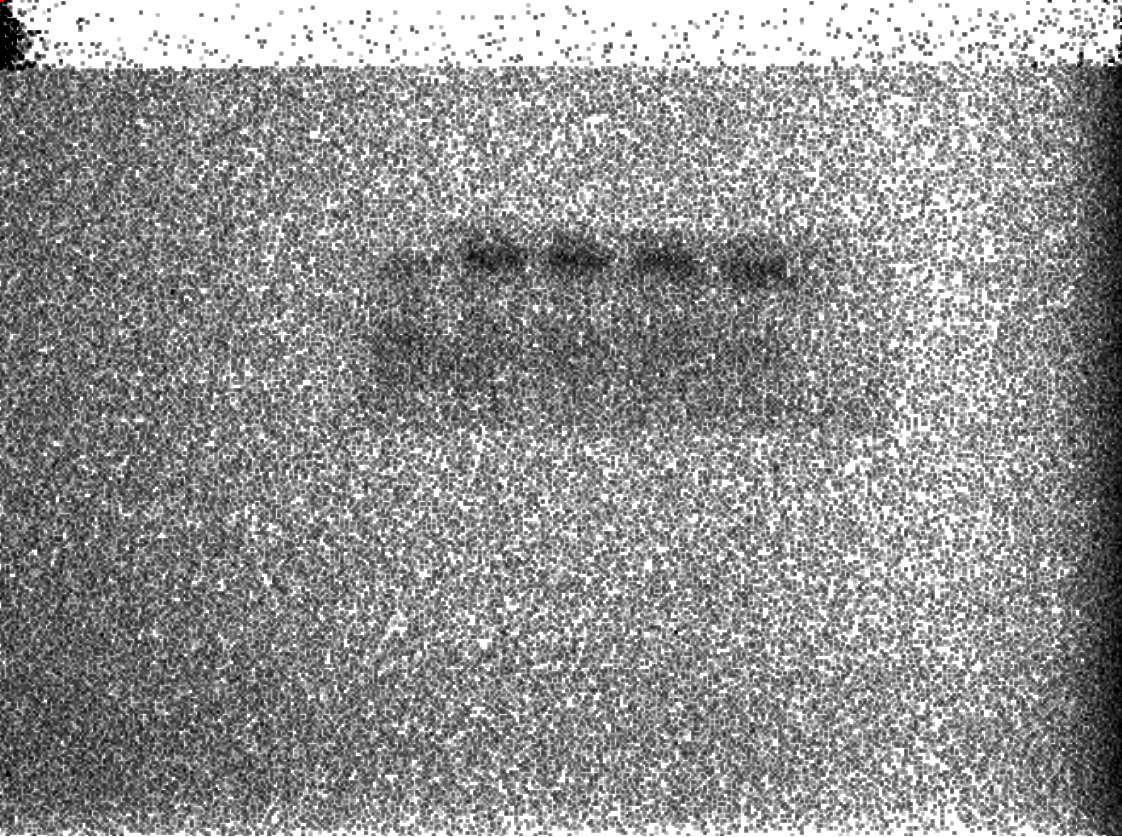

Supplement: Supplementary file 1 [file DataSheet1.zip › original WB images/31(iNOS).tif]

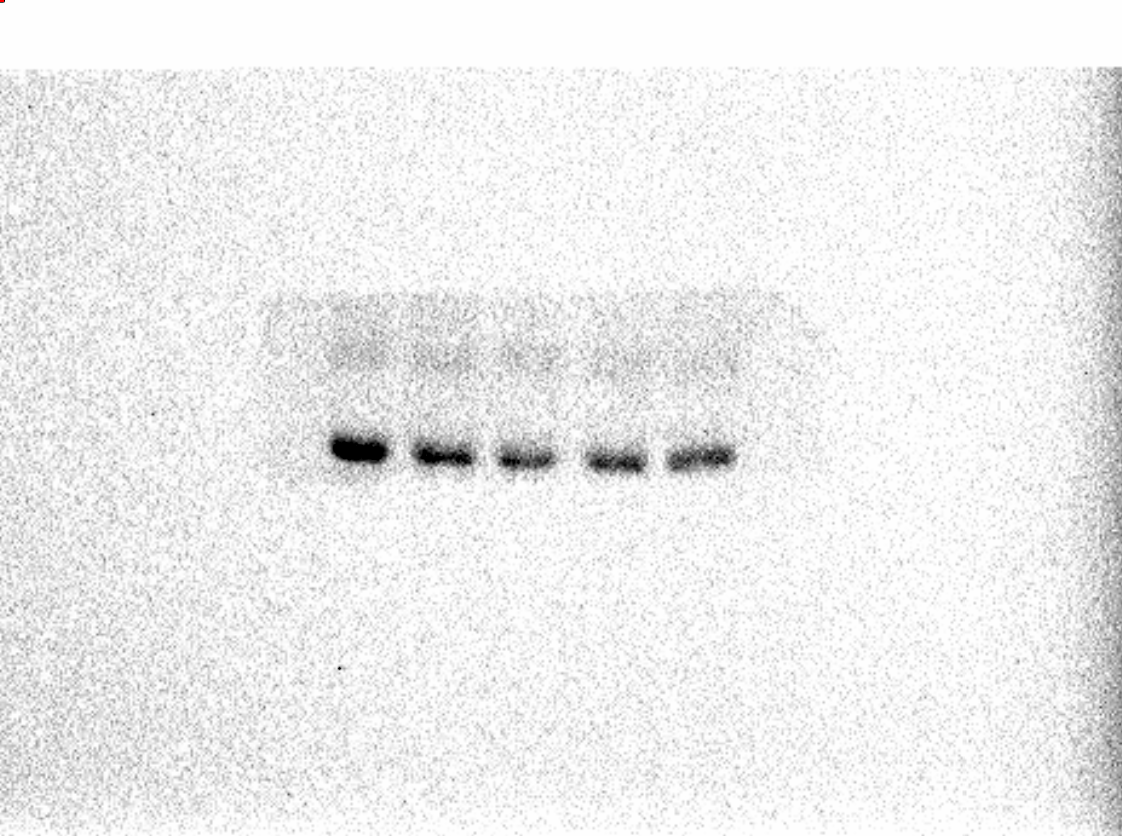

Supplement: Supplementary file 1 [file DataSheet1.zip › original WB images/32(GAPDH-iNOS).tif]

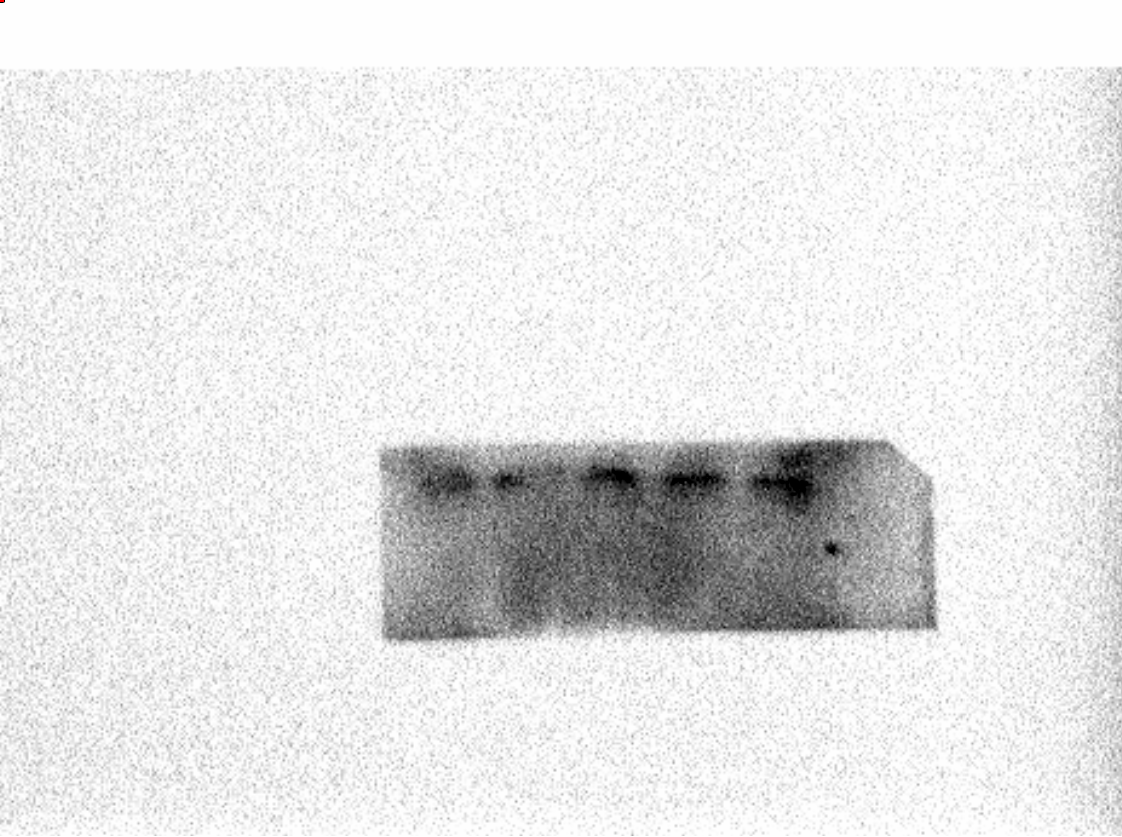

Supplement: Supplementary file 1 [file DataSheet1.zip › original WB images/33(bcl2).tif]

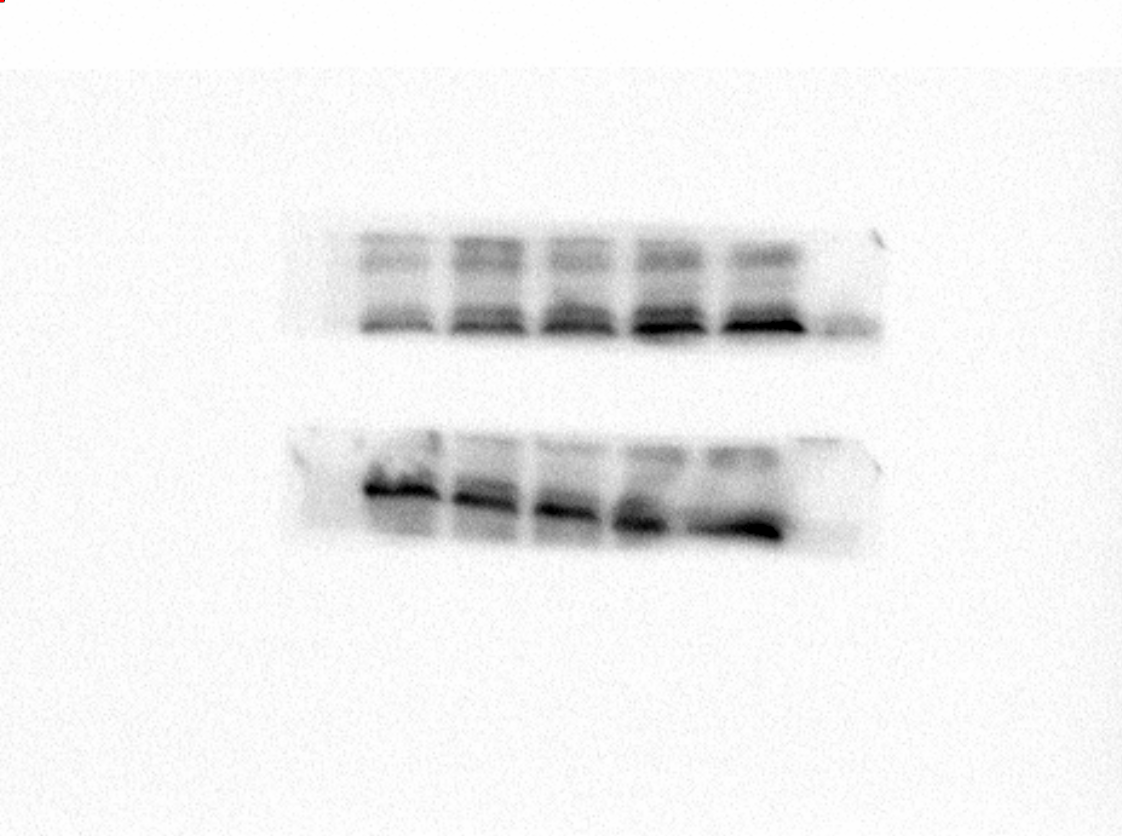

Supplement: Supplementary file 1 [file DataSheet1.zip › original WB images/34(GAPDH-bcl2) 62(GAPDH-bcl2).tif]

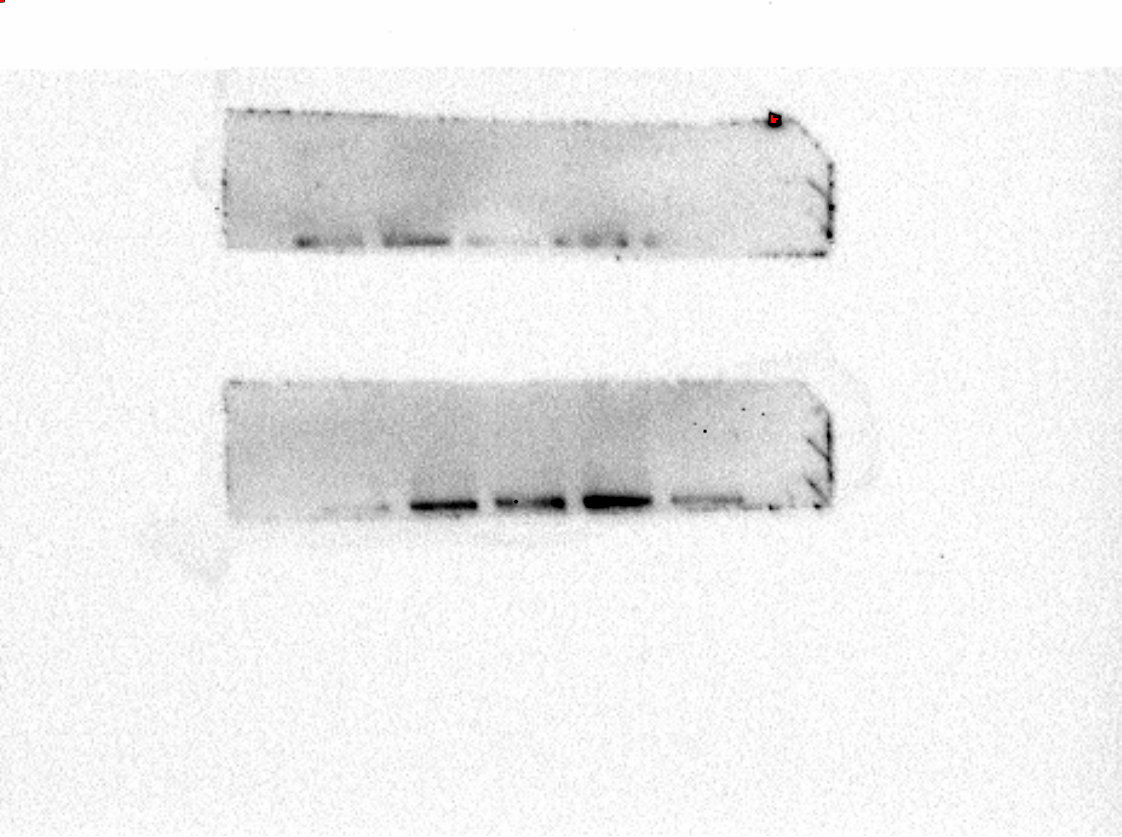

Supplement: Supplementary file 1 [file DataSheet1.zip › original WB images/35(MLCK).tif]

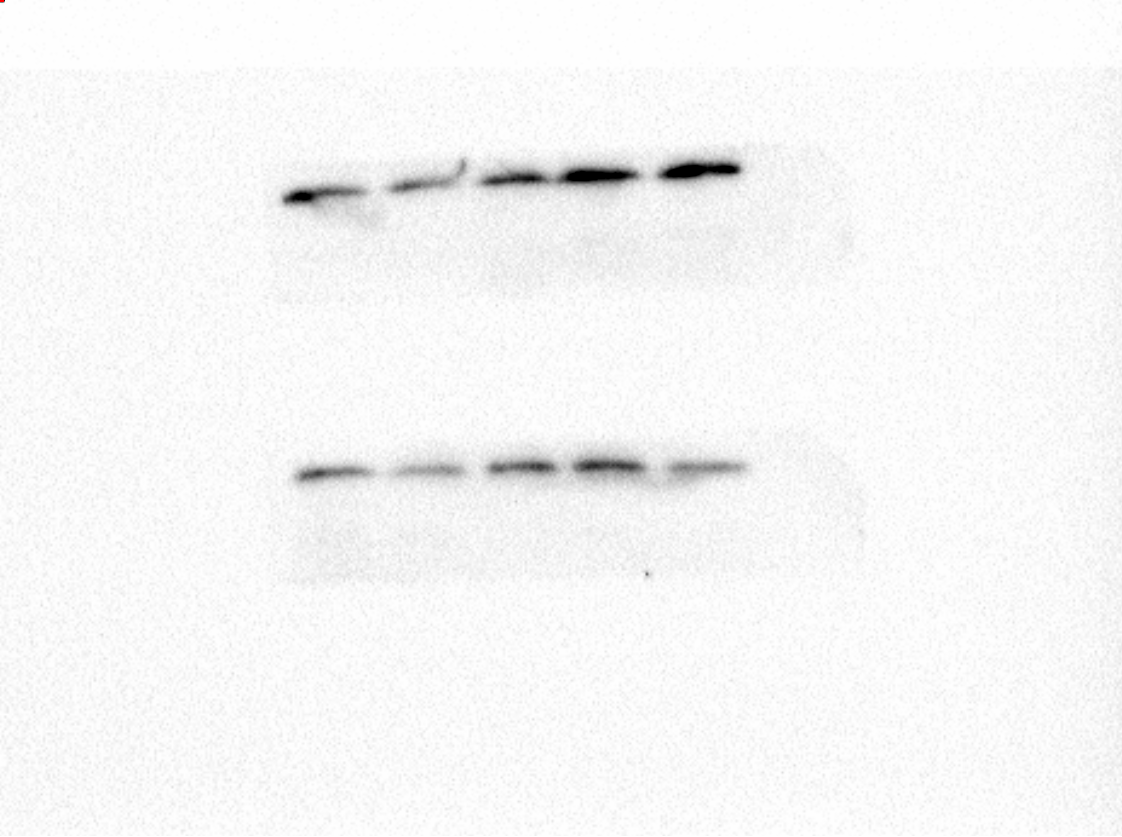

Supplement: Supplementary file 1 [file DataSheet1.zip › original WB images/36(GAPDH-MLCK) 60(GAPDH-iNOS).tif]

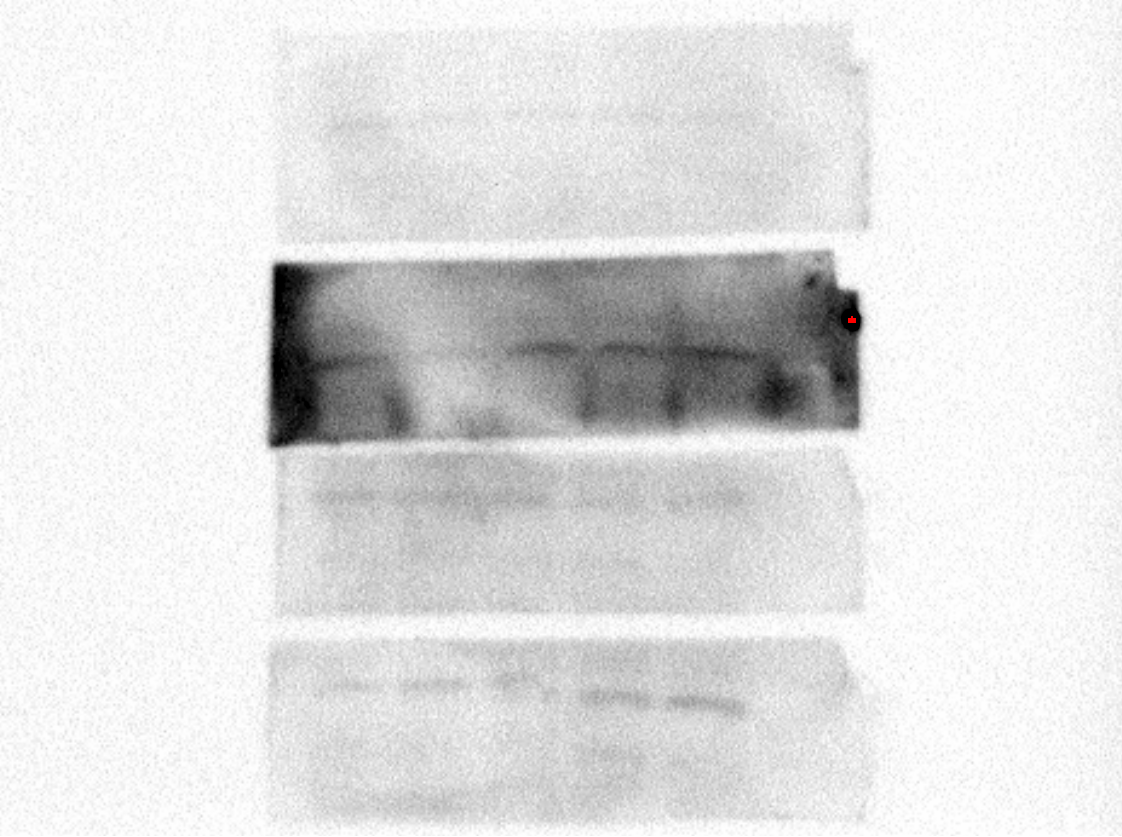

Supplement: Supplementary file 1 [file DataSheet1.zip › original WB images/37(JAK2).tif]

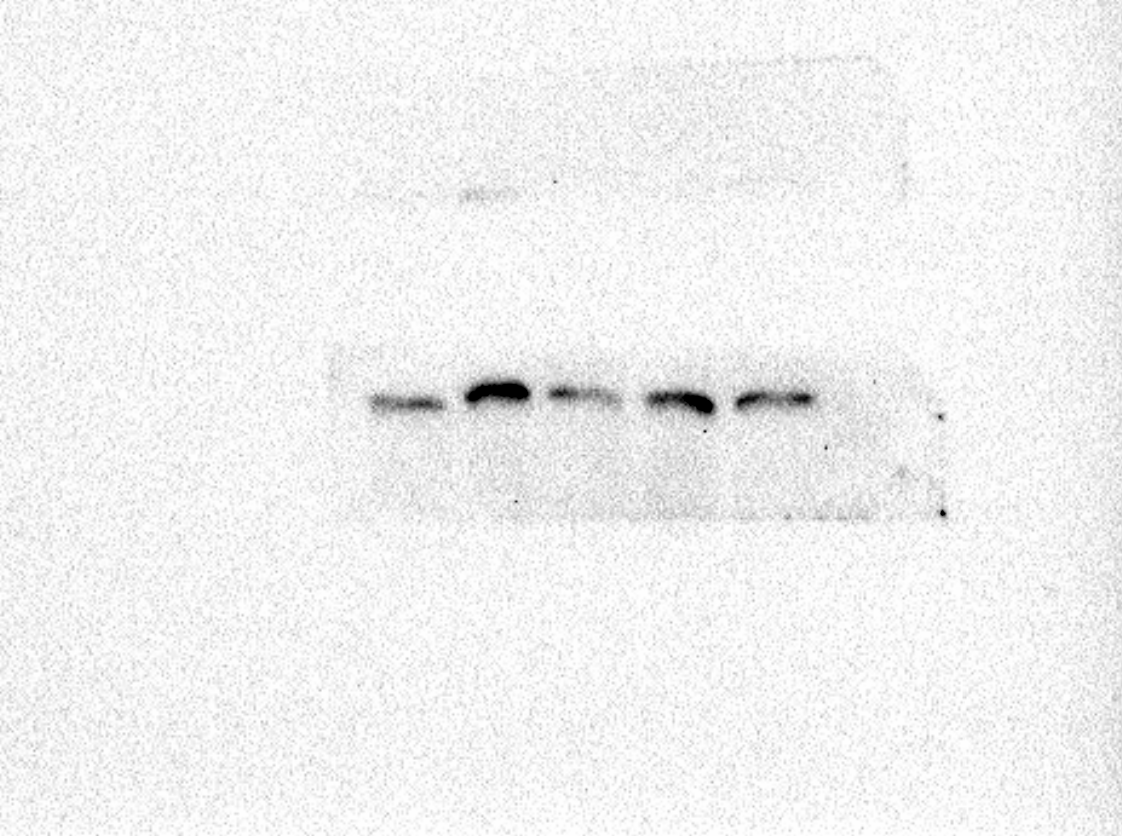

Supplement: Supplementary file 1 [file DataSheet1.zip › original WB images/39(p-JAK2).tif]

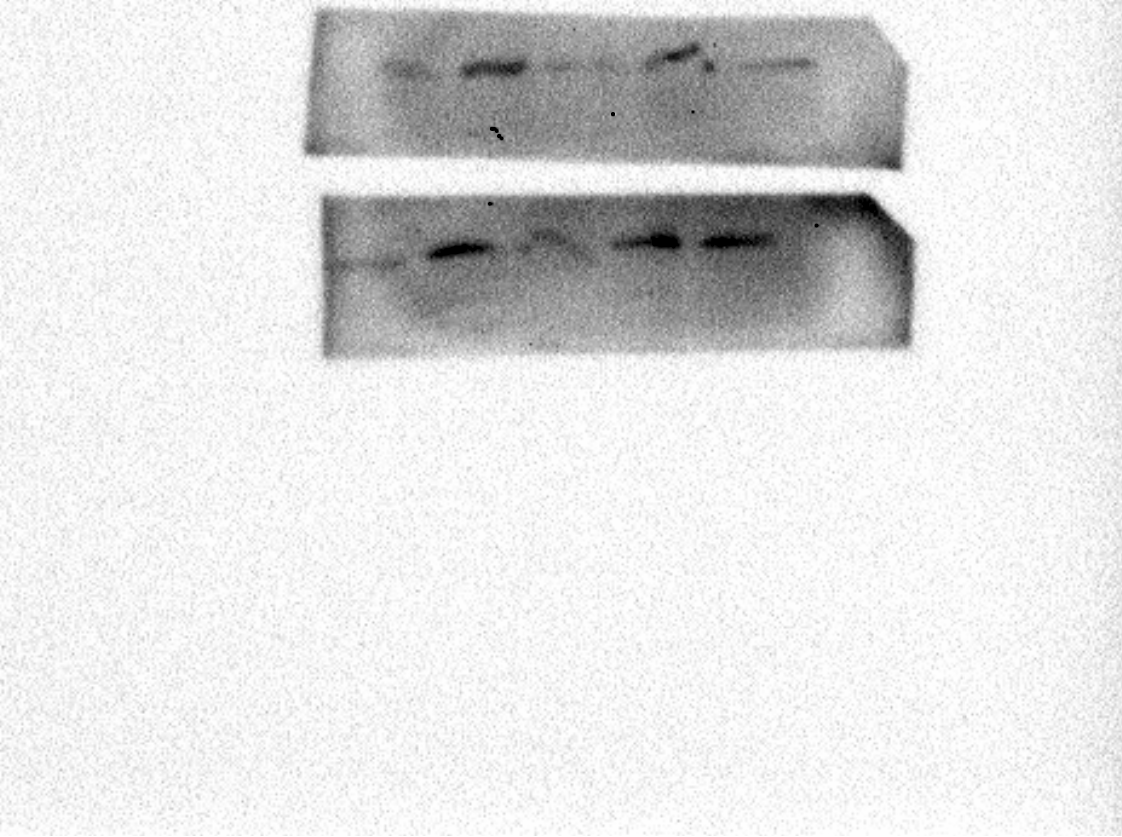

Supplement: Supplementary file 1 [file DataSheet1.zip › original WB images/41(p-STAT3).tif]

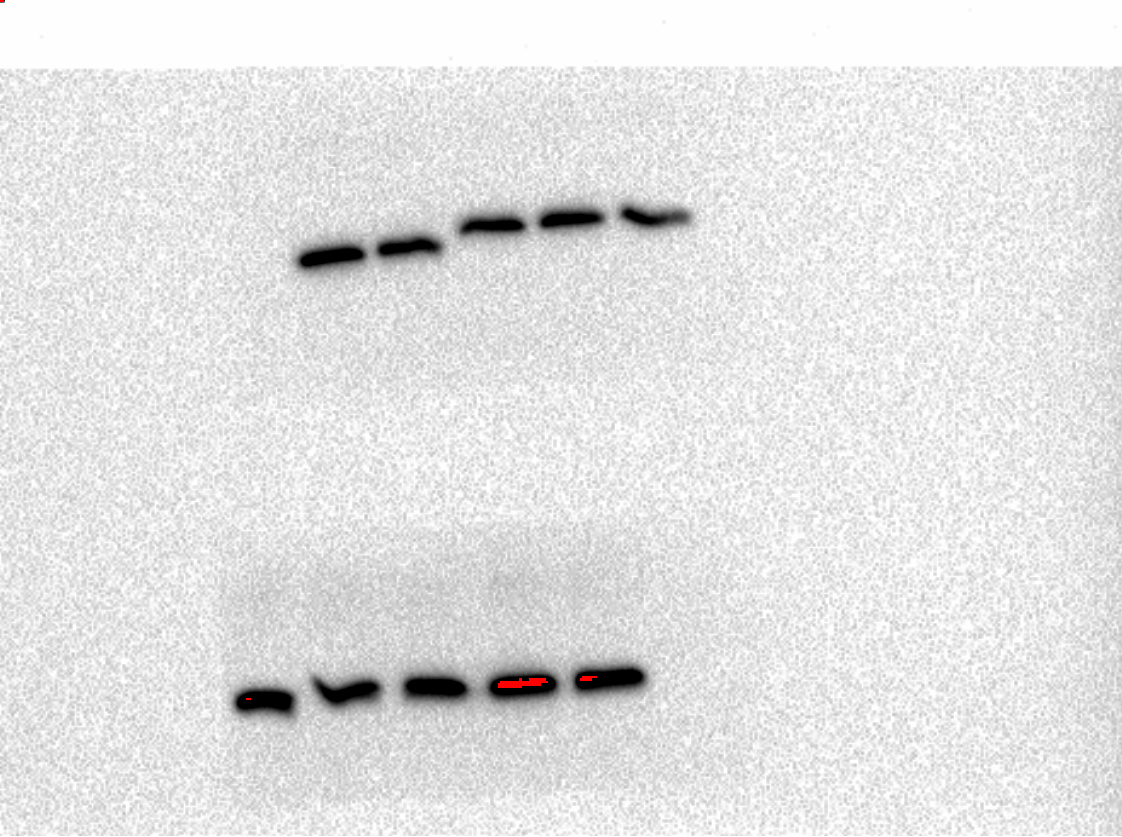

Supplement: Supplementary file 1 [file DataSheet1.zip › original WB images/42(GAPDH-pSTAT3).tif]

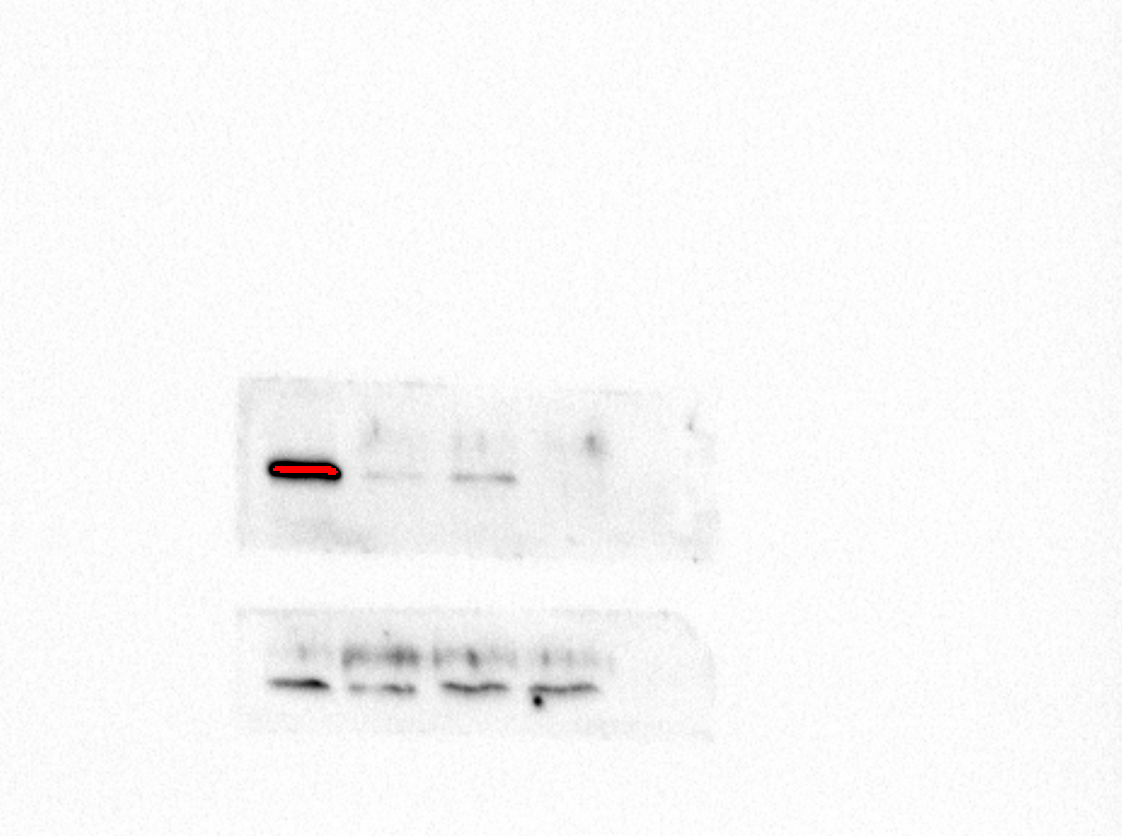

Supplement: Supplementary file 1 [file DataSheet1.zip › original WB images/43(JAK2-RAW).tif]

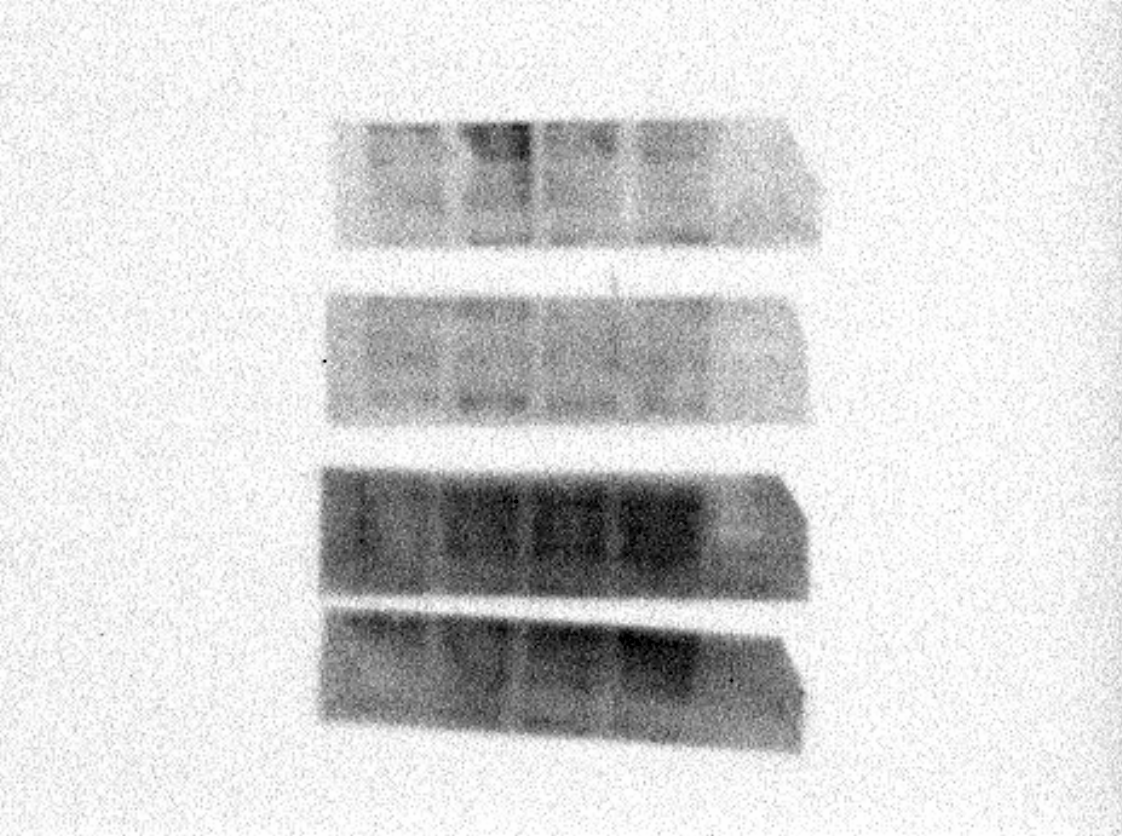

Supplement: Supplementary file 1 [file DataSheet1.zip › original WB images/44(p-JAK2-RAW).tif]

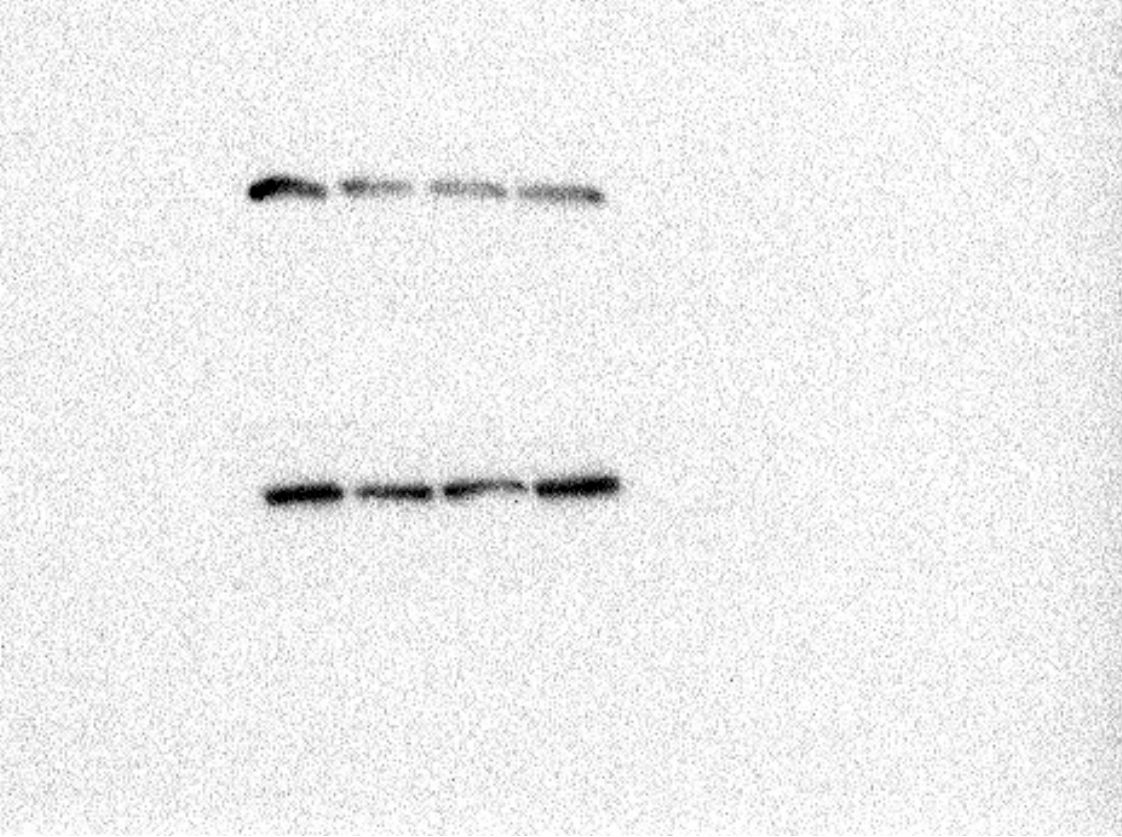

Supplement: Supplementary file 1 [file DataSheet1.zip › original WB images/45(GAPDH-RAW).tif]

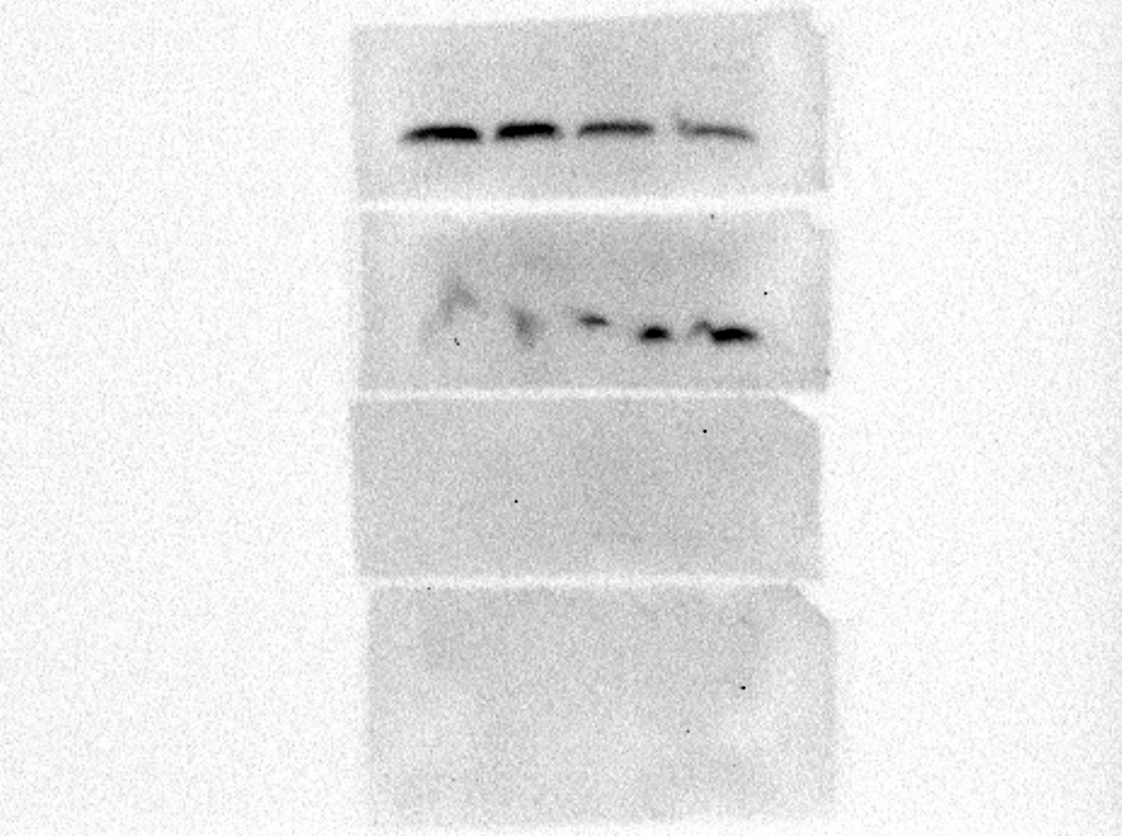

Supplement: Supplementary file 1 [file DataSheet1.zip › original WB images/48(GAPDH-IEC6).tif]

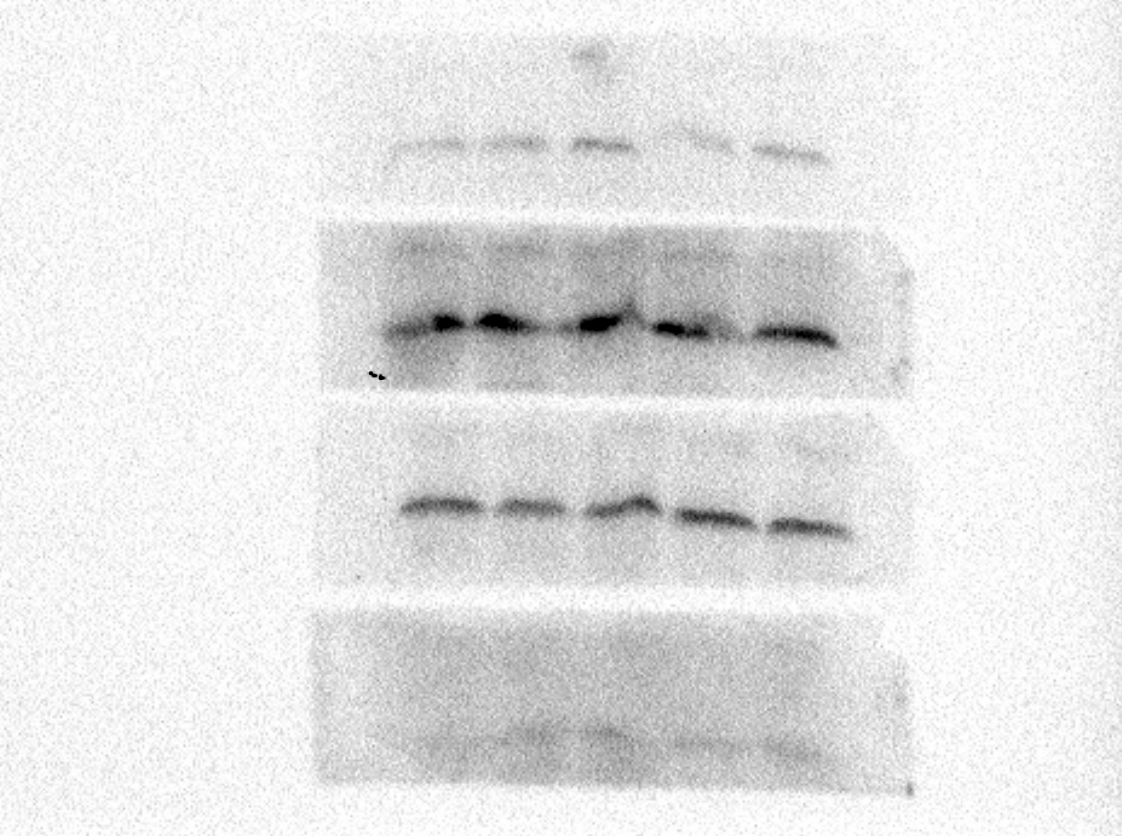

Supplement: Supplementary file 1 [file DataSheet1.zip › original WB images/49(JAK2) 50(GAPDH-JAK2) 77(JAK2) 78(GAPDH-JAK2).tif]

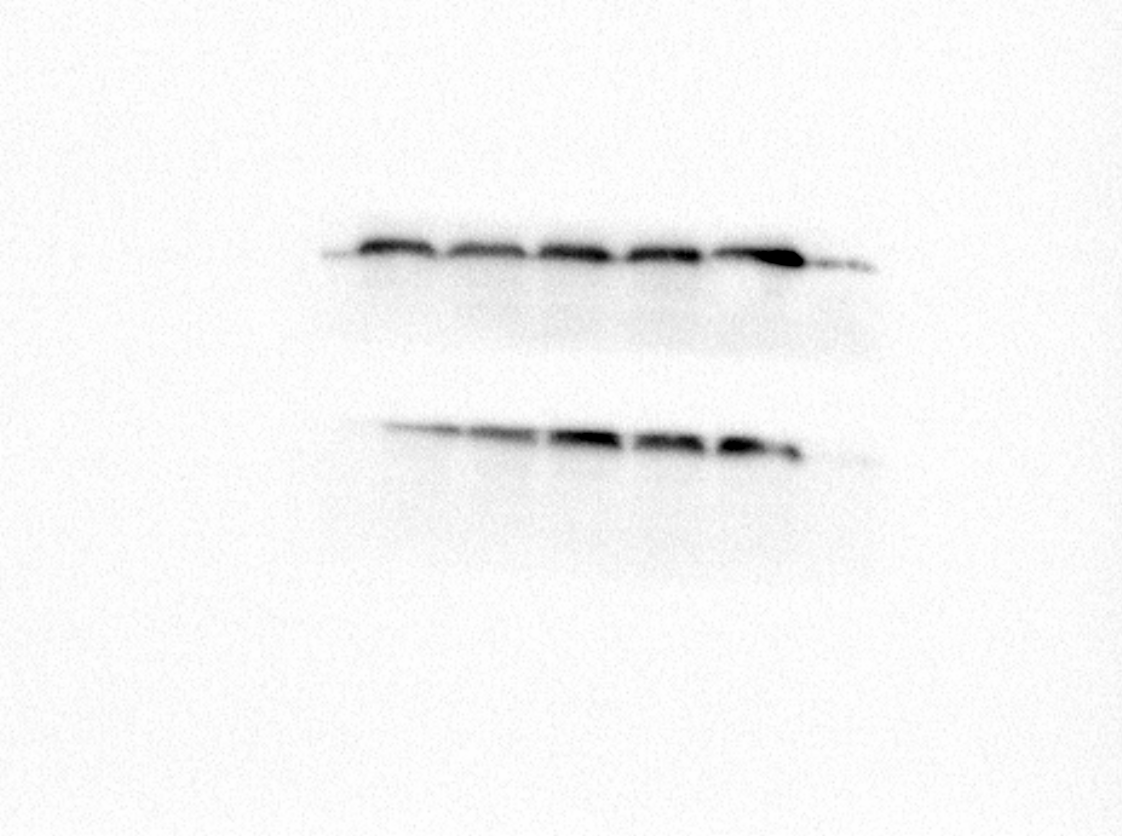

Supplement: Supplementary file 1 [file DataSheet1.zip › original WB images/5(Bcl-2).tif]

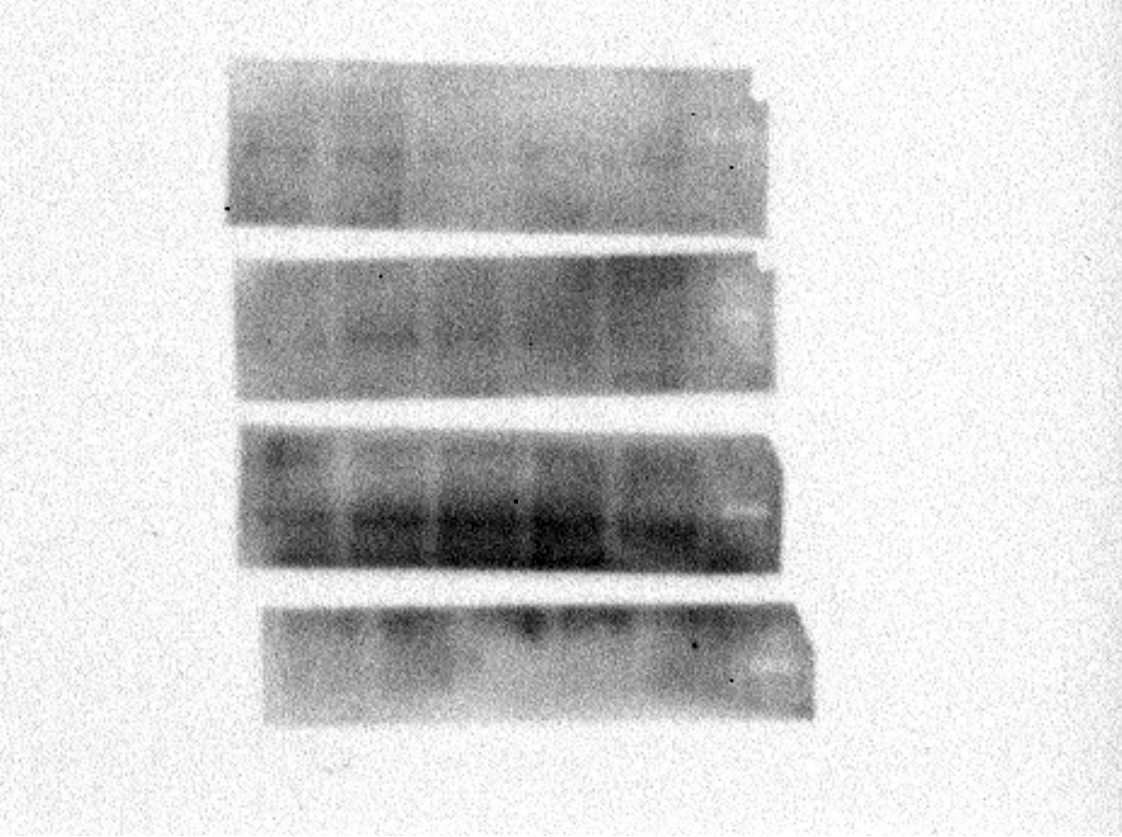

Supplement: Supplementary file 1 [file DataSheet1.zip › original WB images/51(p-JAK2) 52(GAPDH-pjak2).tif]

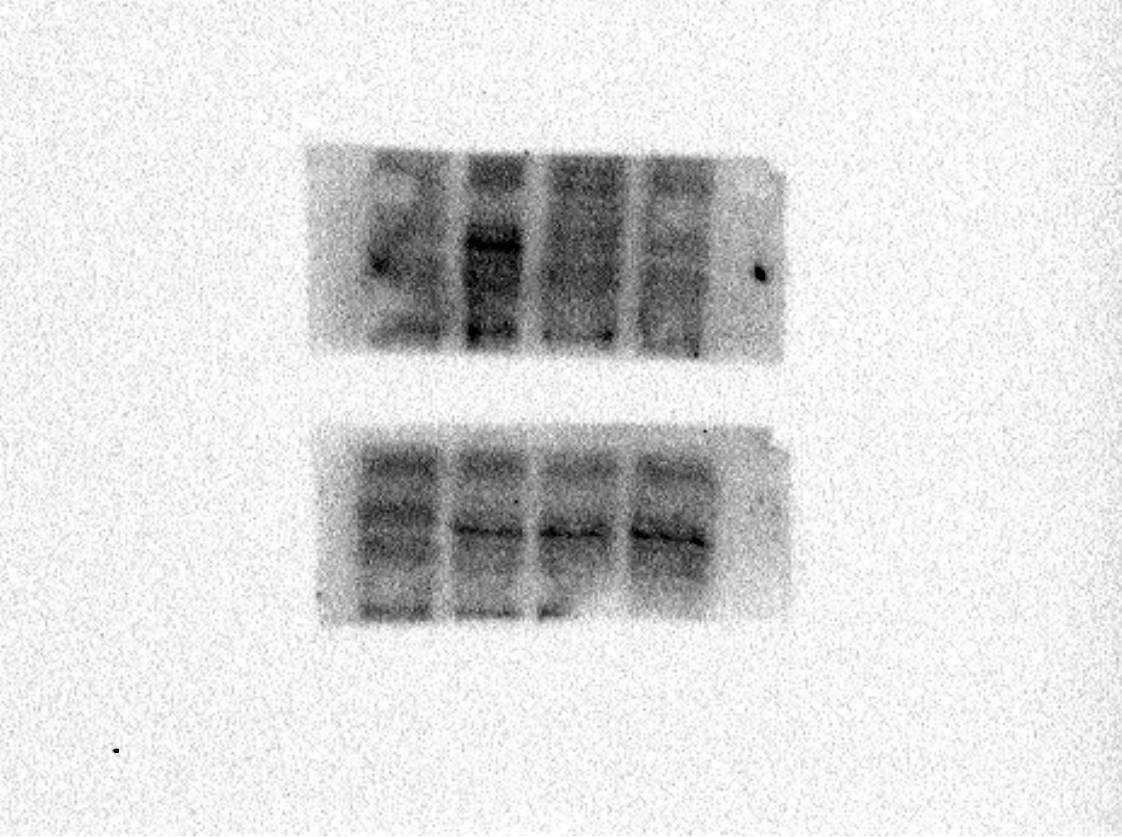

Supplement: Supplementary file 1 [file DataSheet1.zip › original WB images/55(p-STAT3).tif]

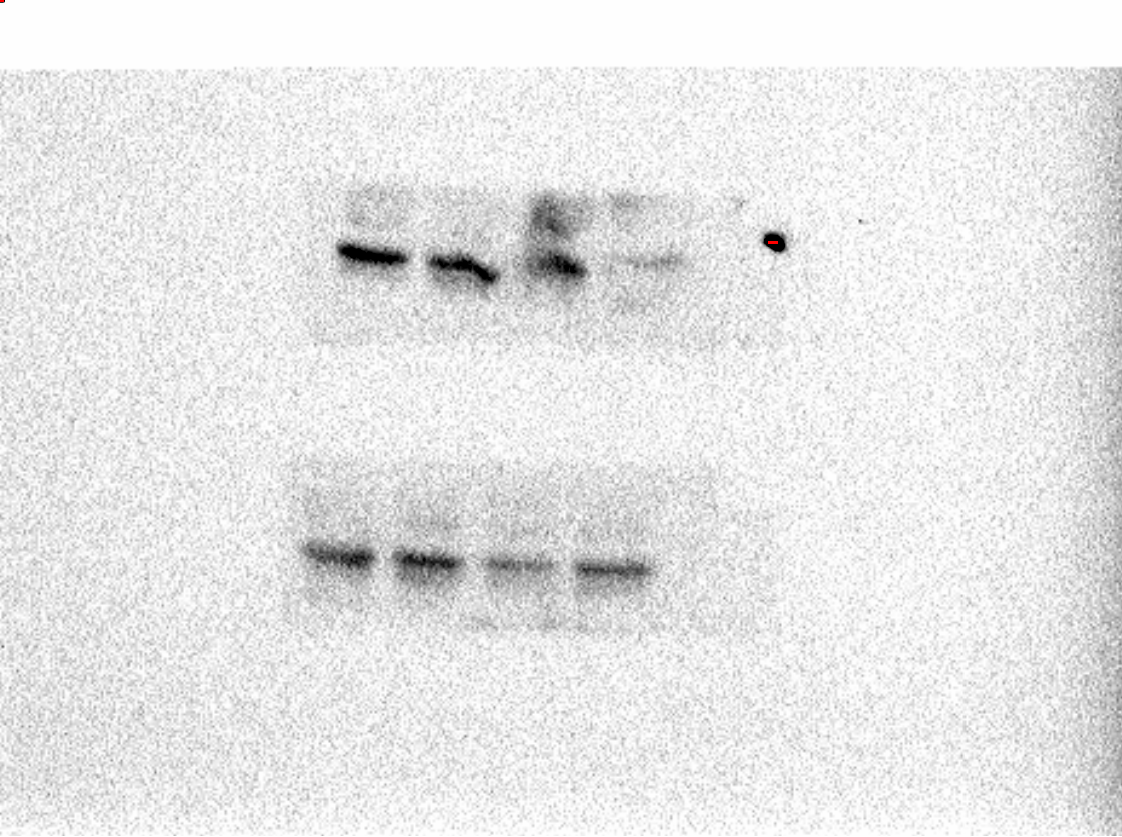

Supplement: Supplementary file 1 [file DataSheet1.zip › original WB images/56(GAPDH-pSTAT3).tif]

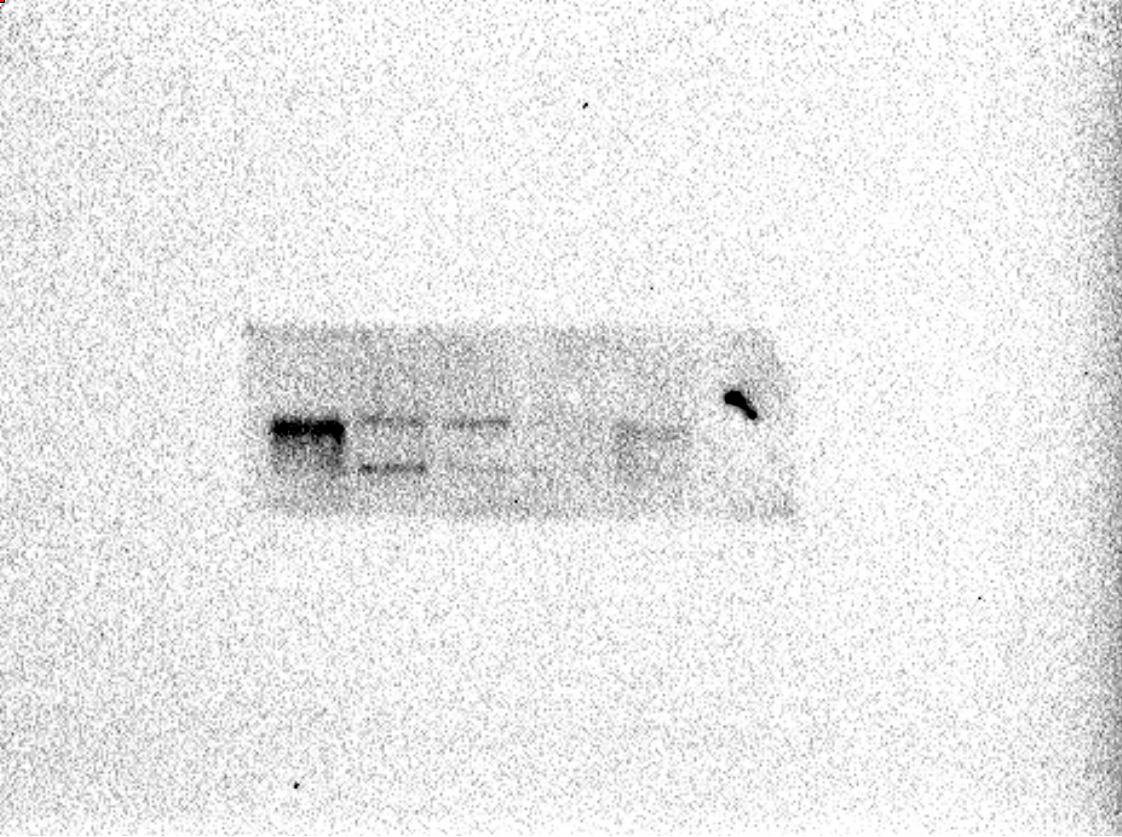

Supplement: Supplementary file 1 [file DataSheet1.zip › original WB images/57(COX2).tif]

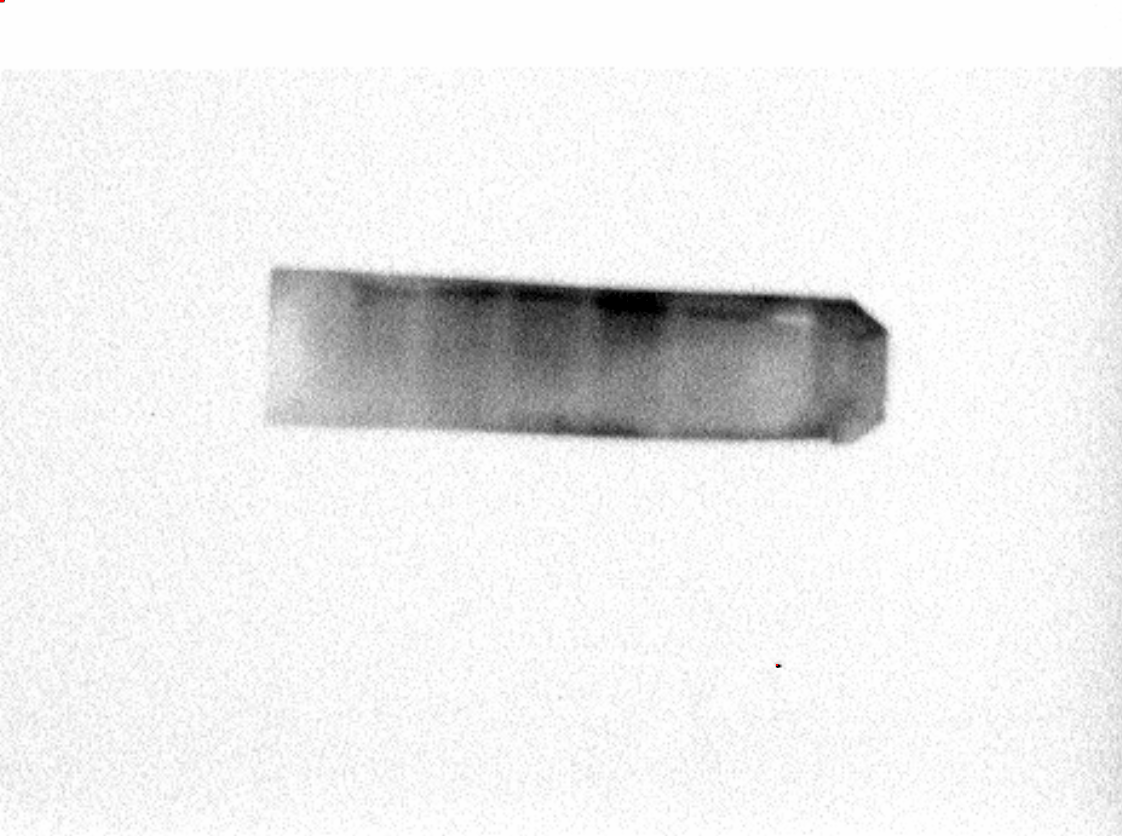

Supplement: Supplementary file 1 [file DataSheet1.zip › original WB images/58(GAPDH-COX2).tif]

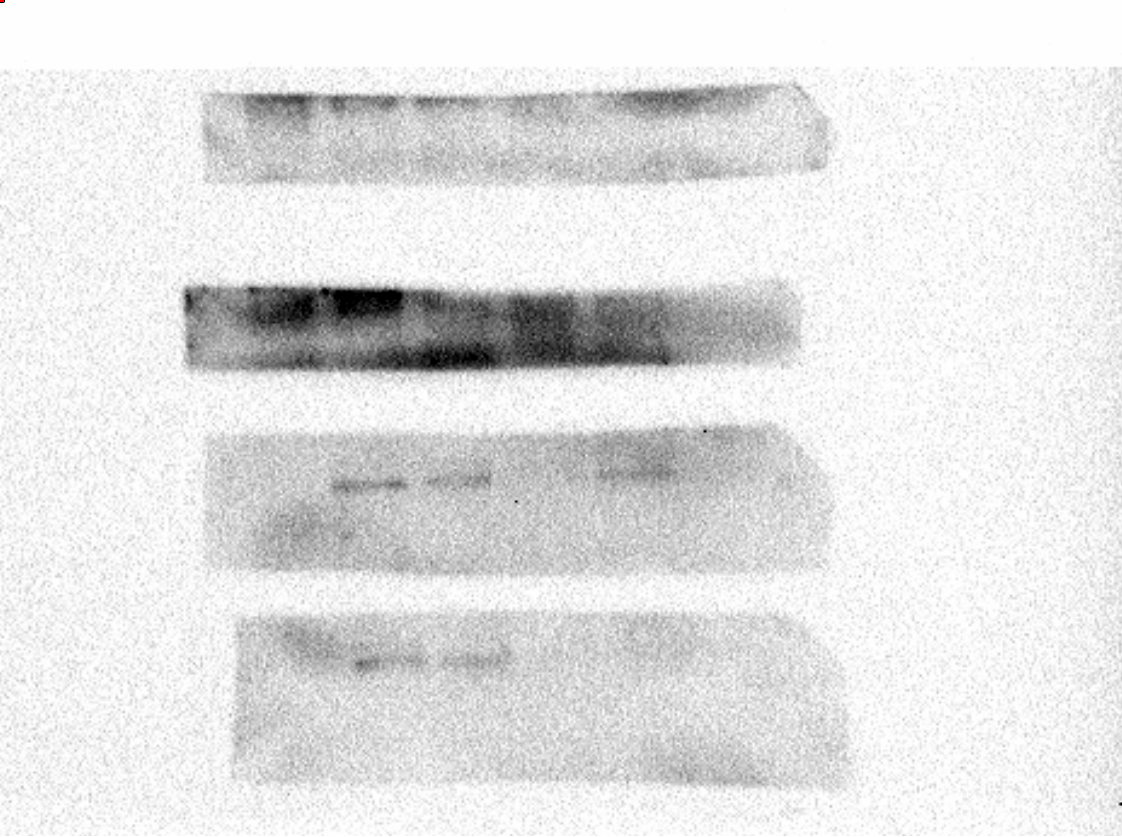

Supplement: Supplementary file 1 [file DataSheet1.zip › original WB images/59(iNOS).tif]

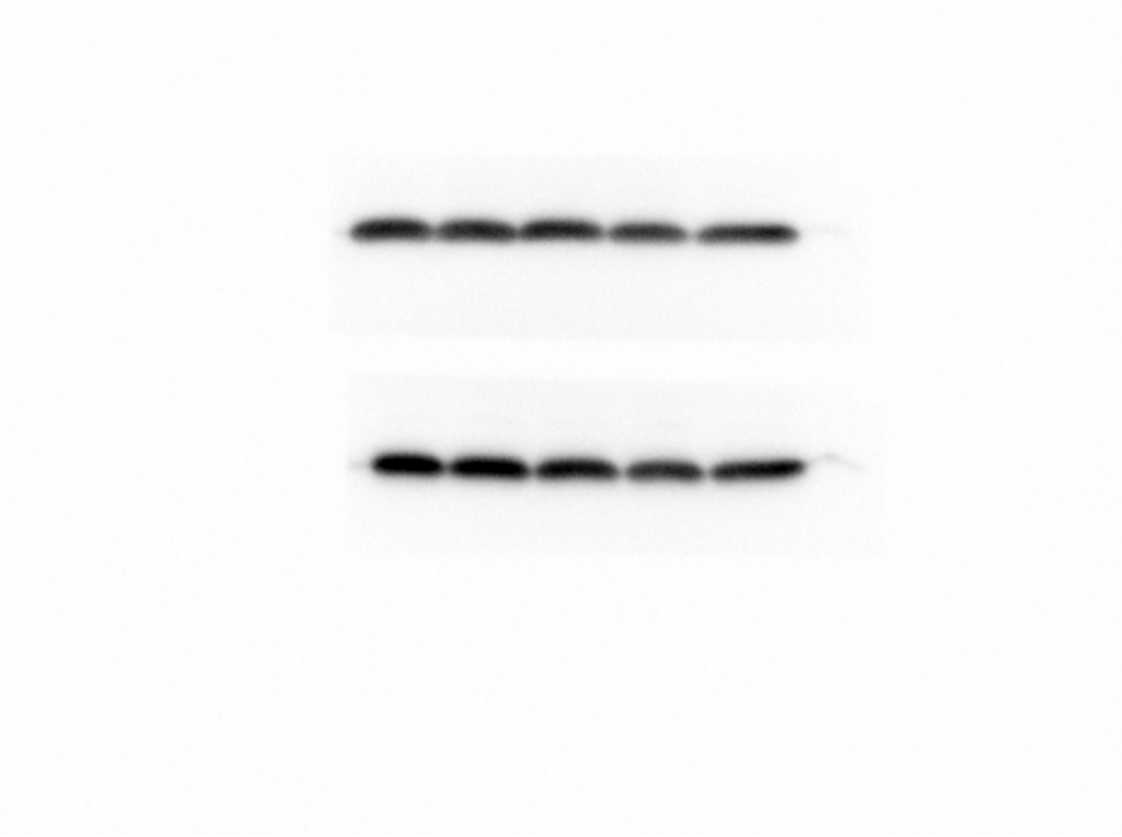

Supplement: Supplementary file 1 [file DataSheet1.zip › original WB images/6(GAPDH-Bcl2) 40(GAPDH-pjak2).tif]

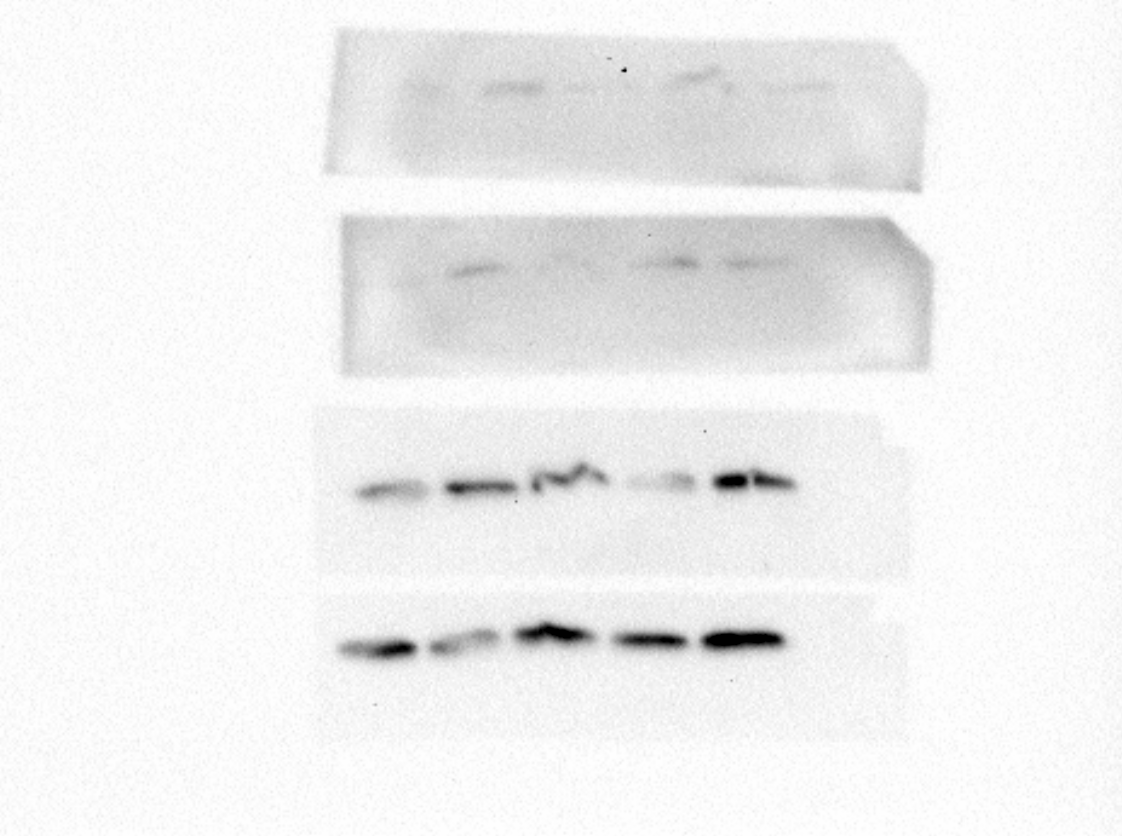

Supplement: Supplementary file 1 [file DataSheet1.zip › original WB images/61(Bcl-2).tif]

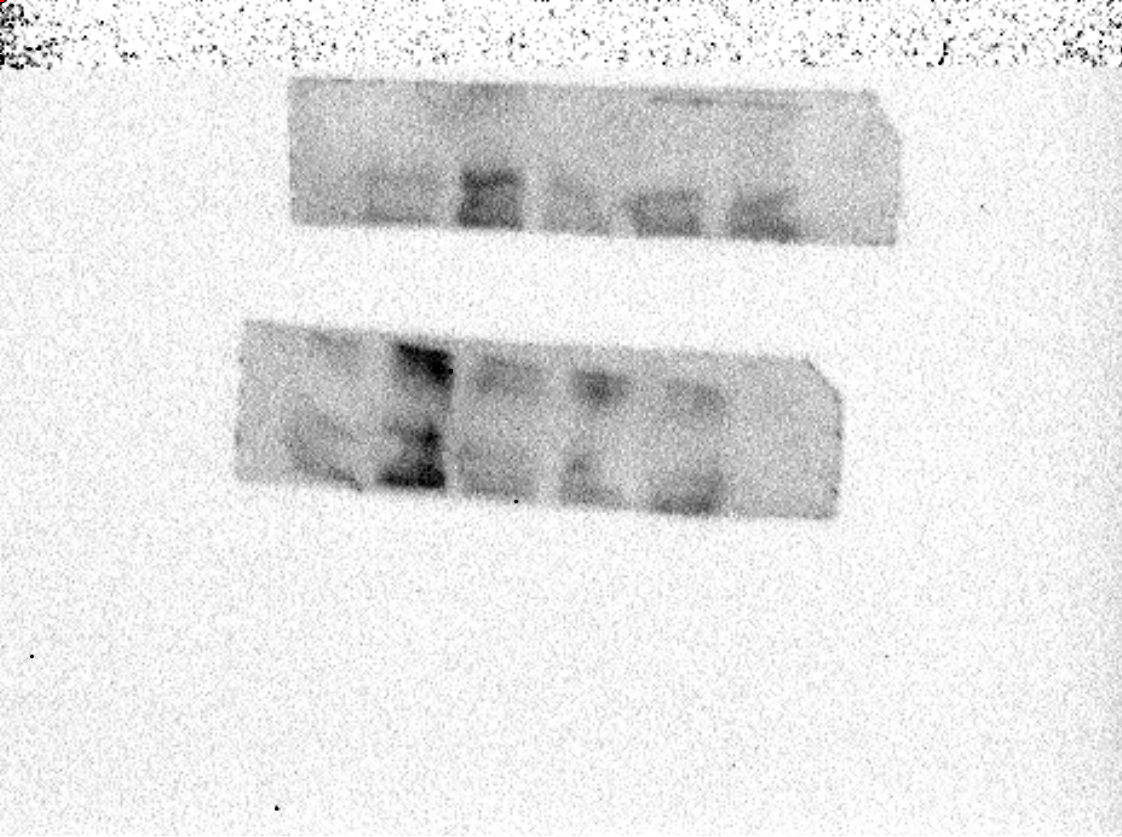

Supplement: Supplementary file 1 [file DataSheet1.zip › original WB images/63(MLCK).tif]

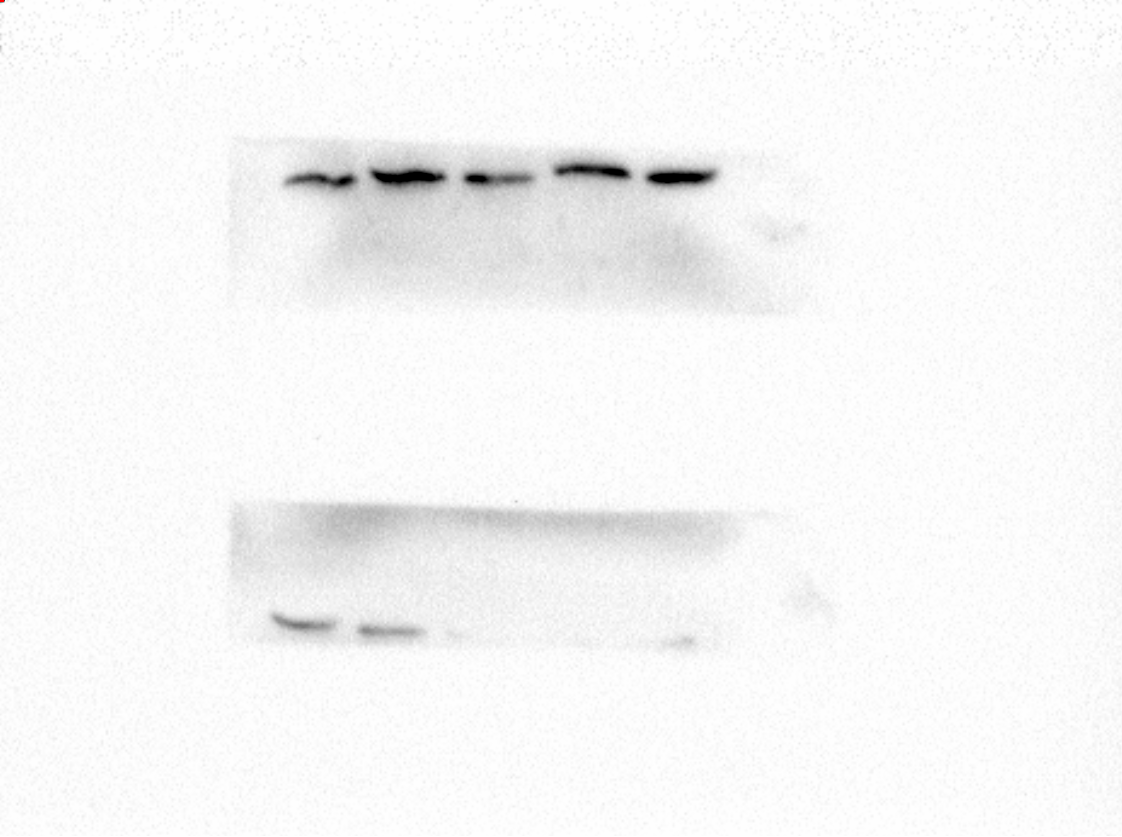

Supplement: Supplementary file 1 [file DataSheet1.zip › original WB images/64(GAPDH-MLCK).tif]

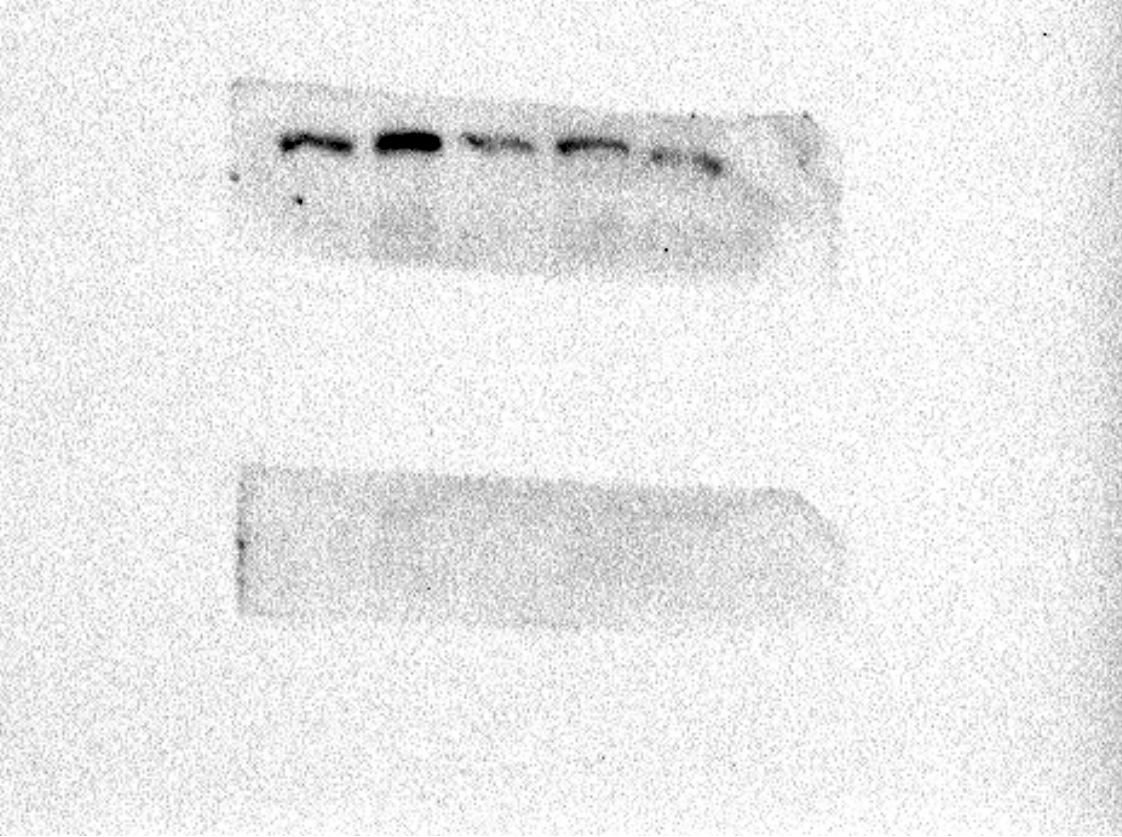

Supplement: Supplementary file 1 [file DataSheet1.zip › original WB images/67(p-JAK2).tif]

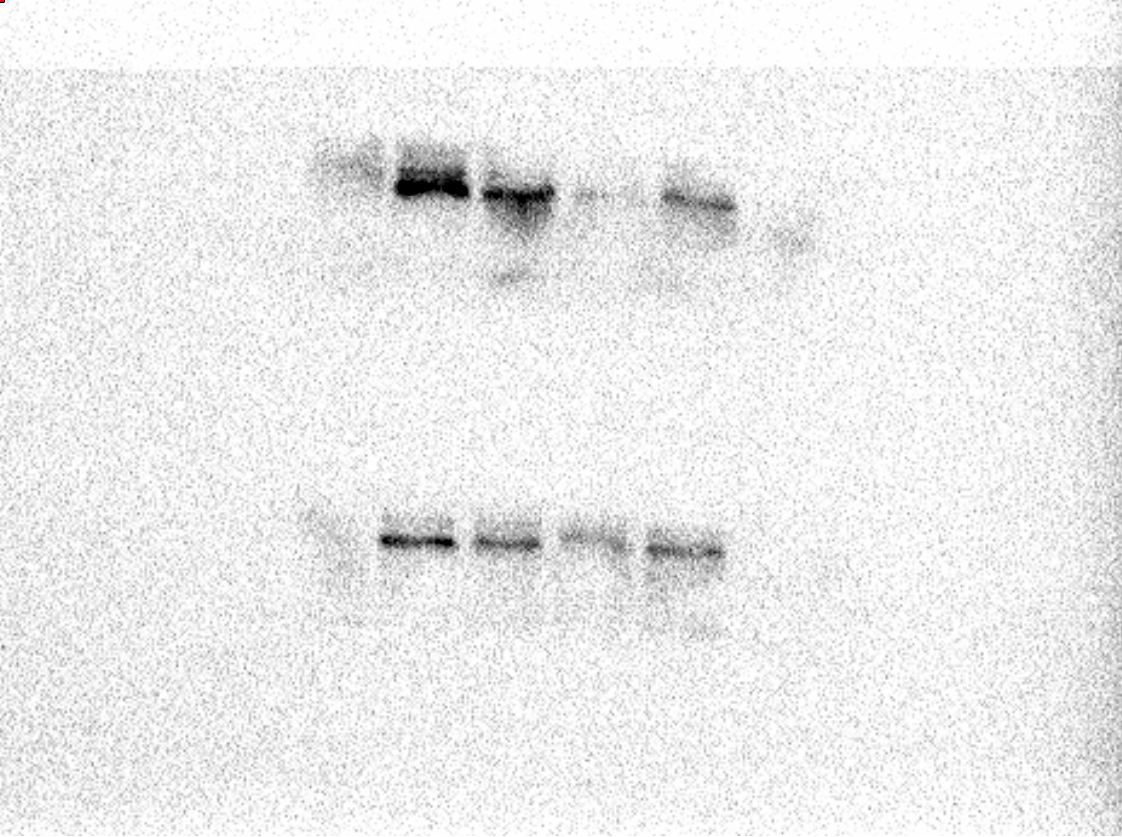

Supplement: Supplementary file 1 [file DataSheet1.zip › original WB images/69(p-STAT3).tif]

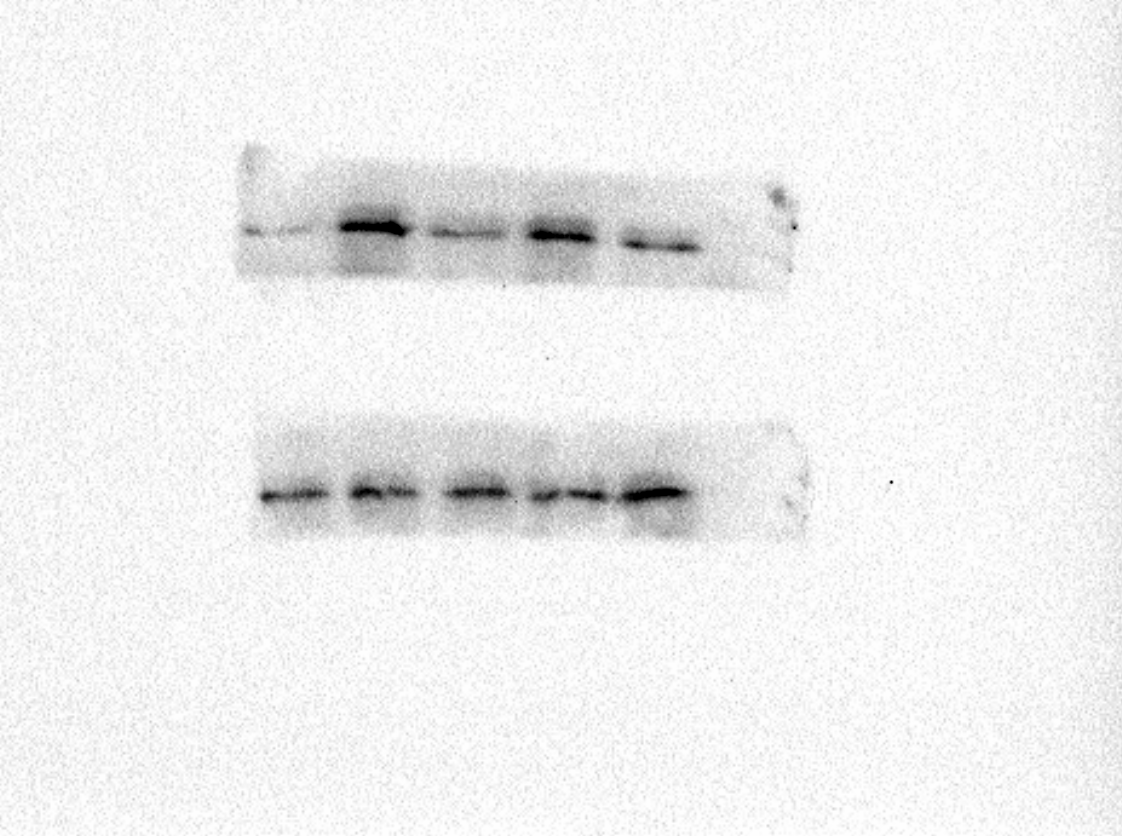

Supplement: Supplementary file 1 [file DataSheet1.zip › original WB images/7(MLCK) 68(GAPDH-p-jak2).tif]

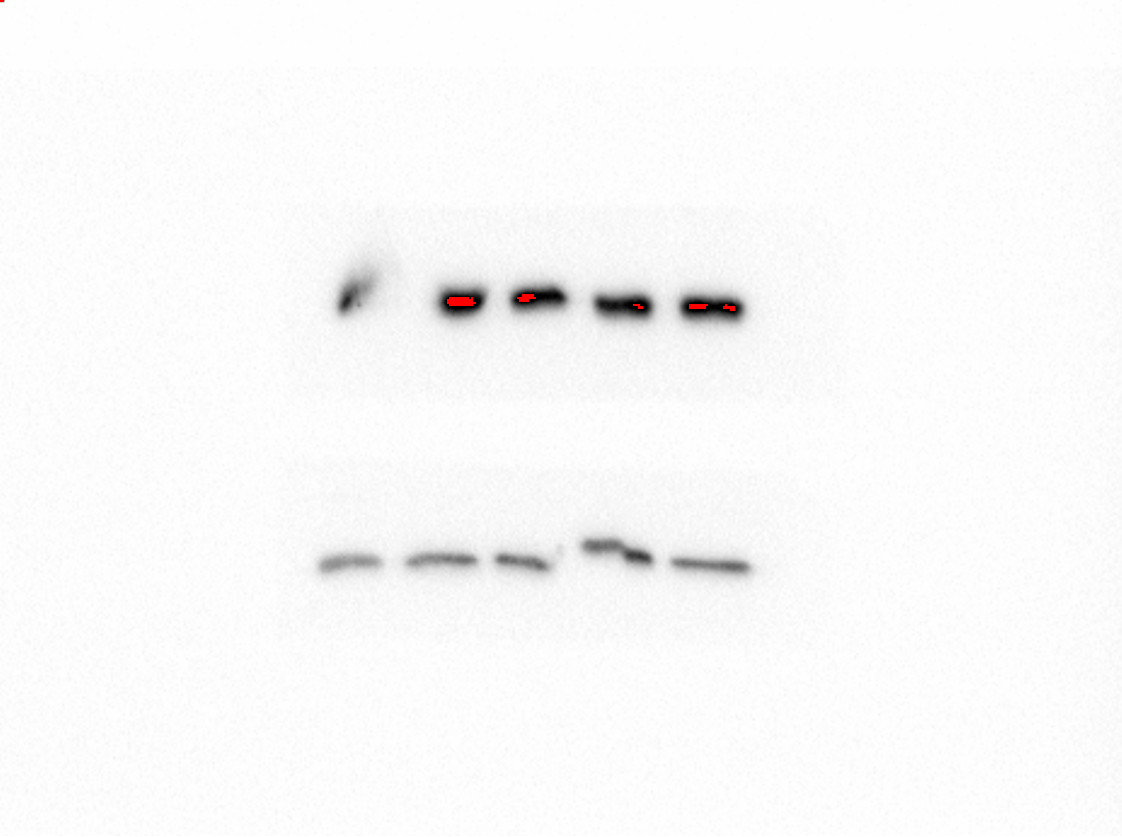

Supplement: Supplementary file 1 [file DataSheet1.zip › original WB images/70(GAPDH-pSTAT3).tif]

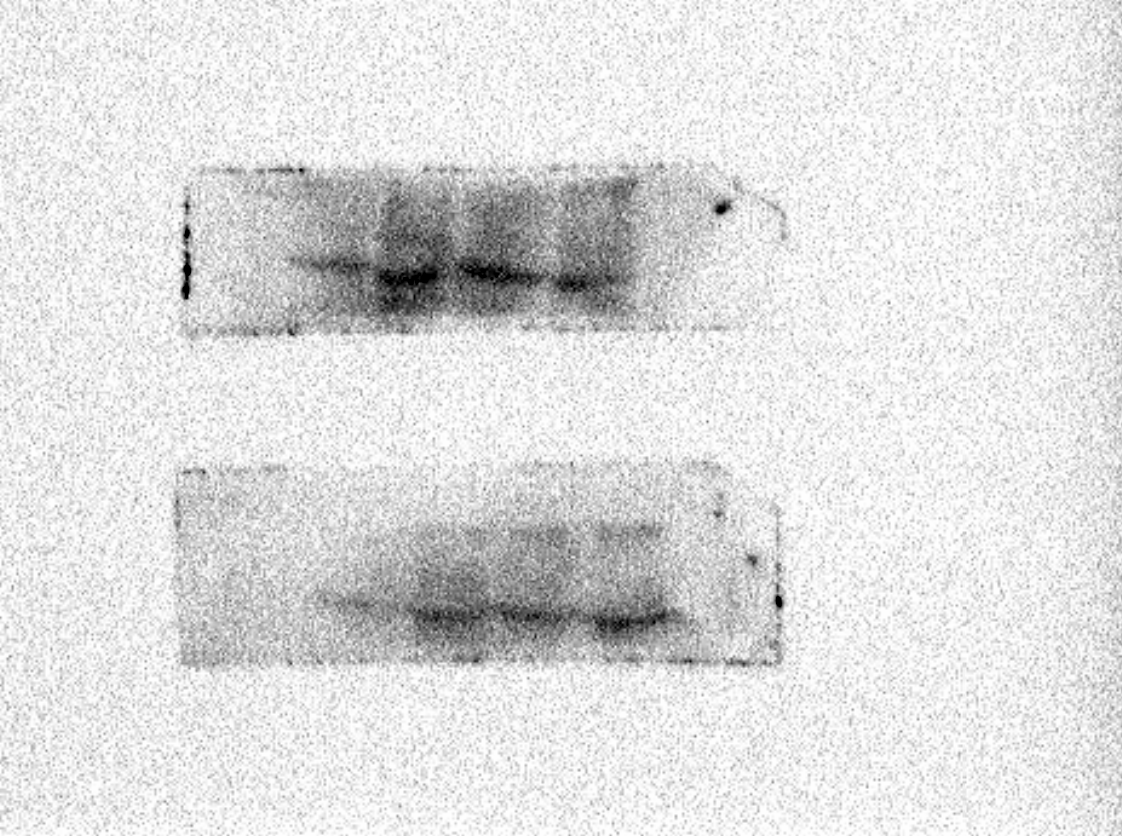

Supplement: Supplementary file 1 [file DataSheet1.zip › original WB images/71(JAK2-RAW) 83(p-STAT3).tif]

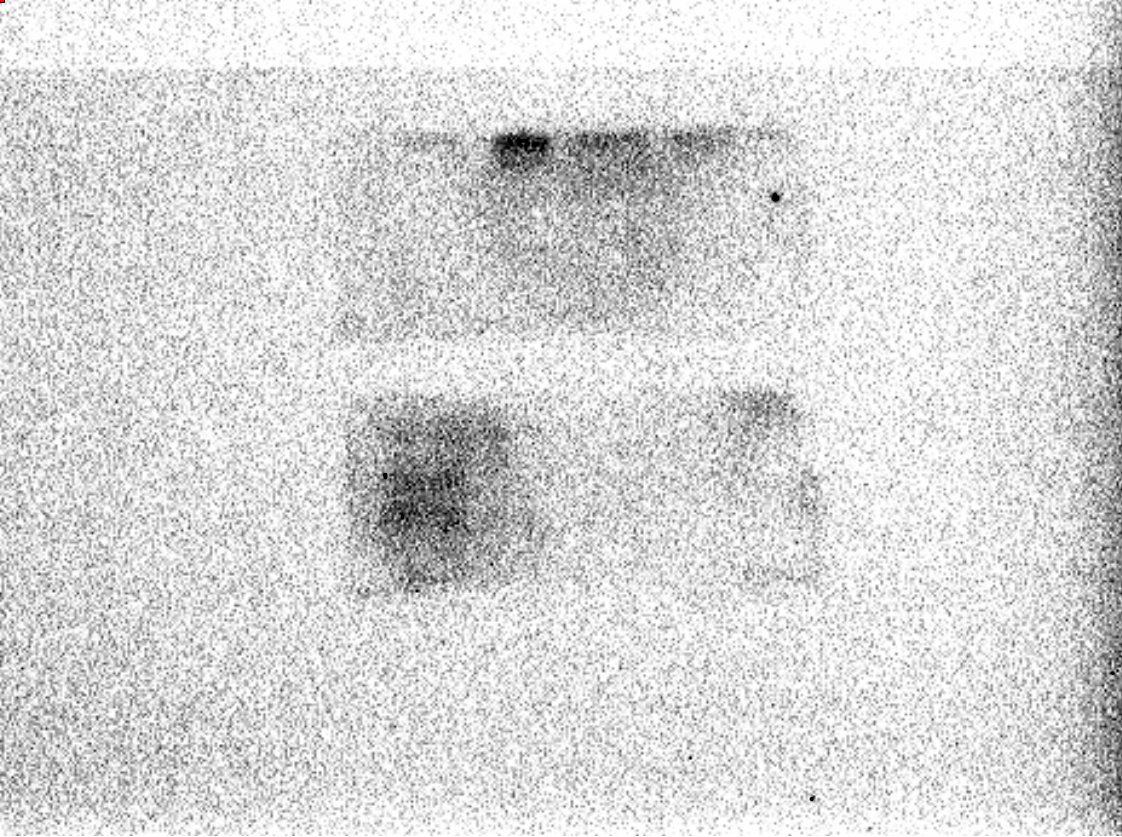

Supplement: Supplementary file 1 [file DataSheet1.zip › original WB images/72(p-JAK2-RAW).tif]

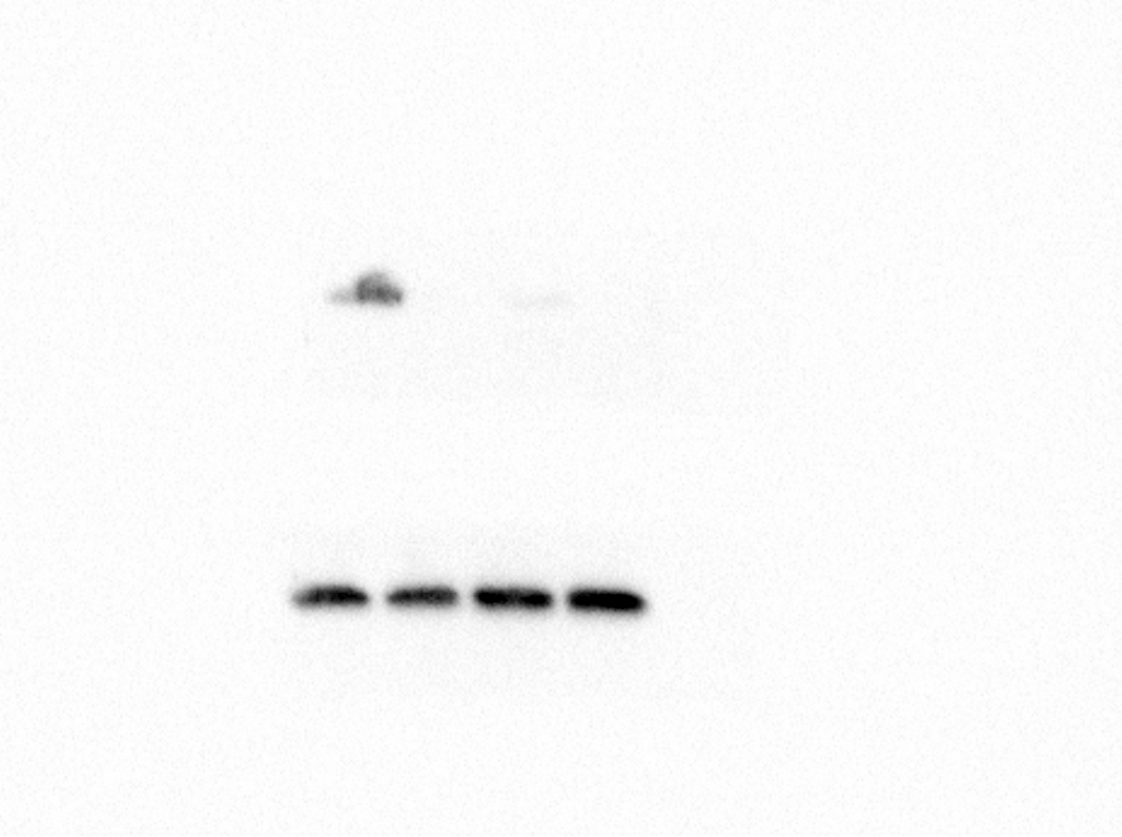

Supplement: Supplementary file 1 [file DataSheet1.zip › original WB images/73(GAPDH-RAW).tif]

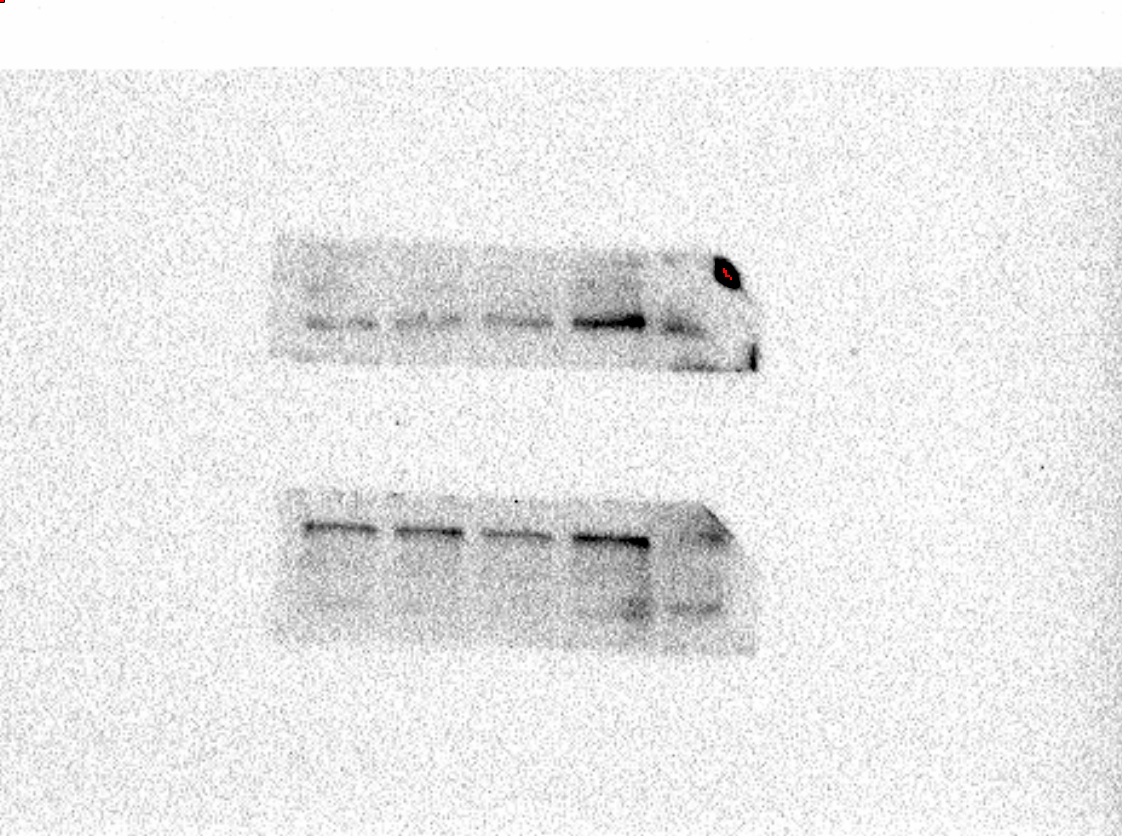

Supplement: Supplementary file 1 [file DataSheet1.zip › original WB images/76(GAPDH-IEC6).tif]

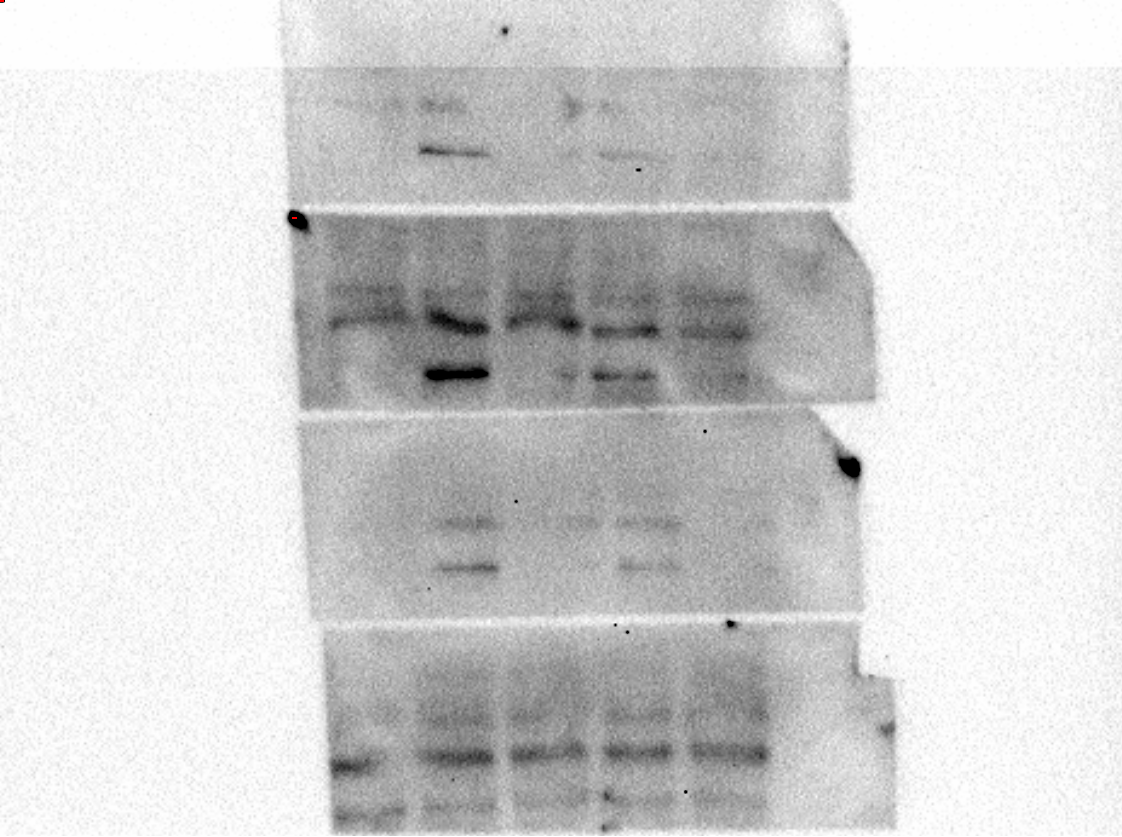

Supplement: Supplementary file 1 [file DataSheet1.zip › original WB images/79(p-JAK2) 80(GAPDH-pjak2).tif]

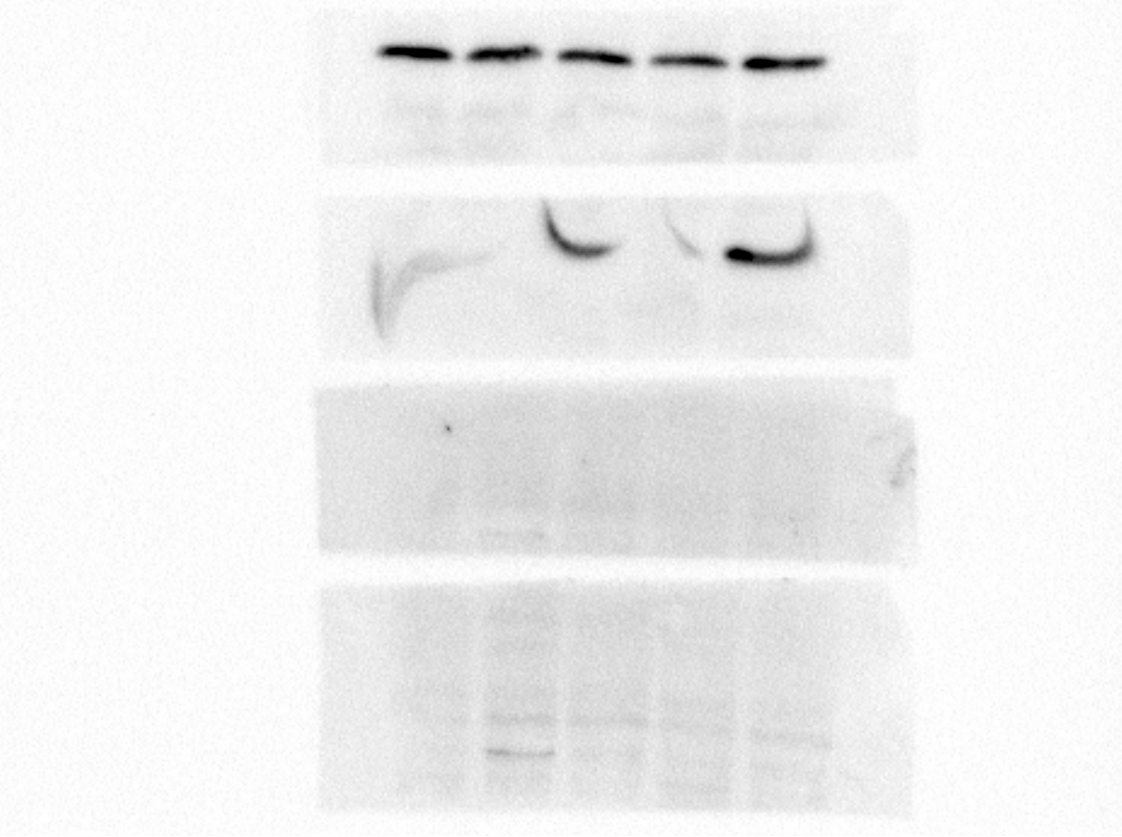

Supplement: Supplementary file 1 [file DataSheet1.zip › original WB images/8(GAPDH-MLCK).tif]

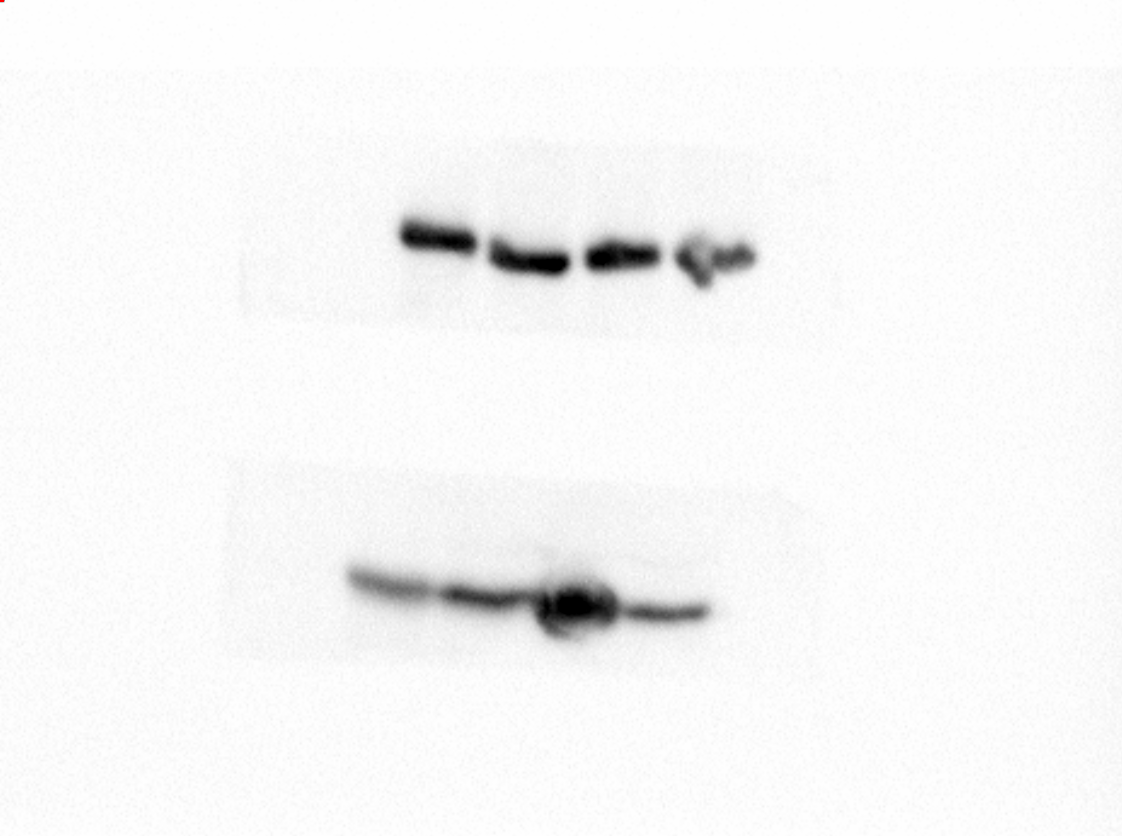

Supplement: Supplementary file 1 [file DataSheet1.zip › original WB images/84(GAPDH-pSTAT3).tif]

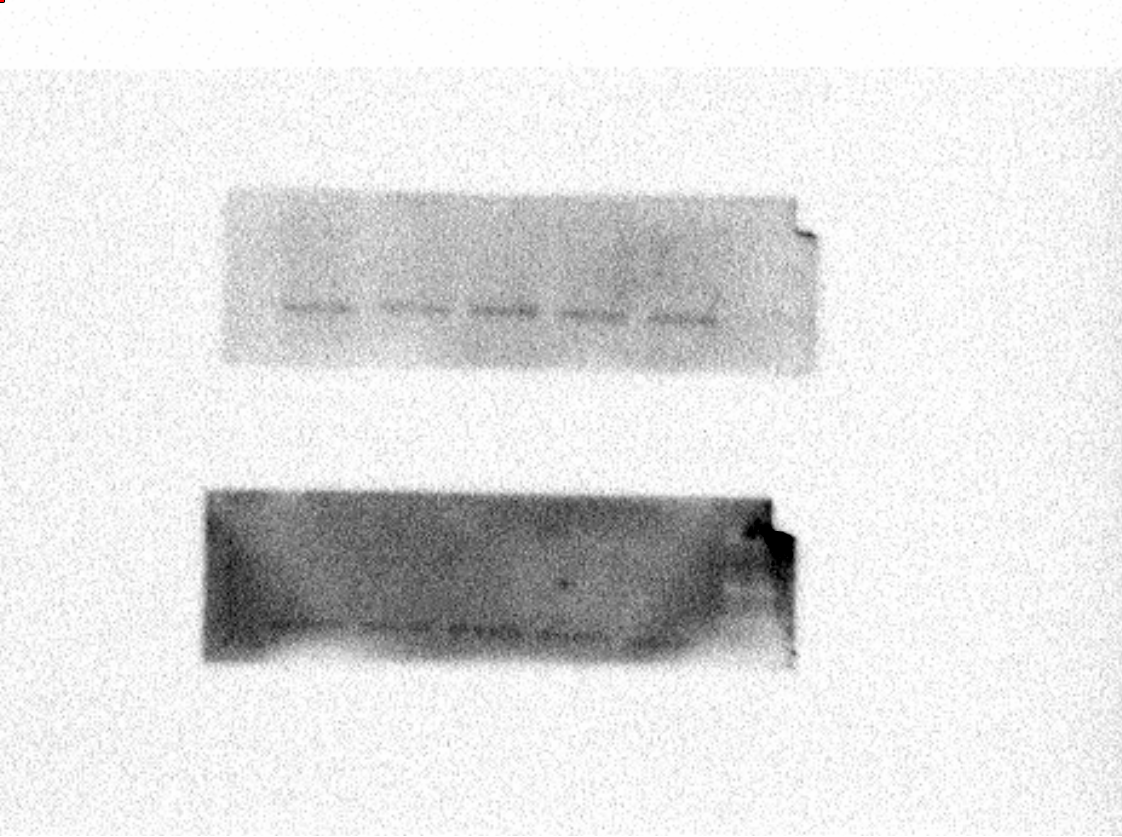

Supplement: Supplementary file 1 [file DataSheet1.zip › original WB images/9(JAK2) 66(GAPDH-JAK2).tif]

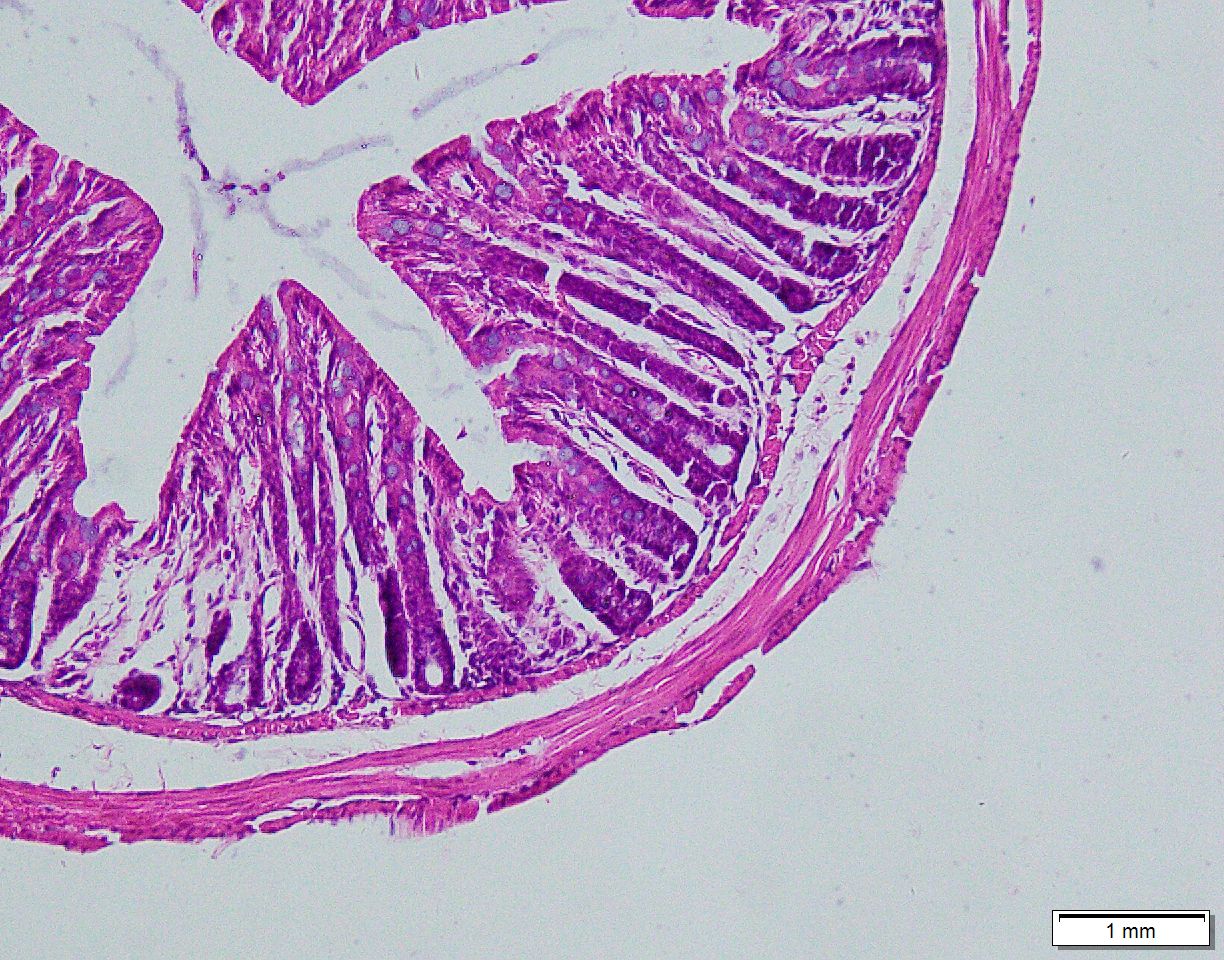

Supplement: Supplementary file 2 [file DataSheet2.zip › HE/fig1 100 100μm.JPG]

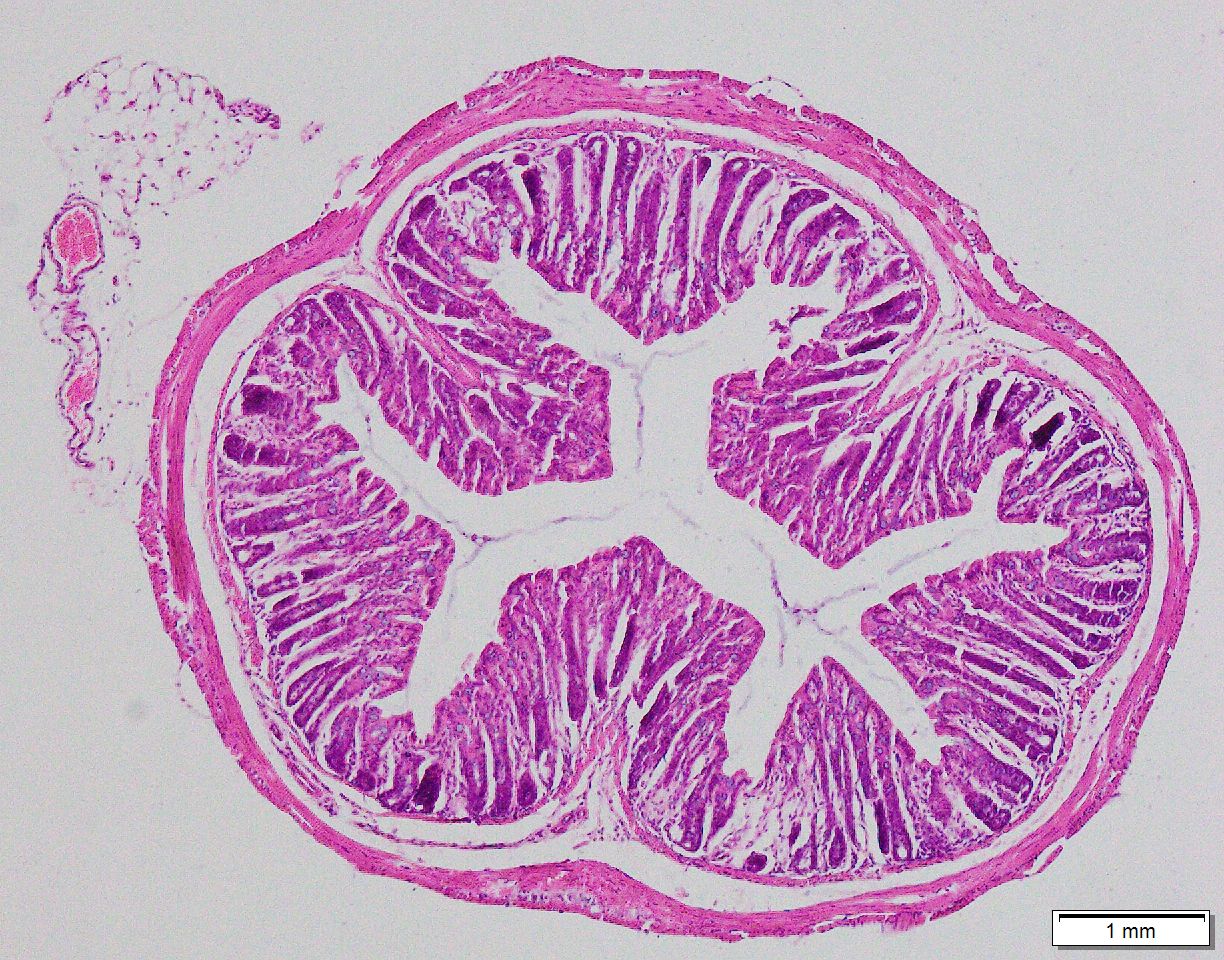

Supplement: Supplementary file 2 [file DataSheet2.zip › HE/fig1 100 200μm.JPG]

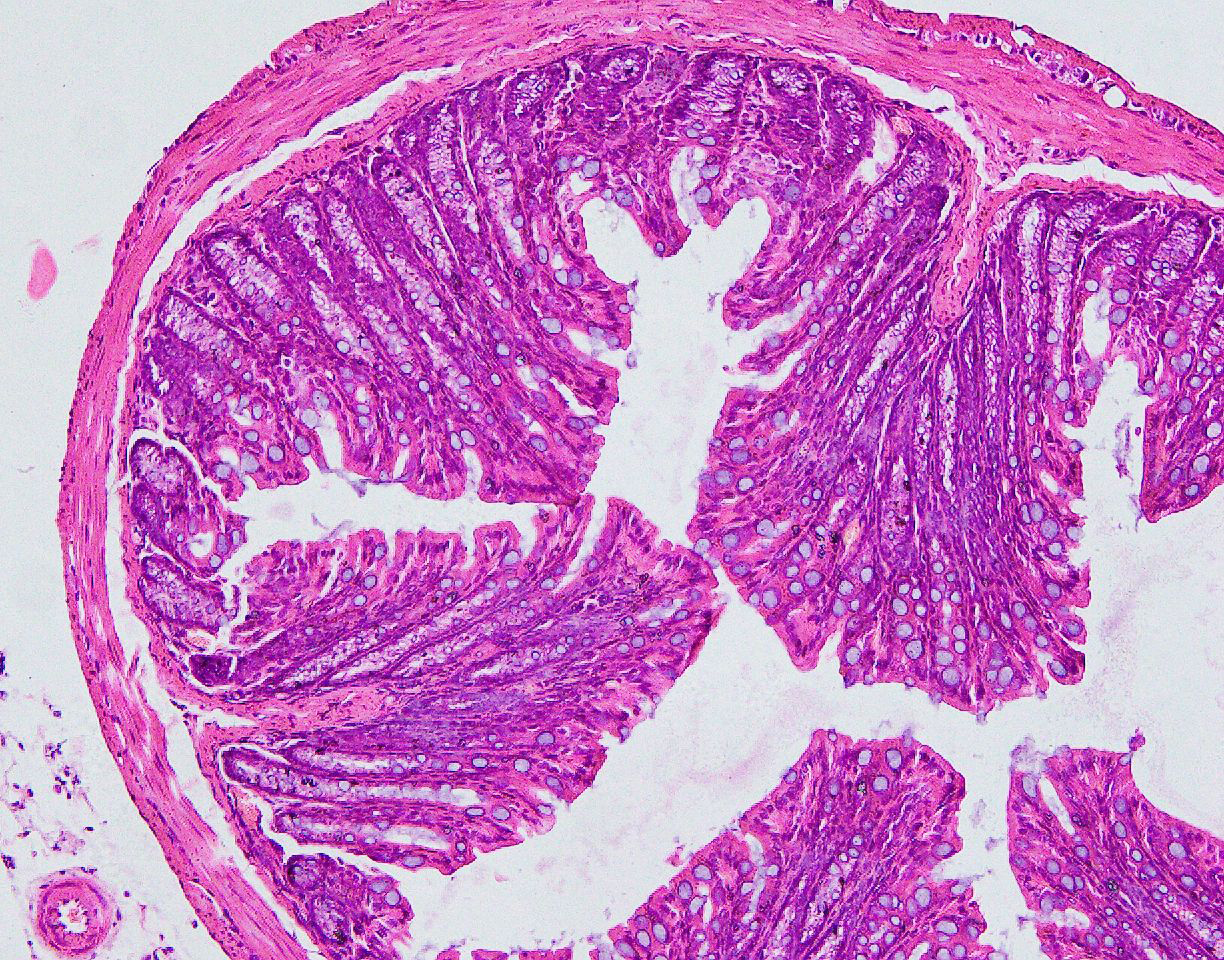

Supplement: Supplementary file 2 [file DataSheet2.zip › HE/fig1 50 100μm.JPG]

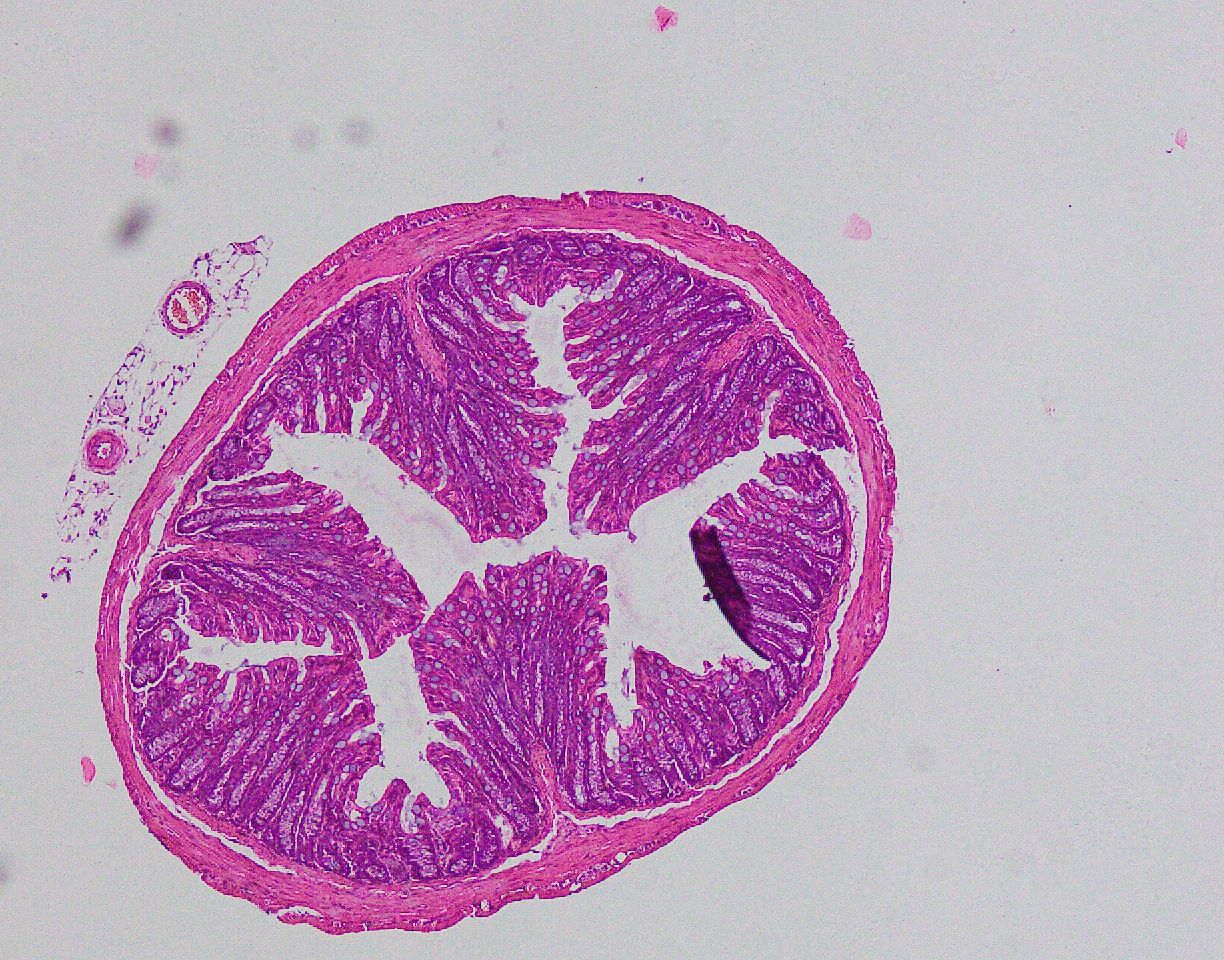

Supplement: Supplementary file 2 [file DataSheet2.zip › HE/fig1 50 200μm.JPG]

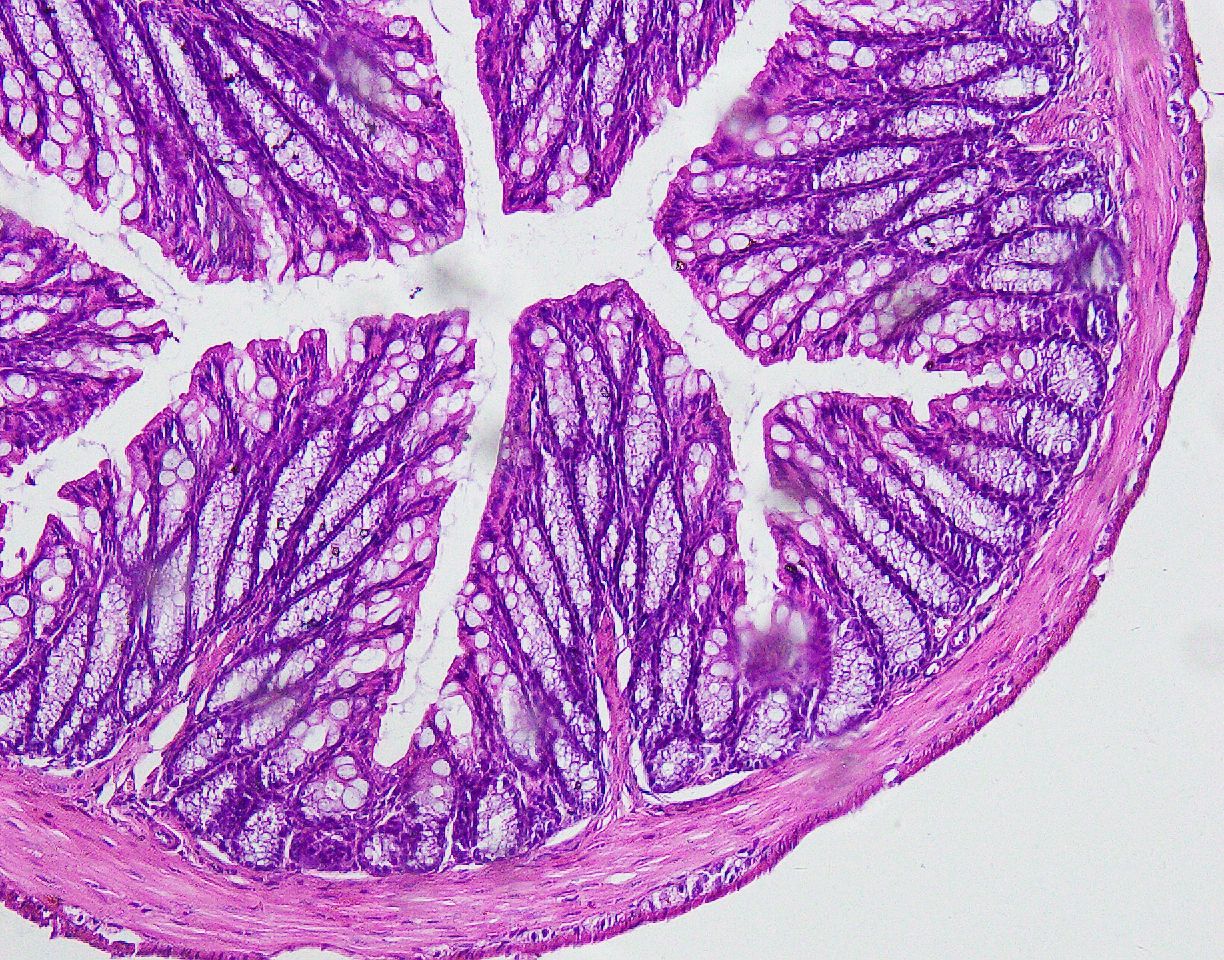

Supplement: Supplementary file 2 [file DataSheet2.zip › HE/fig1 Sham 100μm.JPG]

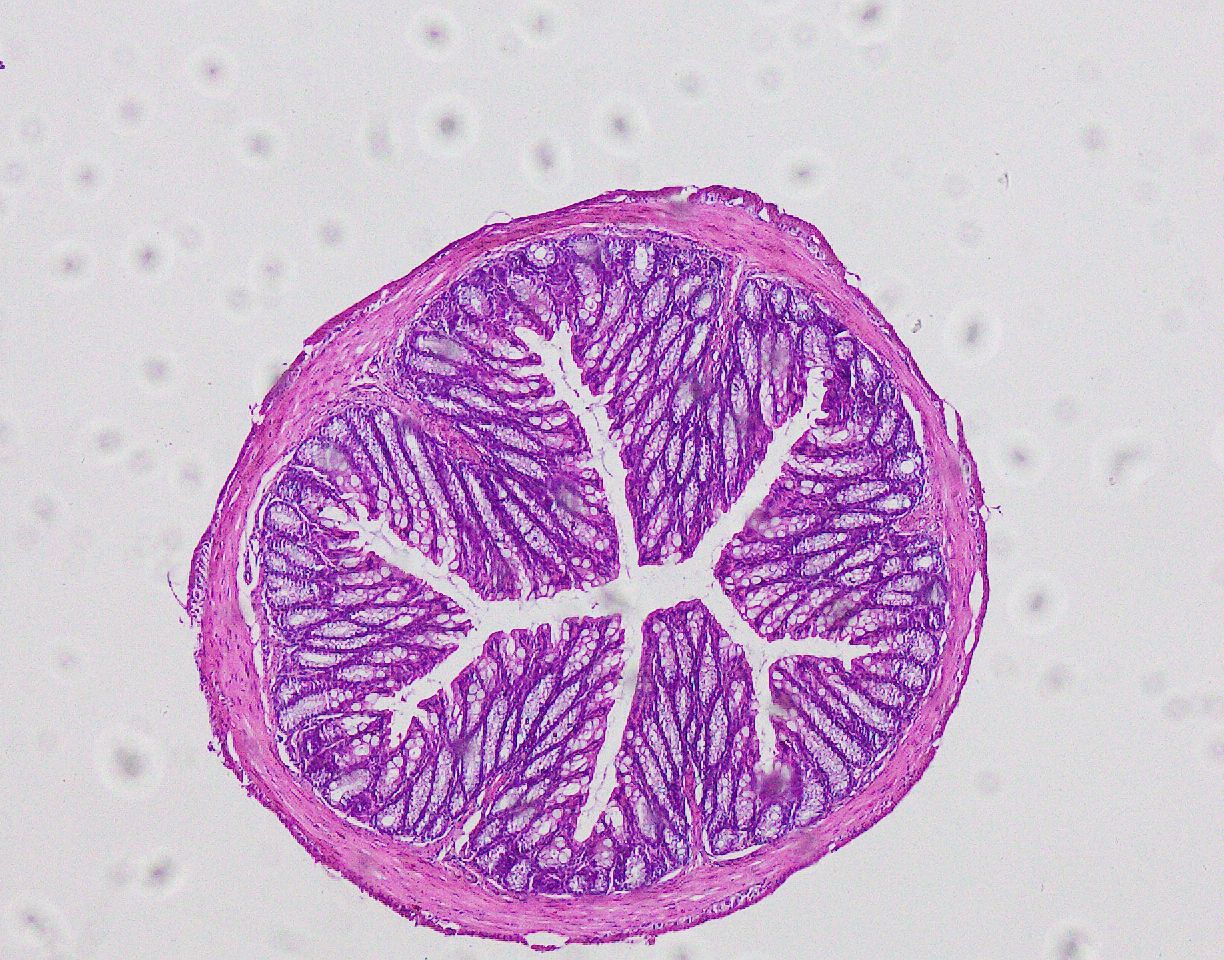

Supplement: Supplementary file 2 [file DataSheet2.zip › HE/fig1 Sham 200μm.JPG]

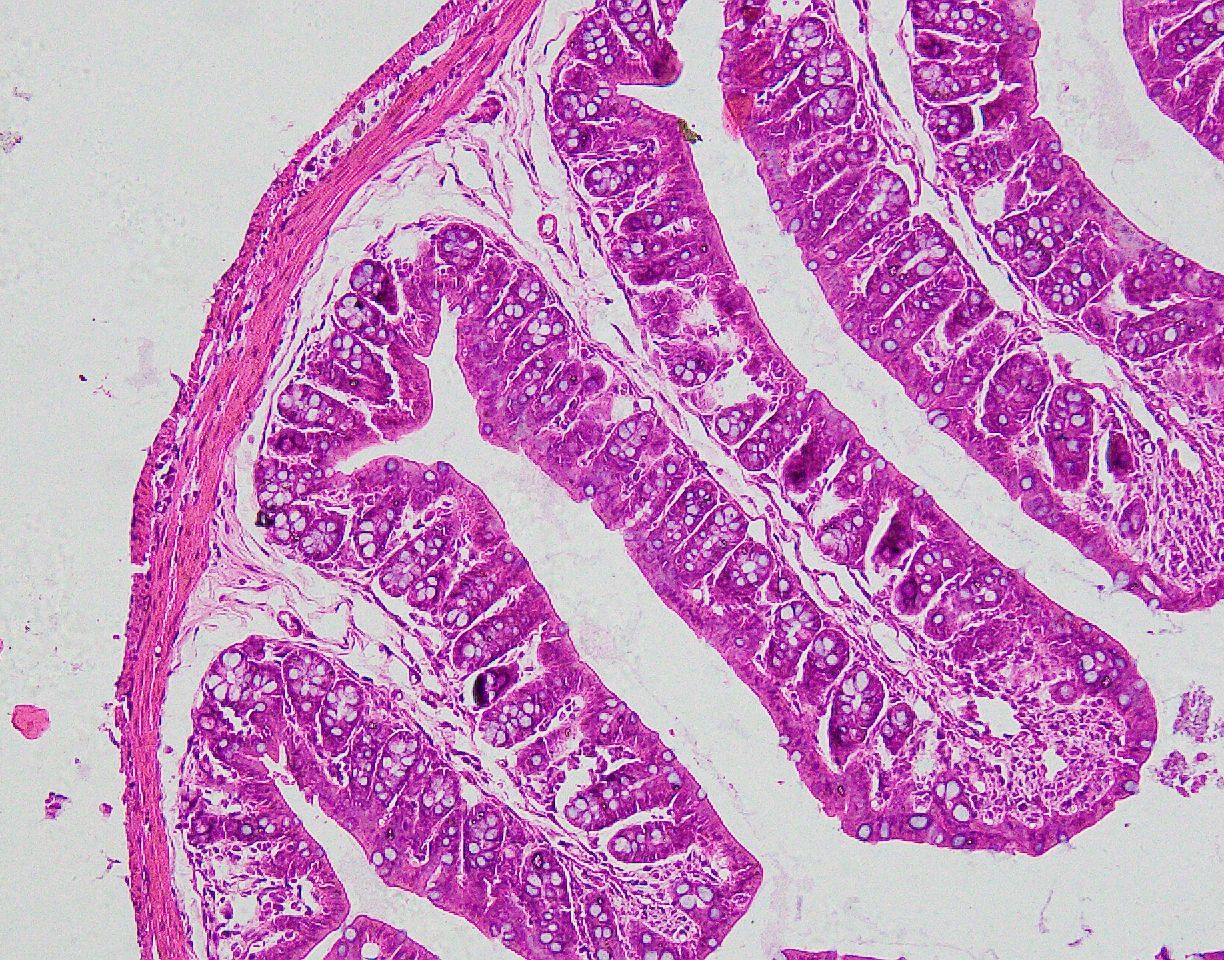

Supplement: Supplementary file 2 [file DataSheet2.zip › HE/fig2 100 100μm.JPG]

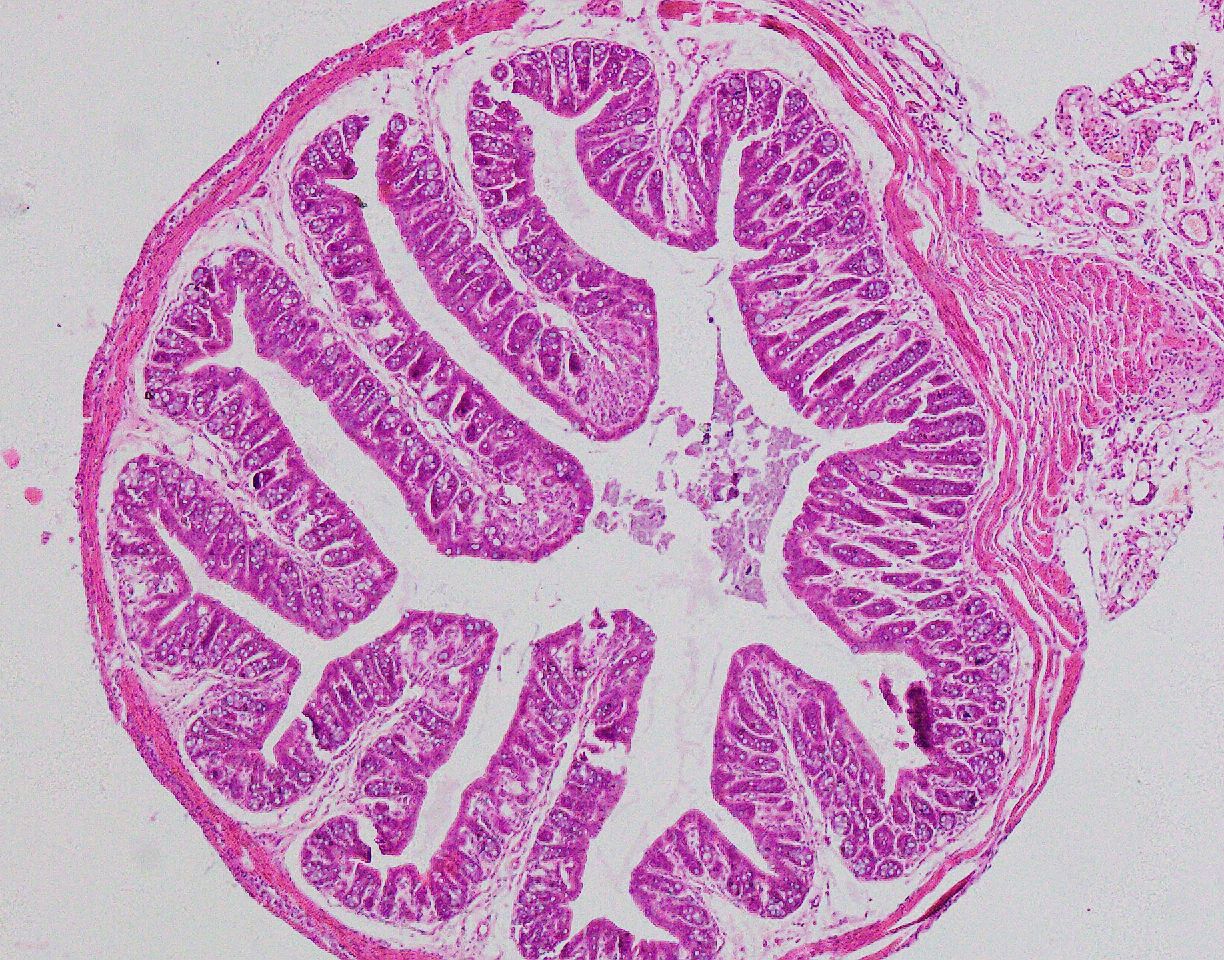

Supplement: Supplementary file 2 [file DataSheet2.zip › HE/fig2 100 200μm.JPG]

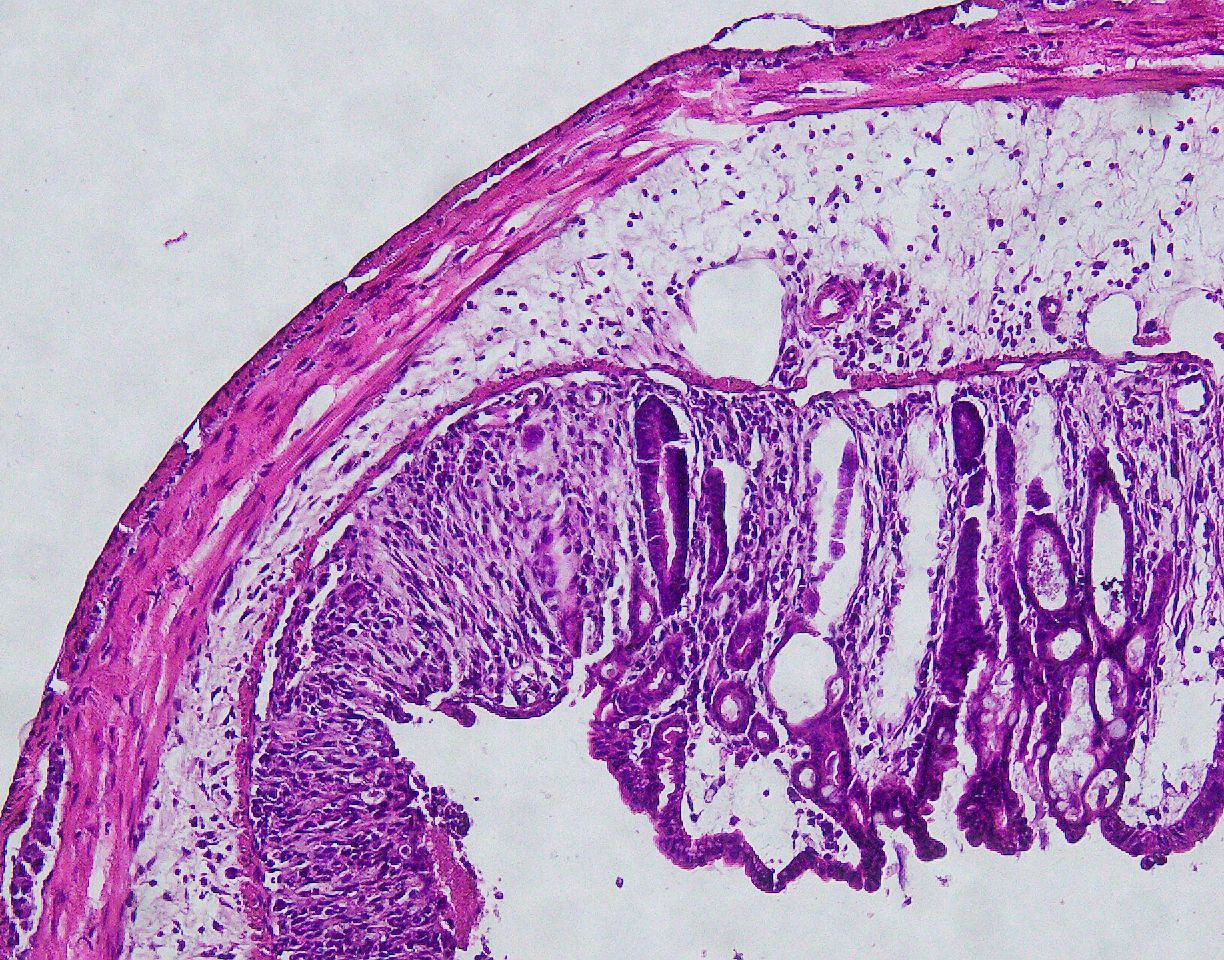

Supplement: Supplementary file 2 [file DataSheet2.zip › HE/fig2 50 100μm.JPG]

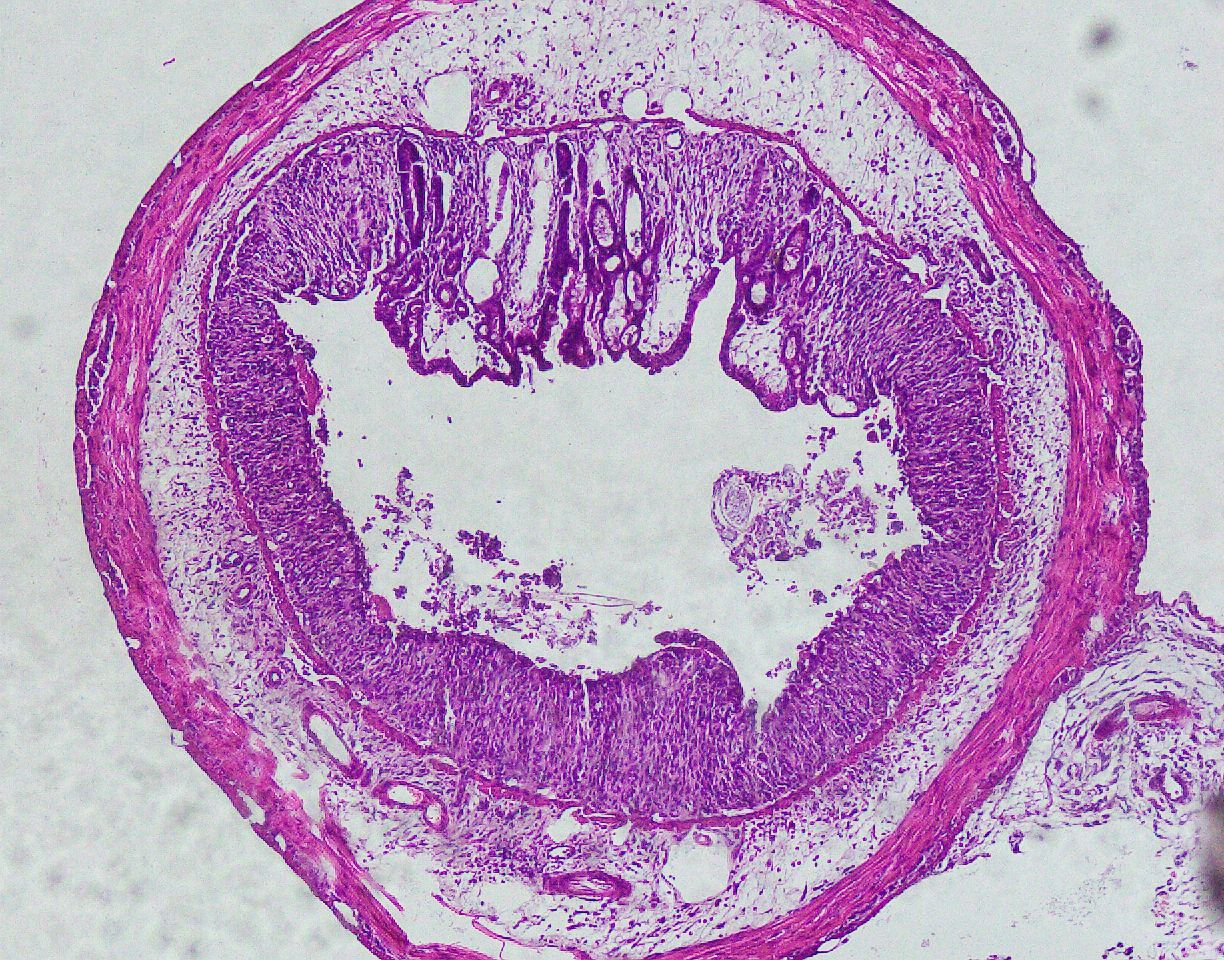

Supplement: Supplementary file 2 [file DataSheet2.zip › HE/fig2 50 200μm.JPG]

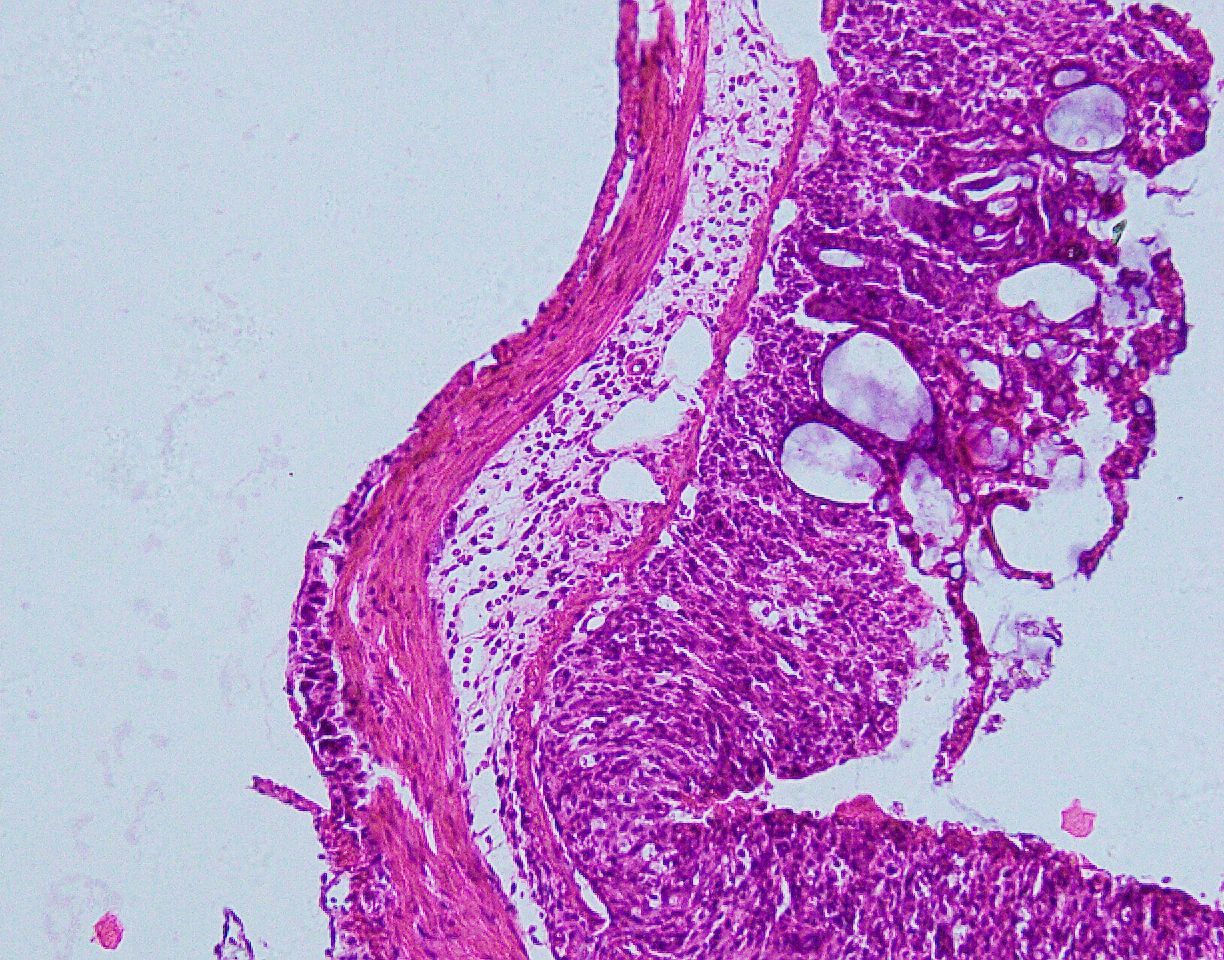

Supplement: Supplementary file 2 [file DataSheet2.zip › HE/fig2 Control 100μm.JPG]

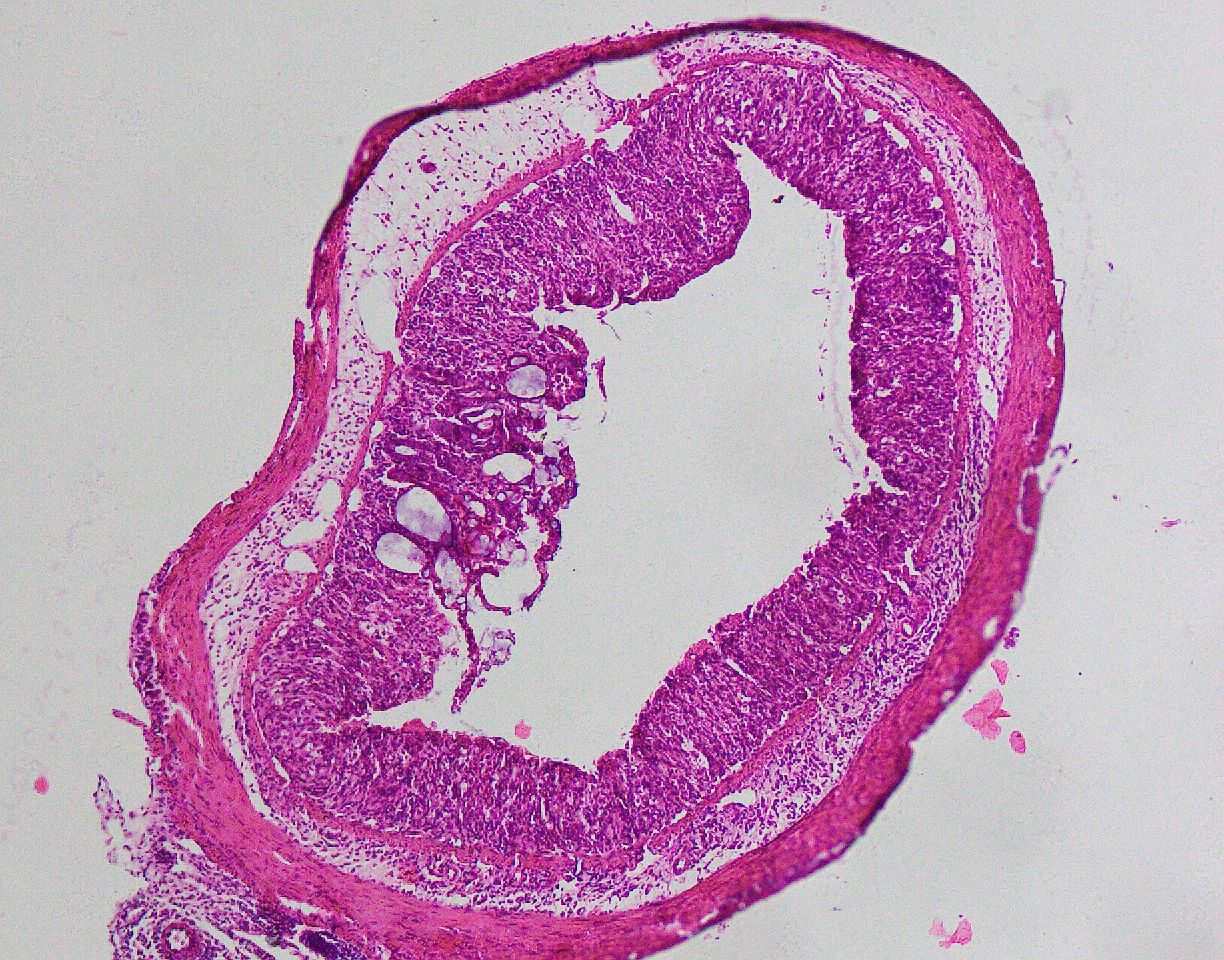

Supplement: Supplementary file 2 [file DataSheet2.zip › HE/fig2 Control 200μm.JPG]

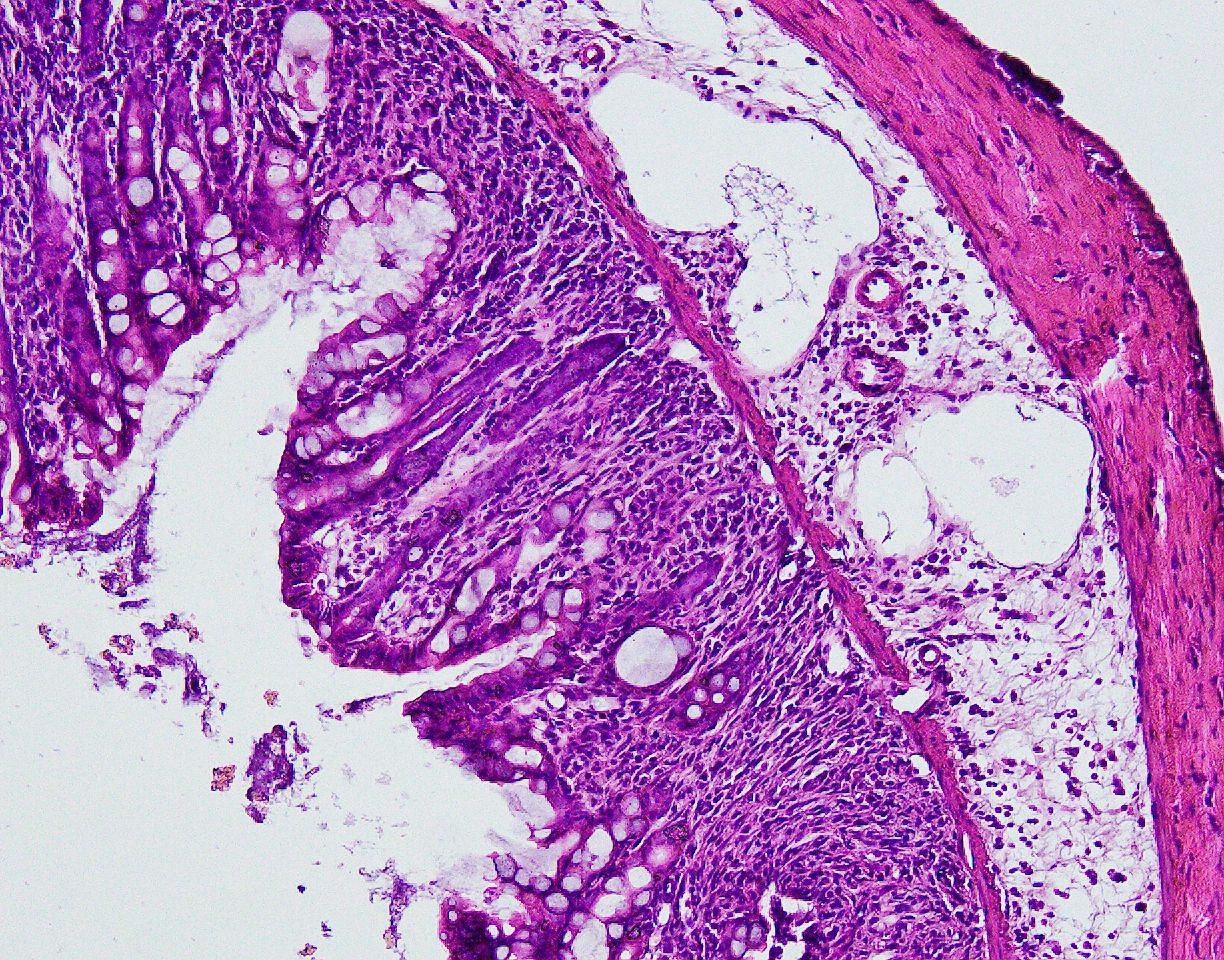

Supplement: Supplementary file 2 [file DataSheet2.zip › HE/fig2 SASP 100μm.JPG]

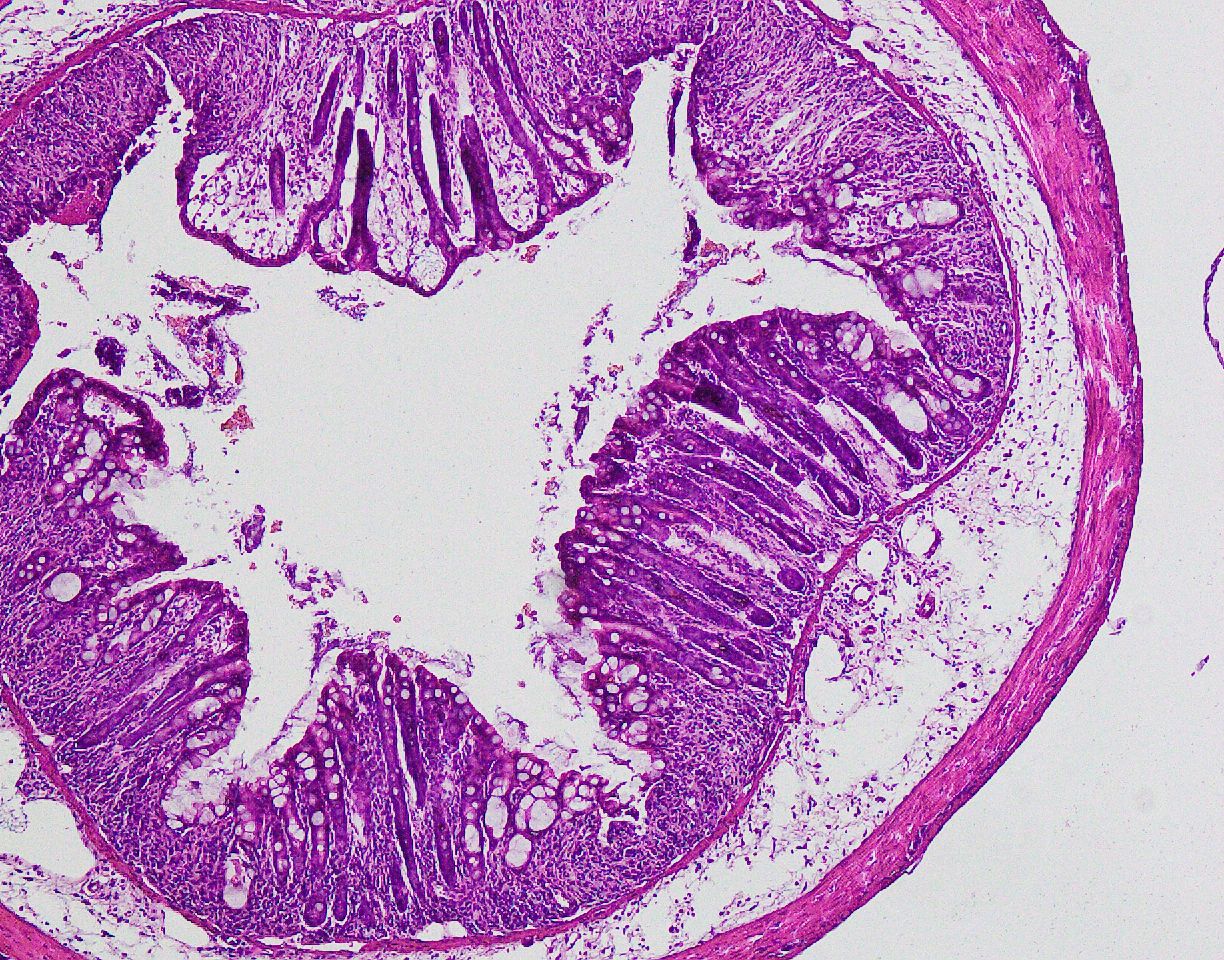

Supplement: Supplementary file 2 [file DataSheet2.zip › HE/fig2 SASP 200μm.JPG]

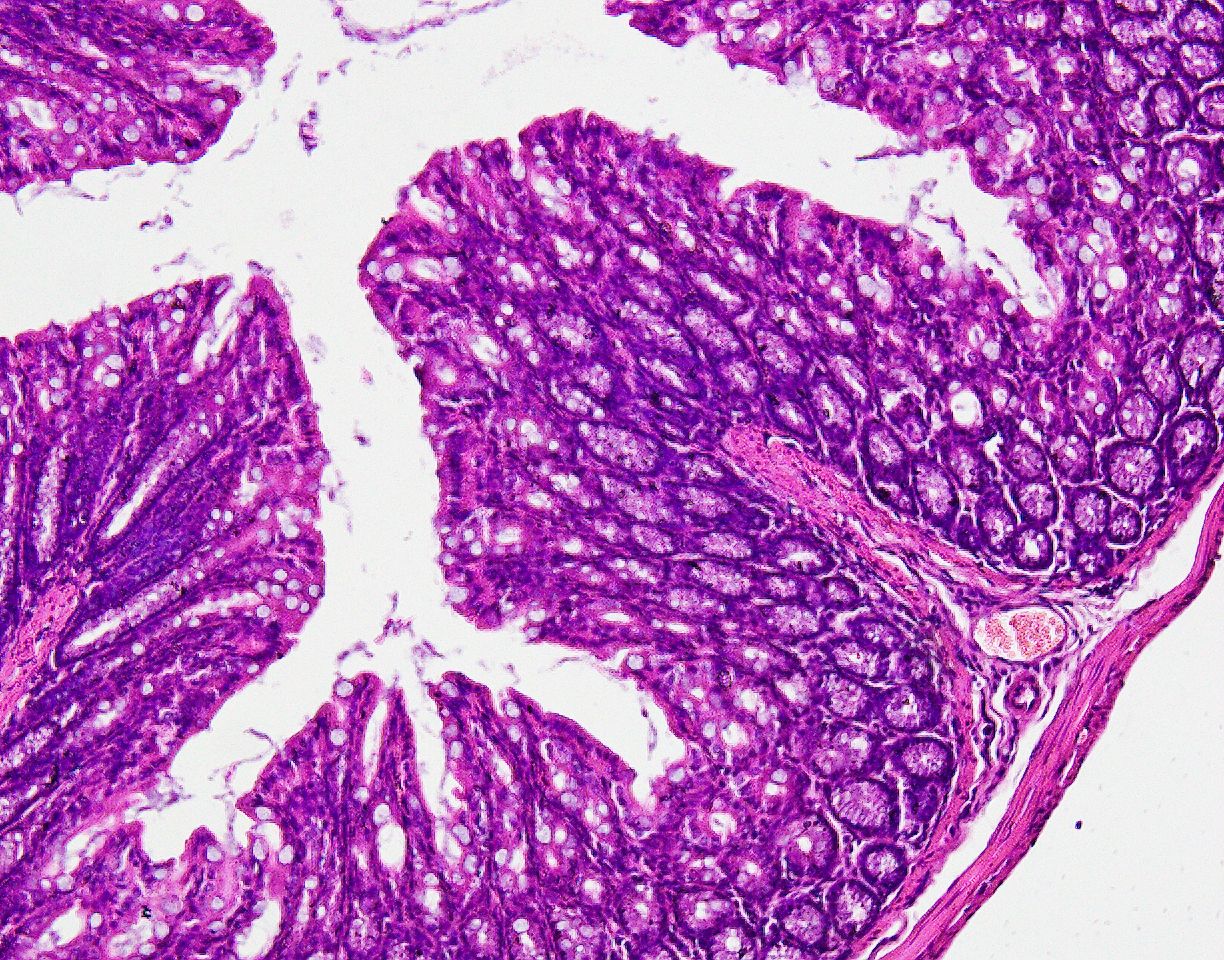

Supplement: Supplementary file 2 [file DataSheet2.zip › HE/fig2 Sham 100μm.JPG]

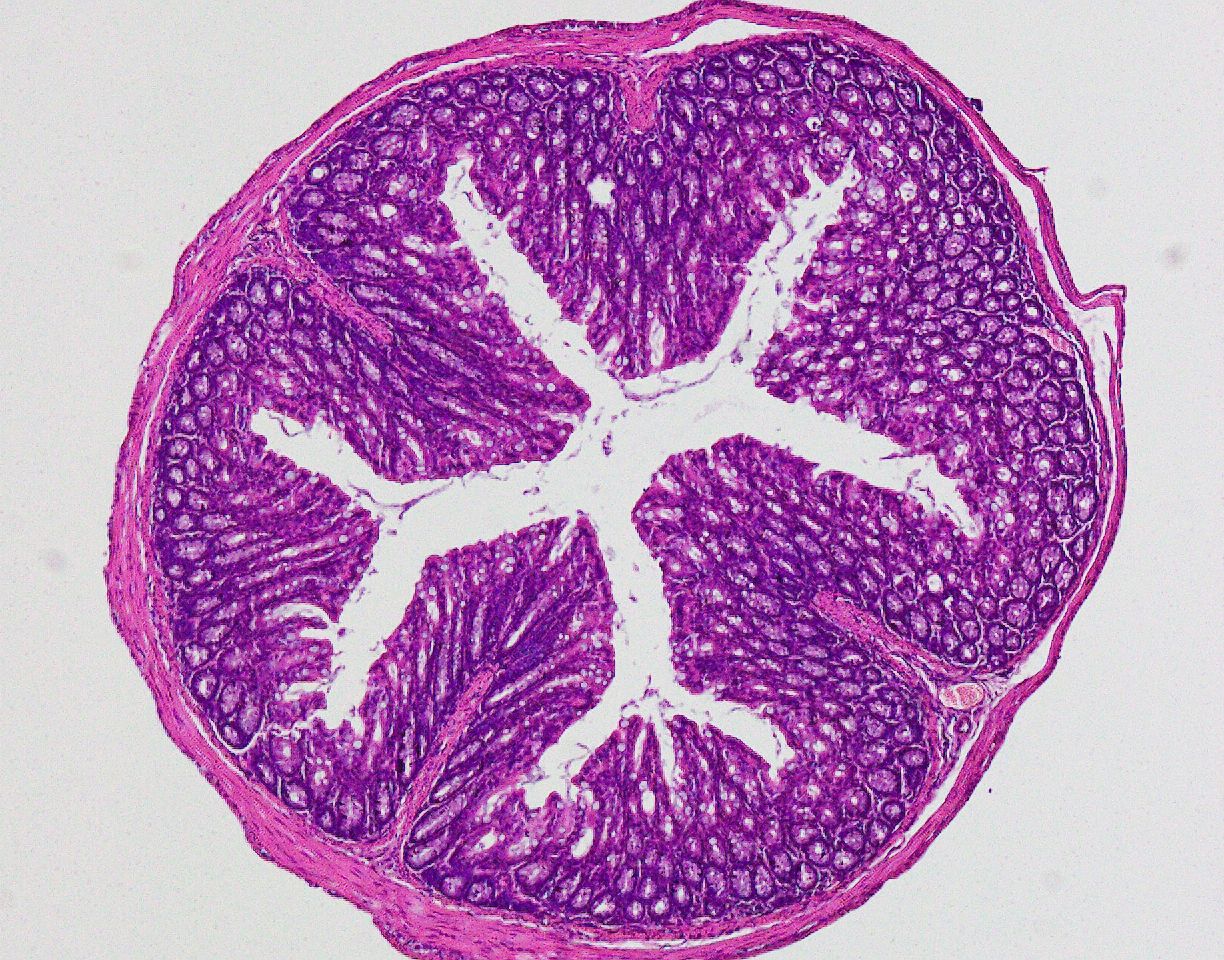

Supplement: Supplementary file 2 [file DataSheet2.zip › HE/fig2 Sham 200μm.JPG]

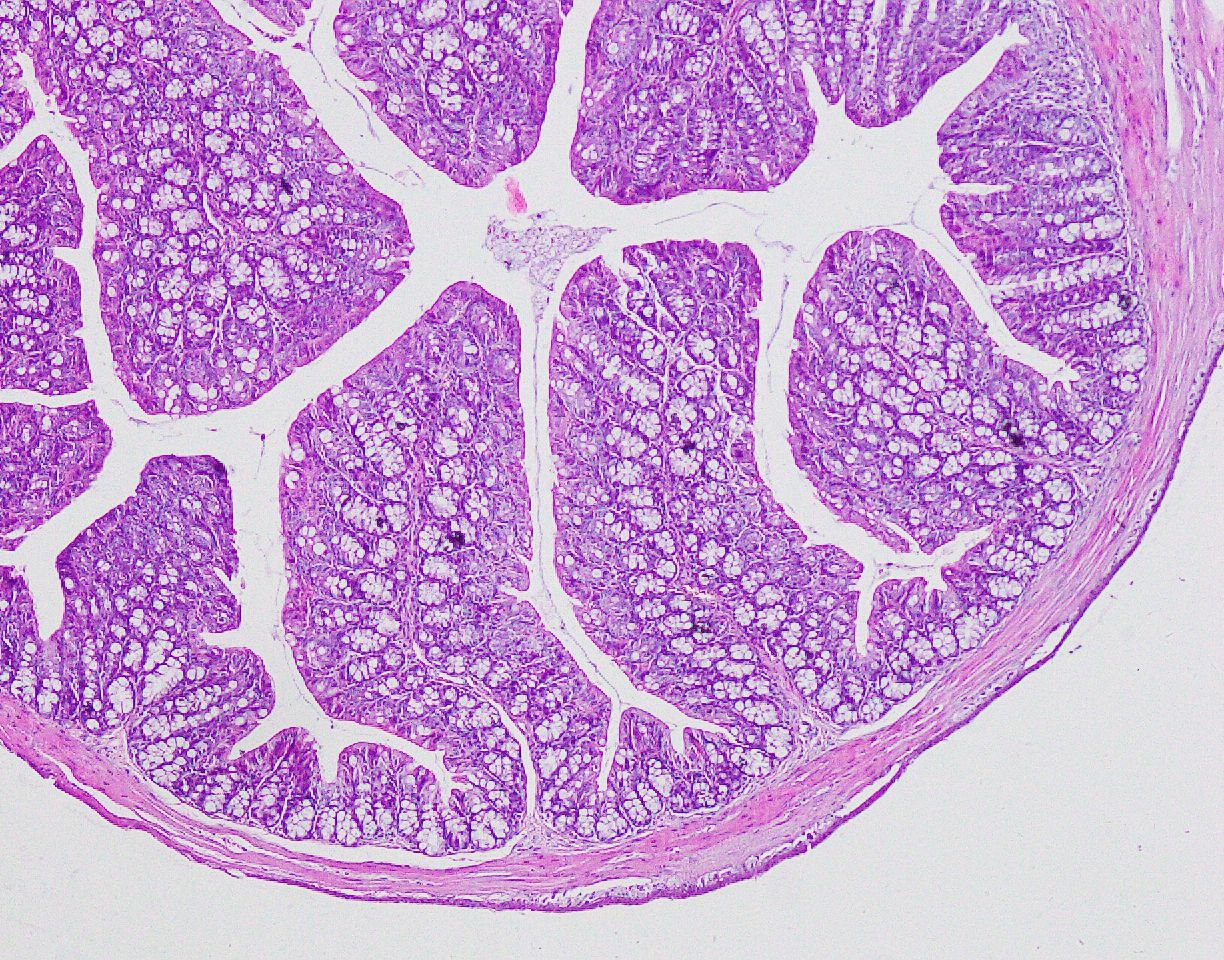

Supplement: Supplementary file 2 [file DataSheet2.zip › HE/fig7 Ag490 100μm.JPG]

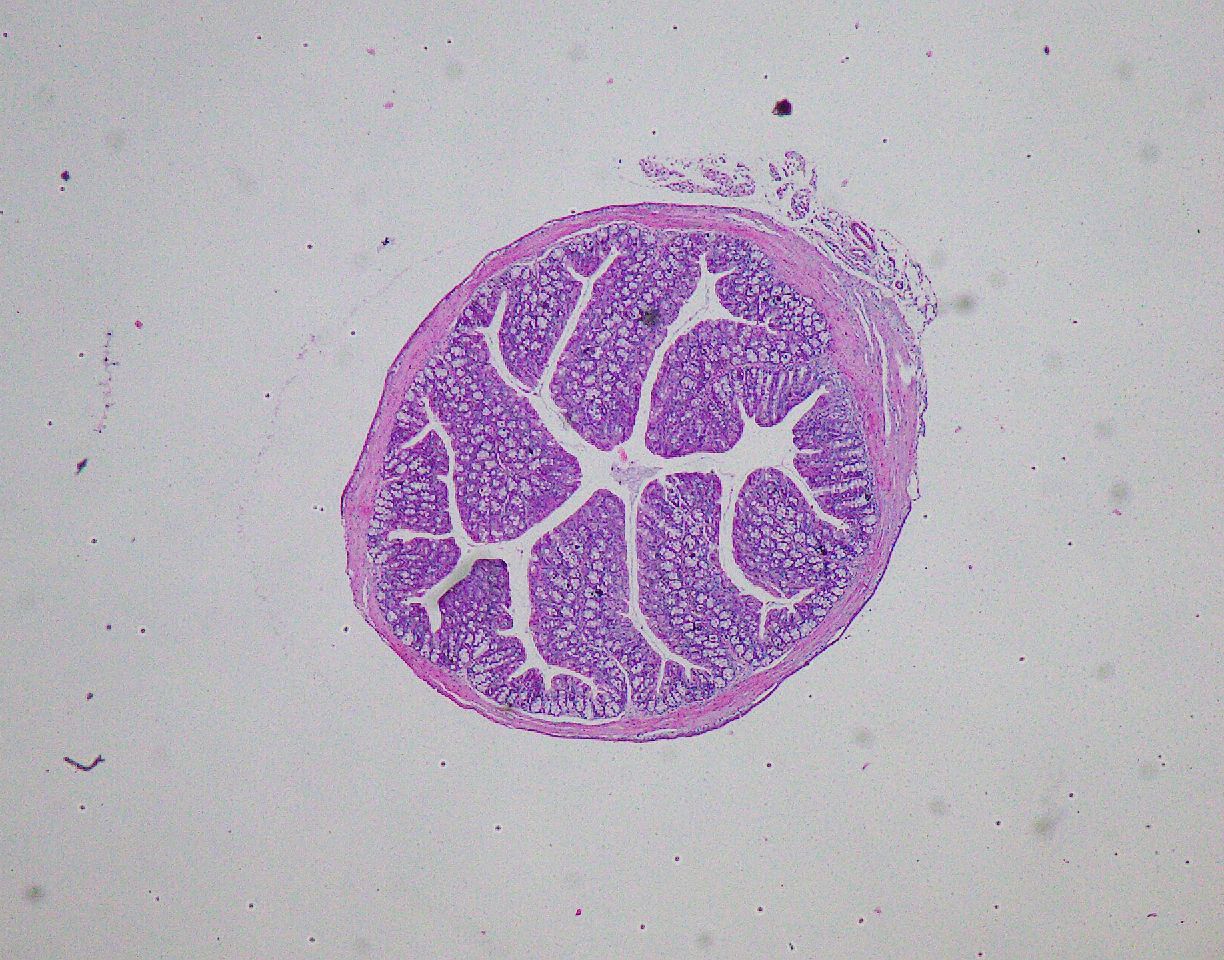

Supplement: Supplementary file 2 [file DataSheet2.zip › HE/fig7 Ag490 200μm.JPG]

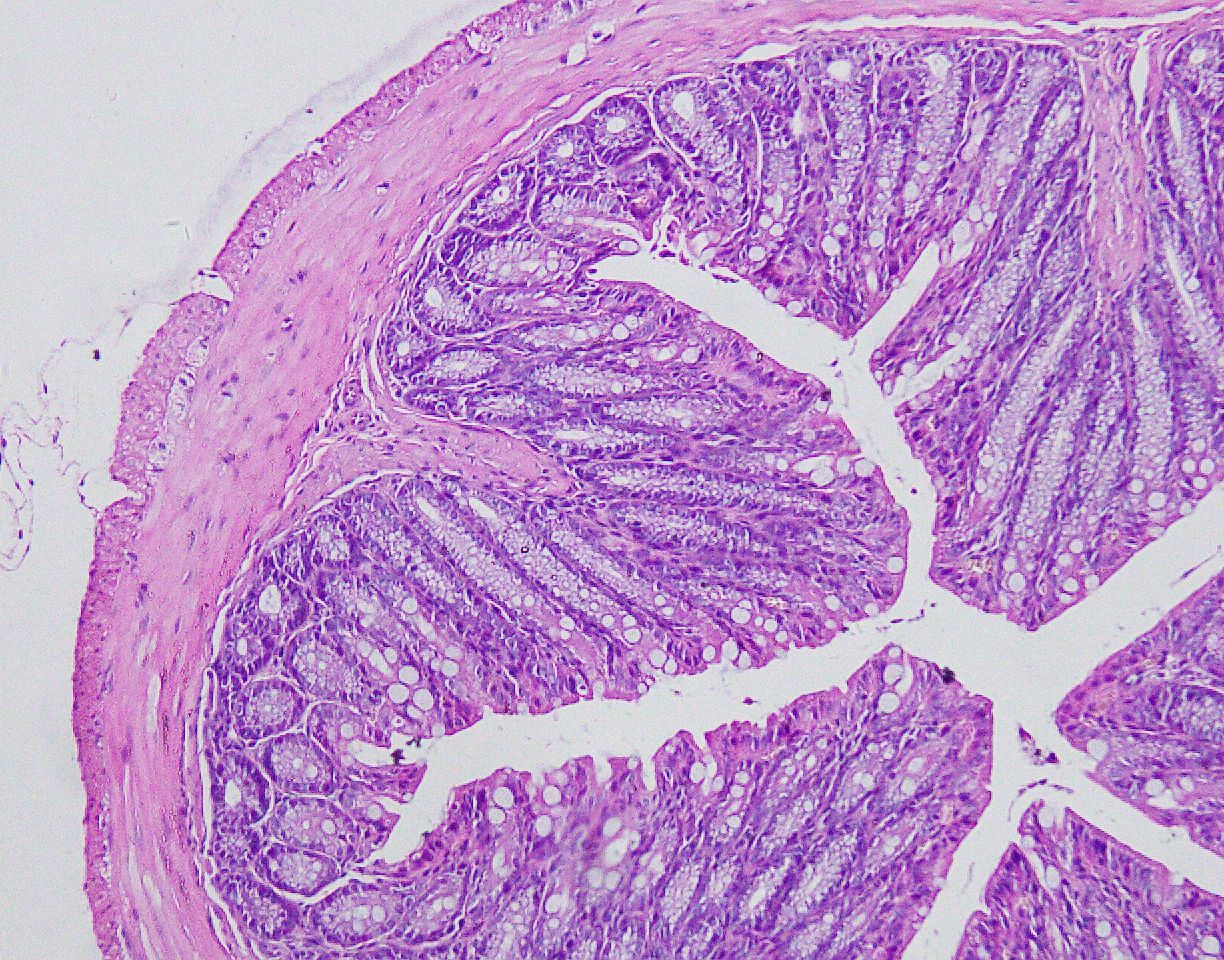

Supplement: Supplementary file 2 [file DataSheet2.zip › HE/fig7 Arbutin 100μm.JPG]

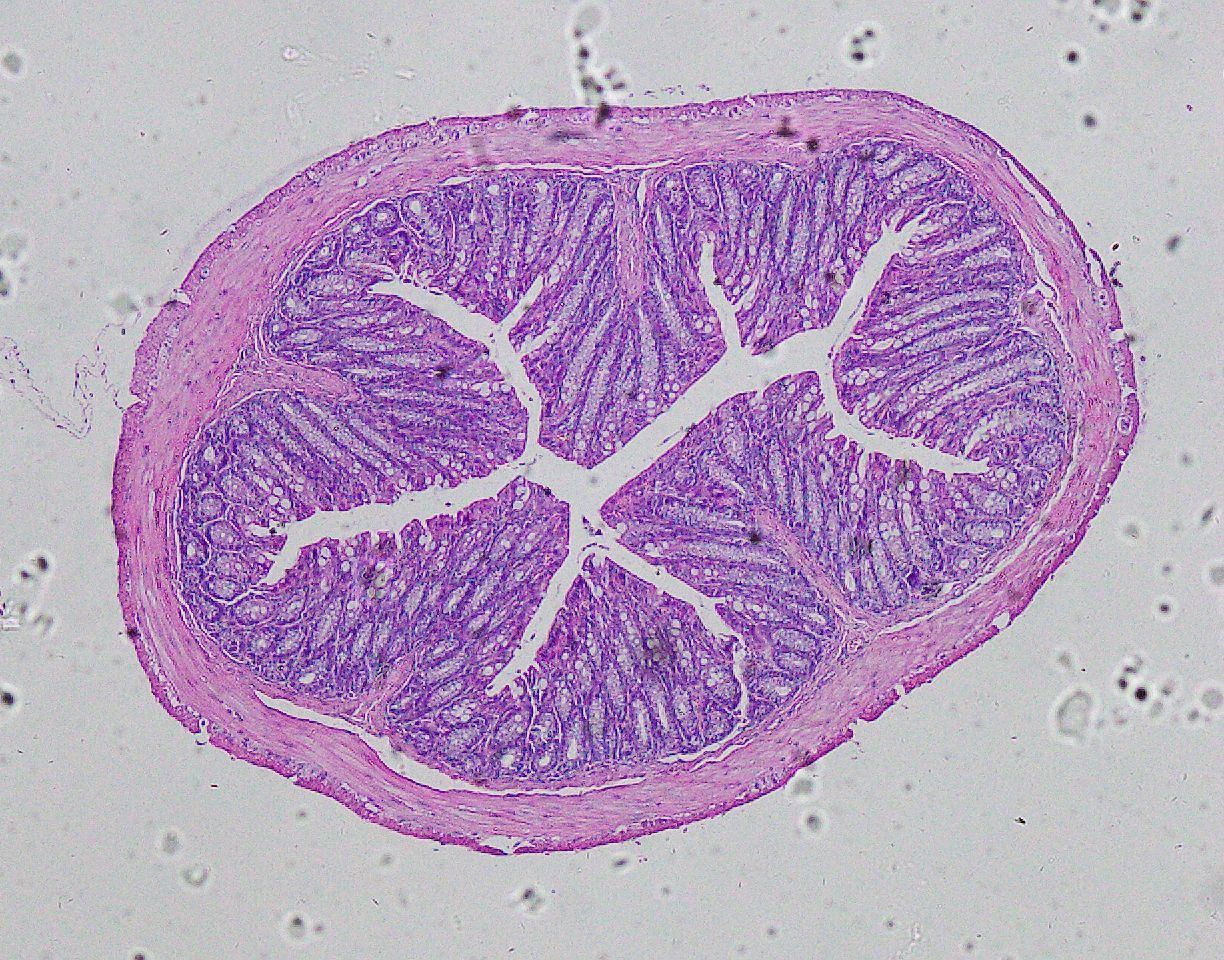

Supplement: Supplementary file 2 [file DataSheet2.zip › HE/fig7 Arbutin 200μm.JPG]

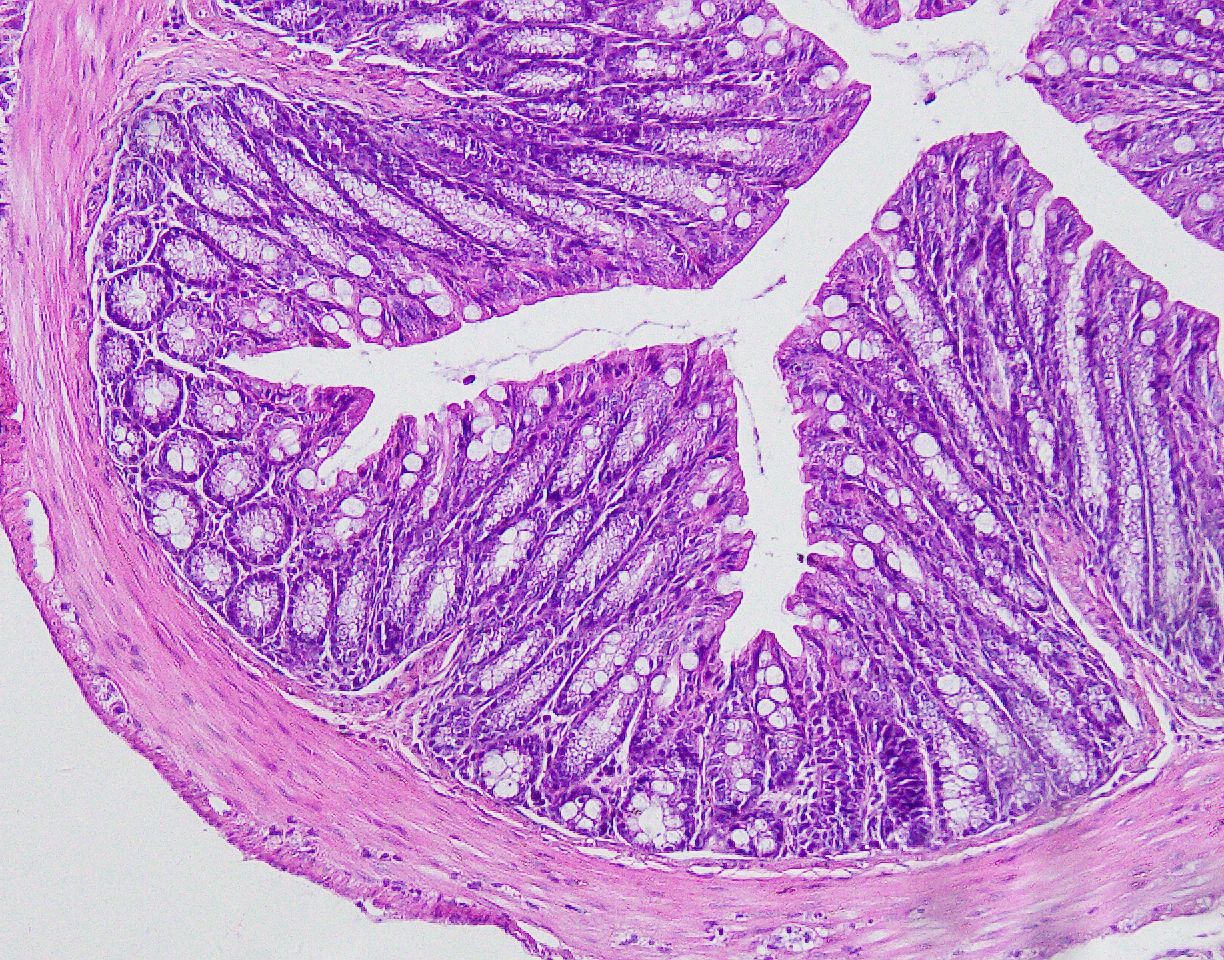

Supplement: Supplementary file 2 [file DataSheet2.zip › HE/fig7 Arbutin+Ag490 100μm.JPG]

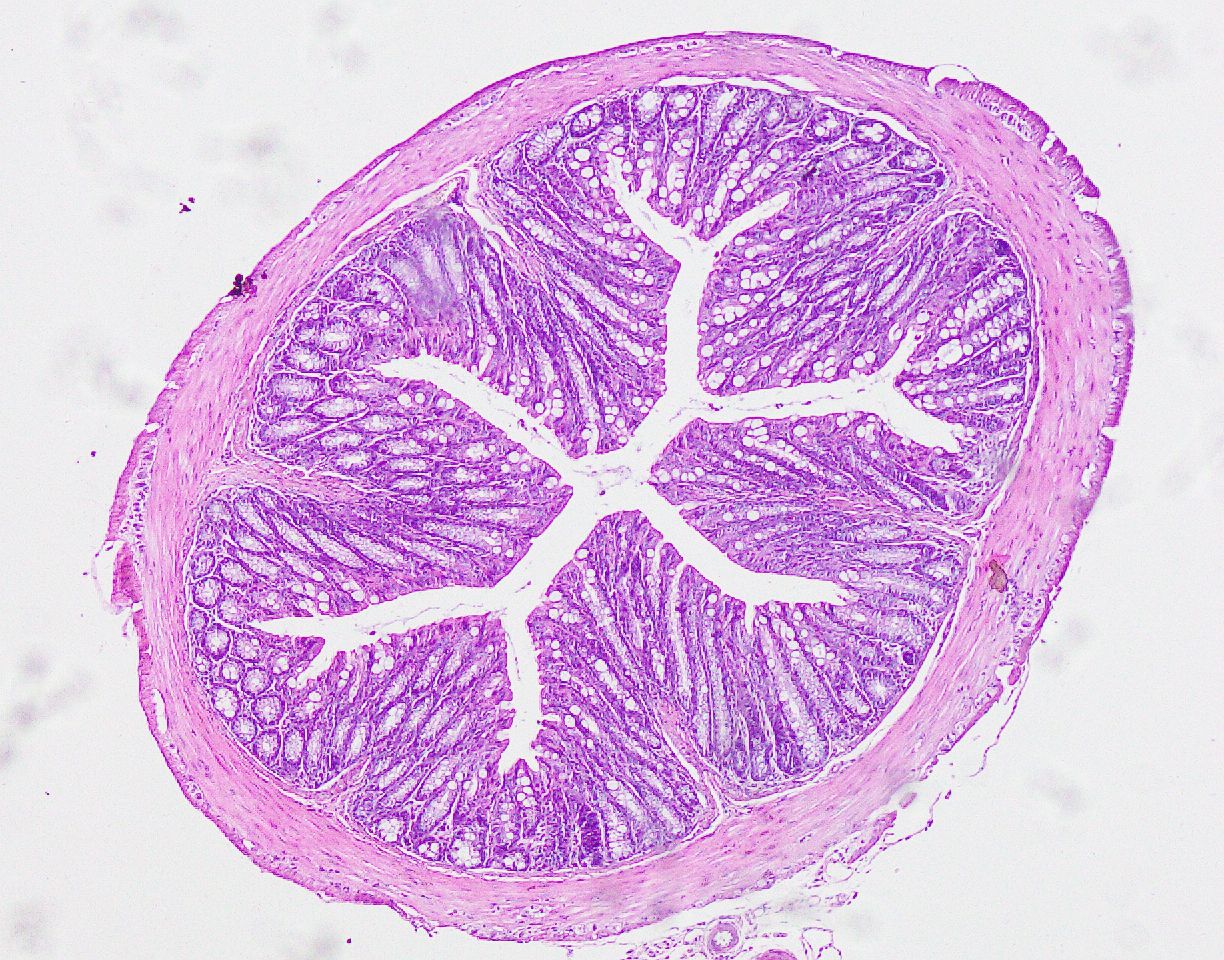

Supplement: Supplementary file 2 [file DataSheet2.zip › HE/fig7 Arbutin+Ag490 200μm.JPG]

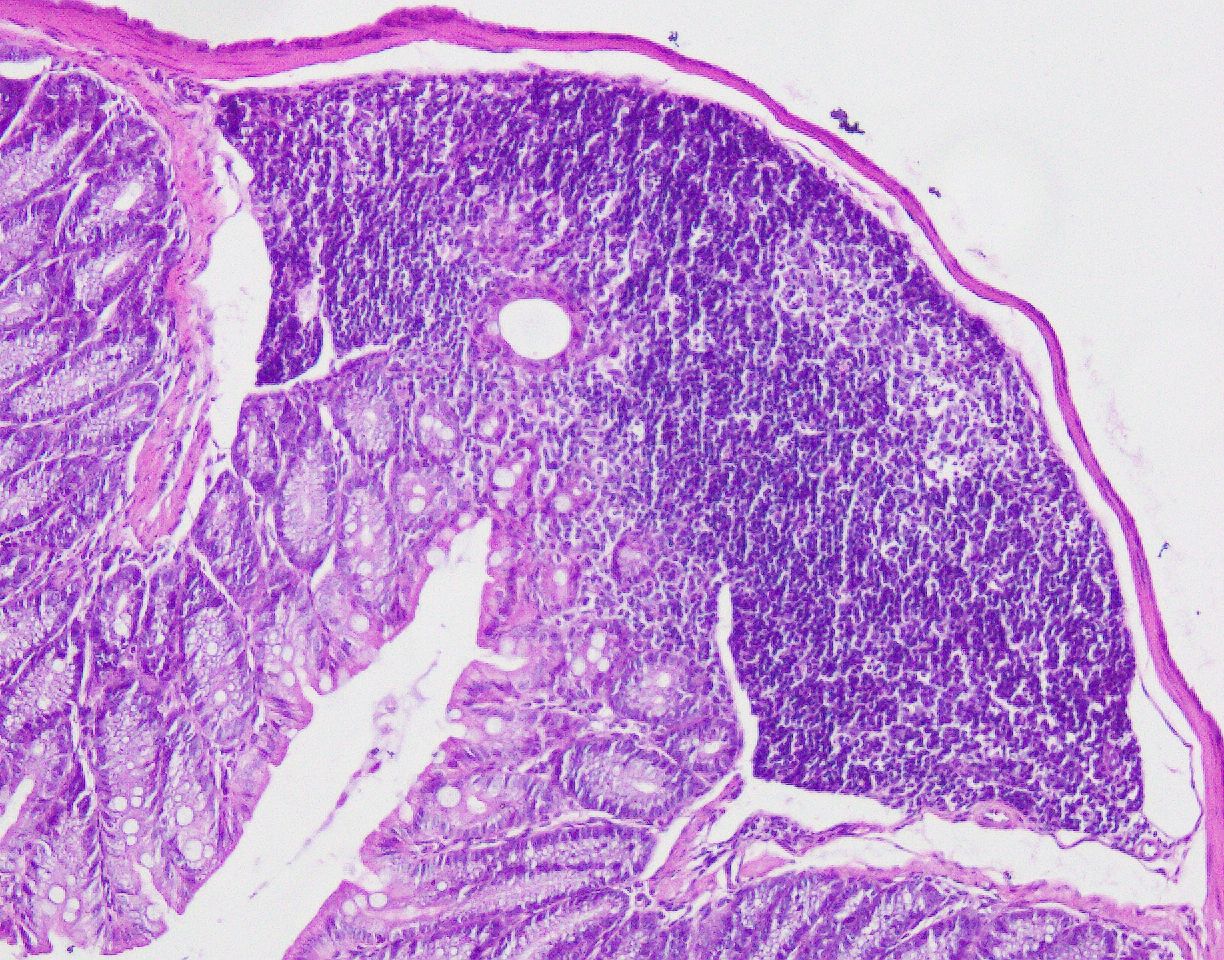

Supplement: Supplementary file 2 [file DataSheet2.zip › HE/fig7 Control 100μm.JPG]

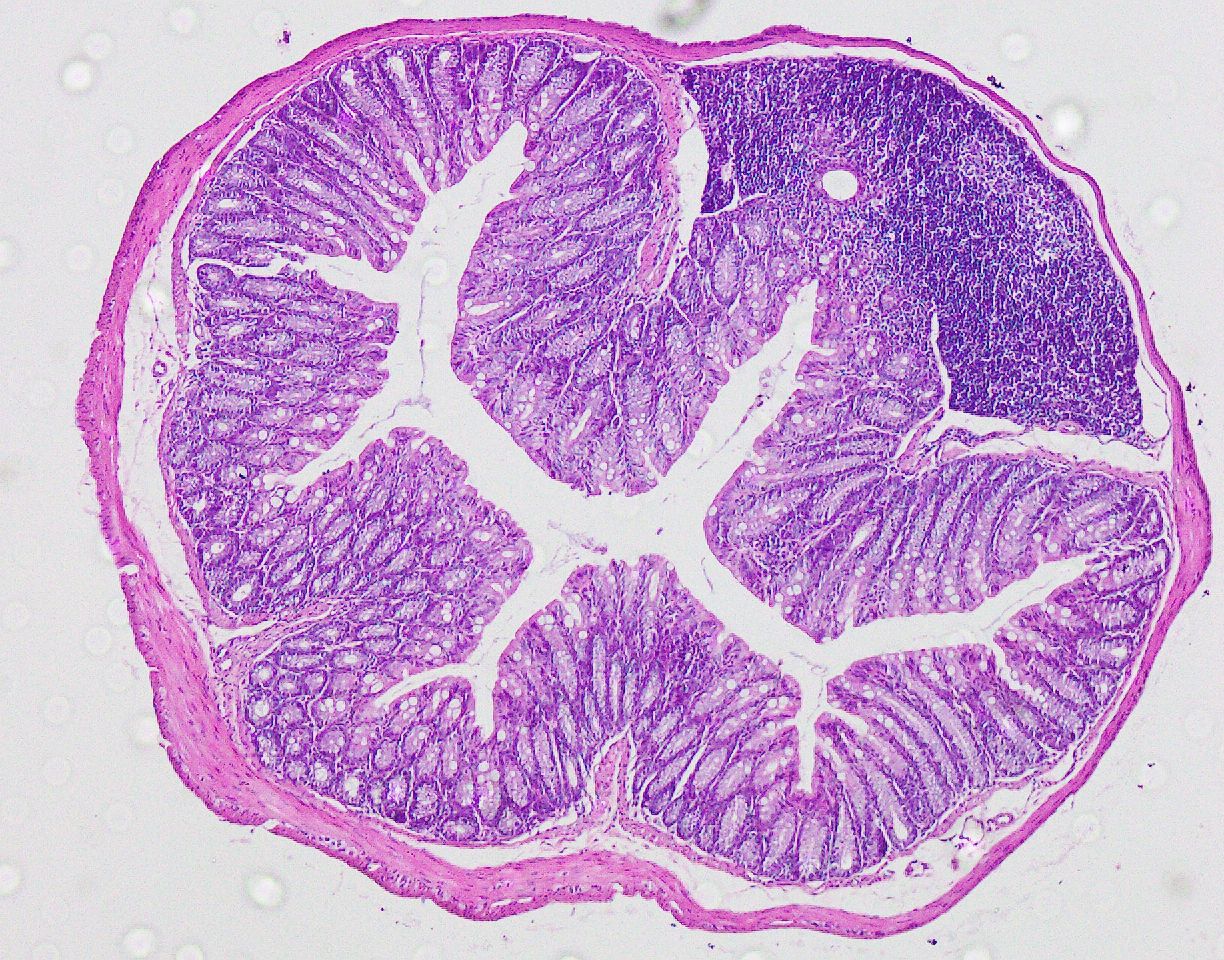

Supplement: Supplementary file 2 [file DataSheet2.zip › HE/fig7 Control 200μm.JPG]

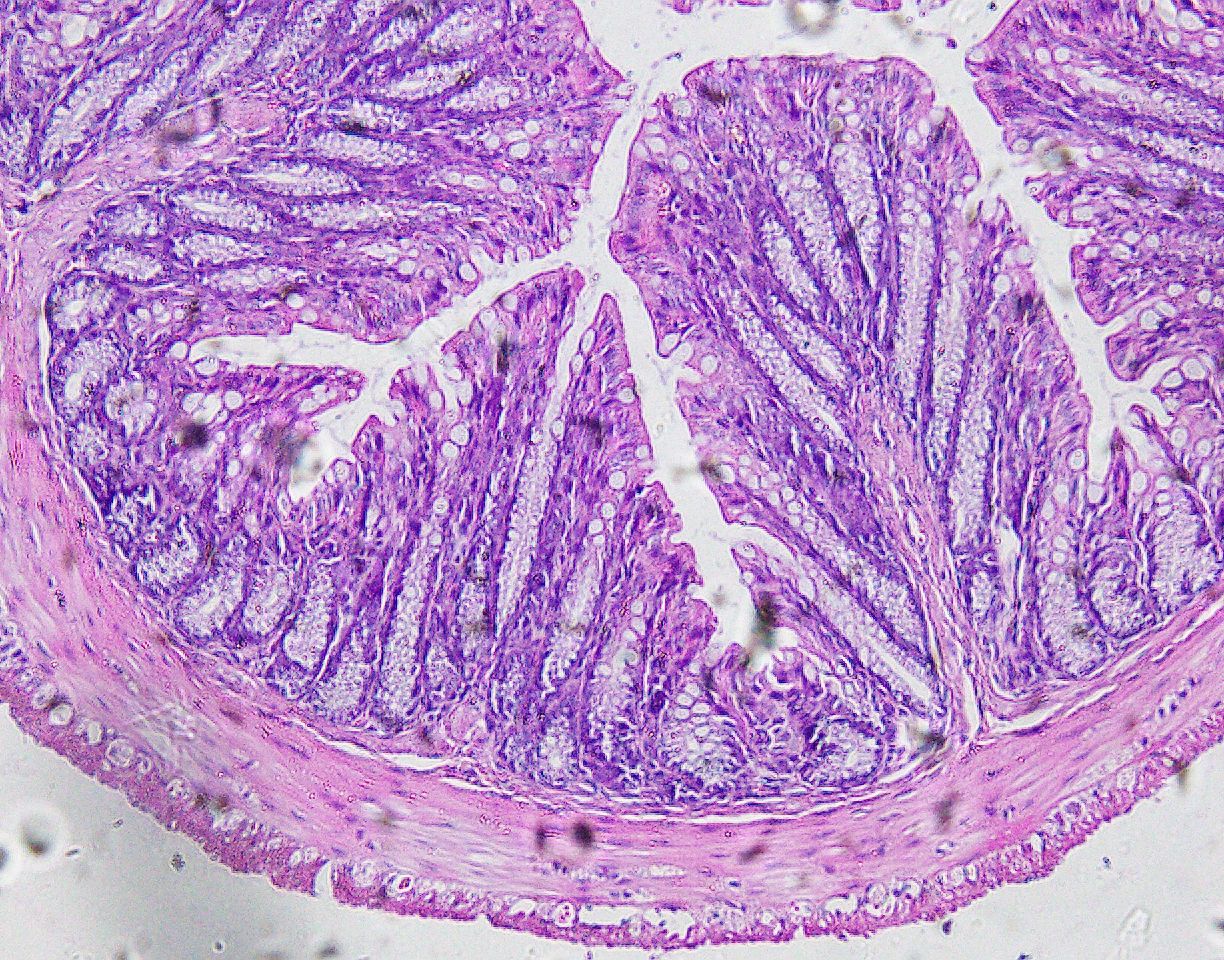

Supplement: Supplementary file 2 [file DataSheet2.zip › HE/fig7 Sham 100μm.JPG]

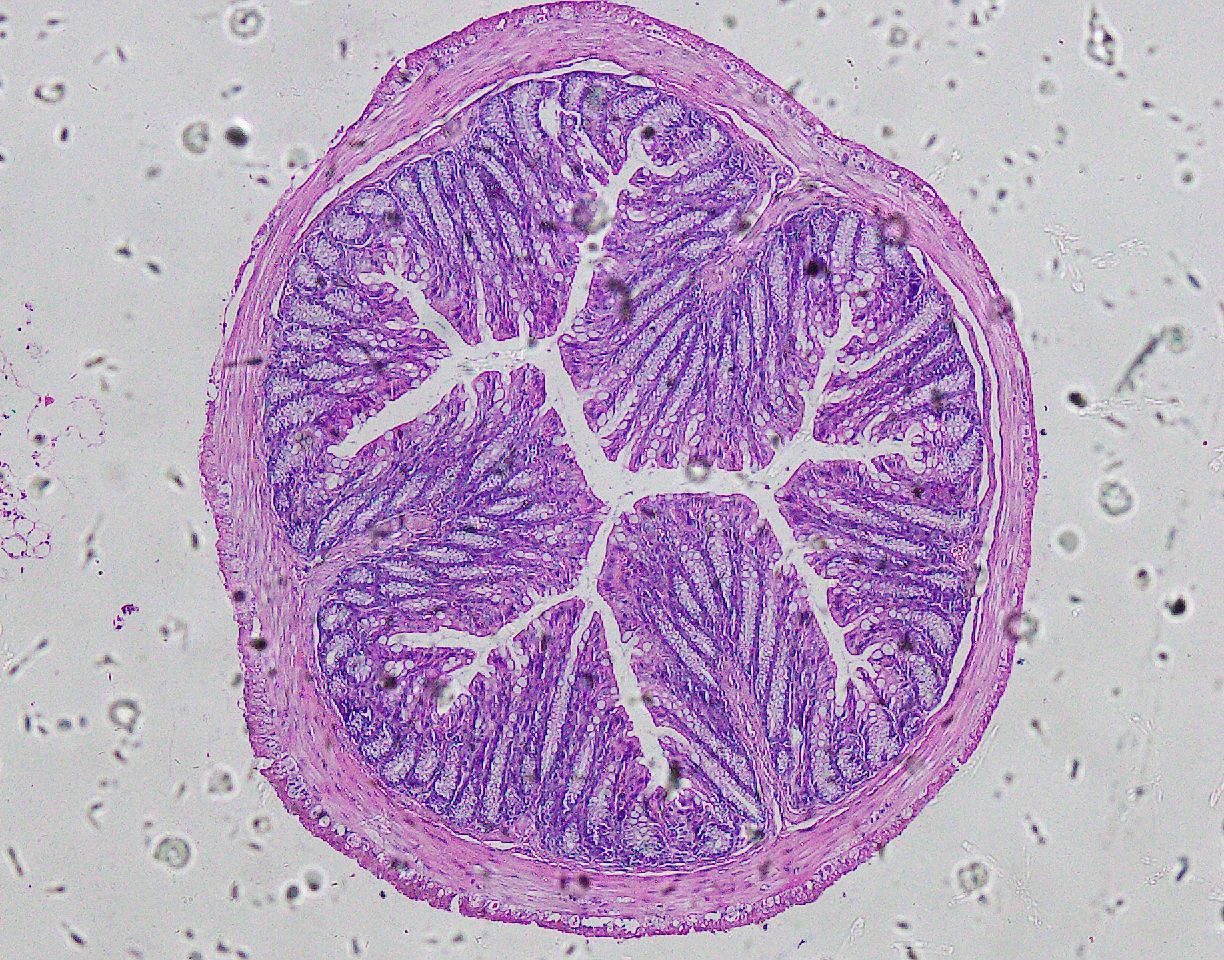

Supplement: Supplementary file 2 [file DataSheet2.zip › HE/fig7 Sham 200μm.JPG]
