# Supplementary material for: Arbutin Ameliorates Murine Colitis by Inhibiting JAK2 Signaling Pathway
Source: Front Pharmacol. 2021 Sep 14;12:683818. doi: 10.3389/fphar.2021.683818 (PMC8477021; doi:10.3389/fphar.2021.683818)
Supplement: Supplementary file 3 [file Image1.pdf]

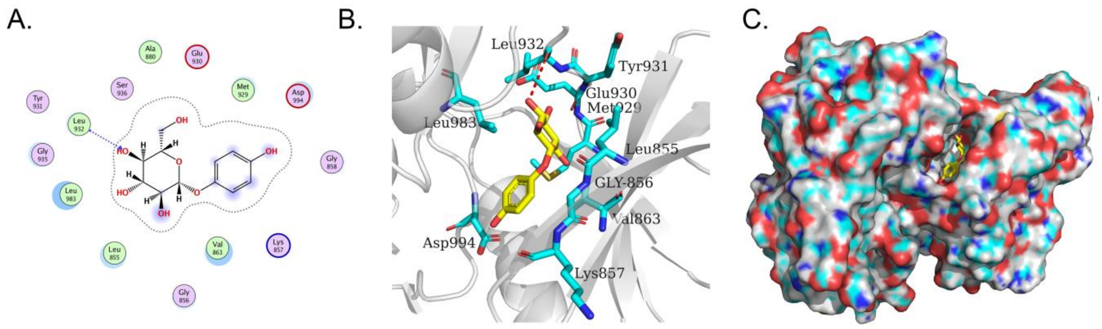

Supplementary figure 1. Molecular docking between arbutin and JAK2 protein. (A) The 2D binding mode of STOCK1N-07141 with JAK2. **(B)** The 3D binding mode of STOCK1N-07141 with JAK2. **(C)** The 3D surface binding mode of STOCK1N-07141 with JAK2. The compound is colored in yellow, the surrounding residues in the binding pockets are colored in cyan. The backbone of the receptor is depicted as gray90 cartoon.
